# Comparison of CpG- and UpA-mediated restriction of RNA virus replication in mammalian and avian cells and investigation of potential ZAP-mediated shaping of host transcriptome compositions

**DOI:** 10.1261/rna.079102.122

**Published:** 2022-08

**Authors:** Valerie Odon, Steven R. Fiddaman, Adrian L. Smith, Peter Simmonds

**Affiliations:** 1 Nuffield Department of Medicine, Peter Medawar Building for Pathogen Research, University of Oxford, Oxford OX1 3SY, United Kingdom; 2 Department of Zoology, Peter Medawar Building for Pathogen Research, University of Oxford, Oxford OX1 3SY, United Kingdom

**Keywords:** dinucleotide, RNA virus, zinc finger antiviral protein, interferon-stimulated gene, interferon, trancriptome

## Abstract

The ability of zinc finger antiviral protein (ZAP) to recognize and respond to RNA virus sequences with elevated frequencies of CpG dinucleotides has been proposed as a functional part of the vertebrate innate immune antiviral response. It has been further proposed that ZAP activity shapes compositions of cytoplasmic mRNA sequences to avoid self-recognition, particularly mRNAs for interferons (IFNs) and IFN-stimulated genes (ISGs) expressed during the antiviral state. We investigated whether restriction of the replication of mutants of influenza A virus (IAV) and the echovirus 7 (E7) replicon with high CpG and UpA frequencies varied in different species of mammals and birds. Cell lines from different bird orders showed substantial variability in restriction of CpG-high mutants of IAV and E7 replicons, whereas none restricted UpA-high mutants, in marked contrast to universal restriction of both mutants in mammalian cells. Dinucleotide representation in ISGs and IFN genes was compared with those of cellular transcriptomes to determine whether potential differences in inferred ZAP activity between species shaped dinucleotide compositions of highly expressed genes during the antiviral state. While mammalian type 1 IFN genes typically showed often profound suppression of CpG and UpA frequencies, there was no oversuppression of either in ISGs in any species, irrespective of their ability to restrict CpG- or UpA-high mutants. Similarly, genome sequences of mammalian and avian RNA viruses were compositionally equivalent, as were IAV strains recovered from ducks, chickens and humans. Overall, we found no evidence for host variability in inferred ZAP function shaping host or viral transcriptome compositions.

## INTRODUCTION

Cellular innate immune responses are typically targeted toward pathogen-associated molecular patterns (PAMPs) that enable cellular recognition pathways to differentiate infecting agents from cellular components. Typical PAMPs displayed by viruses that are recognized in vertebrate cells include cytoplasmic double stranded RNA or DNA sequences, nonmethylated CpG dinucleotides in viral DNA and abnormally terminated (uncapped and nonpolyadenylated) RNA sequences (Randall and Goodbourn 2008; Wilkins and Gale 2010). Alternatively, viruses may be recognized through their possession of conserved virus capsid structures, such as the nucleocapsids of retroviruses targeted by TRIM proteins, or complexes formed during virus budding by tetherin (Nisole et al. 2005; Sawyer et al. 2007; Hotter et al. 2013; Neil 2013). Recently, selective binding of RNA sequences enriched for CpG dinucleotides by zinc finger antiviral protein (ZAP) was described (Takata et al. 2017), and this form of compositionally abnormal RNA represents a potential PAMP. ZAP binding triggers antiviral mechanisms that potently restrict replication of RNA viruses and retroviruses with elevated frequencies of CpG in their genomes (Ficarelli et al. 2019a,b; Miyazato et al. 2019; Odon et al. 2019; Kmiec et al. 2020; Goonawardane et al. 2021). ZAP-dependent restriction of viruses with elevated frequencies of UpA has been similarly characterized (Odon et al. 2019; Goonawardane et al. 2021) and may be mediated through overlapping pathways. While the structural basis of ZAP-mediated restriction of CpG-high sequences has been partially characterized, there appear to be multiple mechanisms downstream that restrict virus replication, including a dependence on TRIM25 (Choudhury et al. 2017; Li et al. 2017; Takata et al. 2017; Zheng et al. 2017; Ficarelli et al. 2019a), activation of the nuclease KHNYN (Ficarelli et al. 2019b), a potentially noncanonical activation of oligoadenylate synthetase and its downstream RNaseL RNA degradation pathway (Odon et al. 2019), or through effects on translation initiation from the bound RNA template (Bick et al. 2003; Zhu et al. 2012; Tang et al. 2017).

The ability of ZAP to identify and restrict replication of viruses with high CpG and UpA frequencies is contingent on the pervasive suppression of both dinucleotides in vertebrate cellular mRNA sequences that remain below the ZAP “radar.” Frequencies of CpG in vertebrate mRNAs range from 20%–80% of expected frequencies based on the frequencies of their component bases (Bird 1980); suppression is particularly pronounced in mammals and birds and in sequences with a low G + C content (Simmen 2008). Methylation of vertebrate genomic DNA and mutational loss of methylated CpG (Beutler et al. 1989; Fryxell and Zuckerkandl 2000) (and a consequent secondary loss of UpA (Duret and Galtier 2000) may account for dinucleotide suppression within encoded cellular genes. However, there are consistent differences in degrees of suppression between coding and noncoding RNAs and relationships between CpG and UpA frequencies with G + C composition that imply the existence of further selection mechanisms against both dinucleotides in sequences expressed as RNA in the cytoplasm (Simmonds et al. 2013).

While the phenomenon of ZAP-mediated restriction of viruses mutated with high CpG or UpA inserts is well characterized, the functional role of this pathway in restricting virus replication in nature is less well understood. Most vertebrate RNA viruses display similar degrees of CpG and UpA suppression to those of the host cells they infect (Karlin et al. 1994; Rima and McFerran 1997; Lobo et al. 2009; Simmonds et al. 2013; Sexton and Ebel 2019) rendering the pathway seemingly pointless in the majority of cases. The main exceptions are double-stranded RNA viruses, such as members of the *Reoviridae*, that show little suppression of CpG and may therefore be able to avoid ZAP-mediated restriction through currently uncharacterized evasion or shielding mechanism.

The other prominent exceptions are members of the vector-borne *Alphavirus* genus of the family *Togaviridae*, such as Sindbis virus, which shows much higher CpG frequencies than other RNA viruses of similar G + C content. This has been shown to enable ZAP-mediated restriction of wild-type virus in mammalian cells (Bick et al. 2003; Zhang et al. 2007; Law et al. 2010). We have proposed incomplete suppression of CpG may represent an “adaptive compromise” for these and other groups of vector-borne viruses, such as flaviviruses (Fros et al. 2021). While the replication of high CpG mutants of Zika virus was restricted in mammalian cell culture, they replicated to higher titers in insect cell culture and showed greater systemic spread and excretion in saliva in experimentally infected mosquitoes (Fros et al. 2021). Flaviviruses that replicate in insects only (insect-specific flaviviruses; ISFs) show no equivalent CpG suppression (Krupovic et al. 2018), mirroring the absence of methylation and CpG suppression in insect and many other arthropod groups. This supports the idea that dual host-specificity places conflicting evolutionary pressures on genome composition, a problem that may be resolved differently in different virus groups. Alphaviruses appear to have adopted a CpG high genome to facilitate replication in mosquitoes, while vector-borne flaviviruses may be better optimized for replication in vertebrate hosts. The remarkable observation that the apparent host range restriction of ISFs to insect cells may be overcome in mammalian cells by knocking out ZAP expression (Colmant et al. 2021) underlines the involvement of this pathway in determining host range. A specific role of ZAP in preventing transmission of arthropod viruses that do not suppress CpG to mammals and birds remains a speculative possibility; if so, it would be a protective mechanism that vector-borne viruses have been able to breach, albeit at some evolutionary cost.

It has been previously proposed that in addition to controlling RNA virus replication, ZAP and allied antiviral restriction pathways may play roles in host gene regulation (Greenbaum et al. 2009a). For example, the greater than expected suppression of CpG frequencies in human type I interferon (IFN) genes (encoding IFN-α, IFN-β) was proposed as a mechanism to enhance expression of these critical antiviral proteins (Greenbaum et al. 2009a). The IFN-inducibility of the long isoform of ZAP (Schwerk et al. 2019; Nchioua et al. 2020) may indeed induce a hostile internal milieu in cells challenged by virus infection where high levels of ZAP expression may suppress global gene expression and bias gene expression toward genes with oversuppressed CpG frequencies, such as those for IFNs and thereby enhancing the cellular anti-viral state. Shaw et al. (2021) have indeed recently proposed that interferon stimulated genes (ISGs) may also be considered as part of a compositionally distinct “interferome” with suppressed frequencies of CpG that might enable prolonged gene expression after induction of ZAP. Experimentally, the hypothesis is difficult to substantiate since CpG is almost universally suppressed in vertebrate transcriptomes and ZAP or ZAP-like proteins have been detected in the wide range of vertebrates investigated to date (Daugherty et al. 2014). Recently, however, it was reported that functional suppression of viruses with high CpG frequencies was impaired in cells expressing a range of avian-derived ZAP genes compared to those expressing human ZAP (Gonçalves-Carneiro et al. 2021). The possibility that avian cells may not suppress replication of high CpG RNA sequences as effectively as mammalian cells was supported by Greenbaum and colleagues (Greenbaum et al. 2009b), who observed that CpG representation in the “Spanish flu” pandemic strain of influenza A virus (IAV) genomes reduced systematically in the decades after 1918 following its zoonotic transfer from birds to humans. This change was proposed to have occurred in response to a more restrictive environment in mammalian cells mediated at least in part by a functional ZAP.

If avian ZAP shows a reduced ability to recognize and suppress expression of mRNAs or inhibit replication of RNA viruses with high CpG RNA sequences, then these functional differences should drive differences in genome compositions between viruses adapted for replication in mammals and birds. Such differences may extend to host-encoded ISG and IFN gene compositions. In the current study, we have investigated whether restriction of the replication of high CpG mutants of influenza A virus and of enterovirus 7 replicons varied between cell lines derived from different avian and mammalian species. The possibility that differences in CpG or UpA-mediated restrictions may influence host transciptome composition was then investigated in large scale compositional analyses of cellular mRNA sequences of different hosts, and targeted comparison of gene compositions of ISGs and different classes of interferons. While CpG-mediated restriction was variable in cell lines from different avian species (in contrast to mammalian cells), these differences had no detectable impact on host transcriptome compositions nor on dinucleotide representations in interferon genes or ISGs expressed on viral infections. The similarity in CpG and UpA dinucleotide frequencies of IAV strains infecting birds and humans similarly provided little evidence that dinucleotide content is associated with mammalian and avian host range adaptation of RNA viruses.

## RESULTS

### Functional investigation of CpG- and UpA-mediated restriction of RNA virus replication in avian and mammalian cell lines

Effects of CpG and UpA dinucleotide modification on the replication of echovirus 7 (E7) were investigated using a previously described replicon construct (Fros et al. 2017). In the E7 replicon, the structural genes are replaced by a luciferase reporter gene that is cotranslationally expressed with downstream nonstructural genes that replicate the replicon. While the E7 replicon can be attenuated through modification of the coding sequence, we inserted nontranslated sequences in the 3′ untranslated region to ensure that any effects on replication did not originate through unintended effects of dinucleotide frequency modification on translational efficiency. In the current study, mutants of the E7 replicon were generated through insertion of a WT E7 sequence, and corresponding CpG-H and UpA-H mutants where the region was modified to elevate CpG or UpA frequencies. A second UpA-high mutant was used where a longer region of UpA-high sequence was inserted (2xUpH-A). As controls, we included a codon scrambled (CDLR) sequence where native dinucleotide frequencies were maintained, and a further construct in which all CpG dinucleotides were removed from the inserted sequence along with the majority of UpA dinucleotides (cu mutant).

Addition of WT sequences in this nontranslated region of the replicon had little effect on replication, while insertion of sequences with elevated CpG or UpA frequencies lead to a ZAP-dependent attenuation of replication as previously described (Odon et al. 2019). The effects of compositional modification on replication in the chicken-derived DF-1 cell lines was investigated by transfection of 50 ng of in vitro transcribed RNA of WT and mutants of the E7 replicon and monitoring replication at 6 h post-infection as previously described (Fros et al. 2017). The experiment was performed in parallel with control human cells A549 and a CRISPR-derived ZAP knockout (k/o) A549 cell line, B6 used in previous ZAP functional assays (Odon et al. 2019).

**FIGURE 1.**
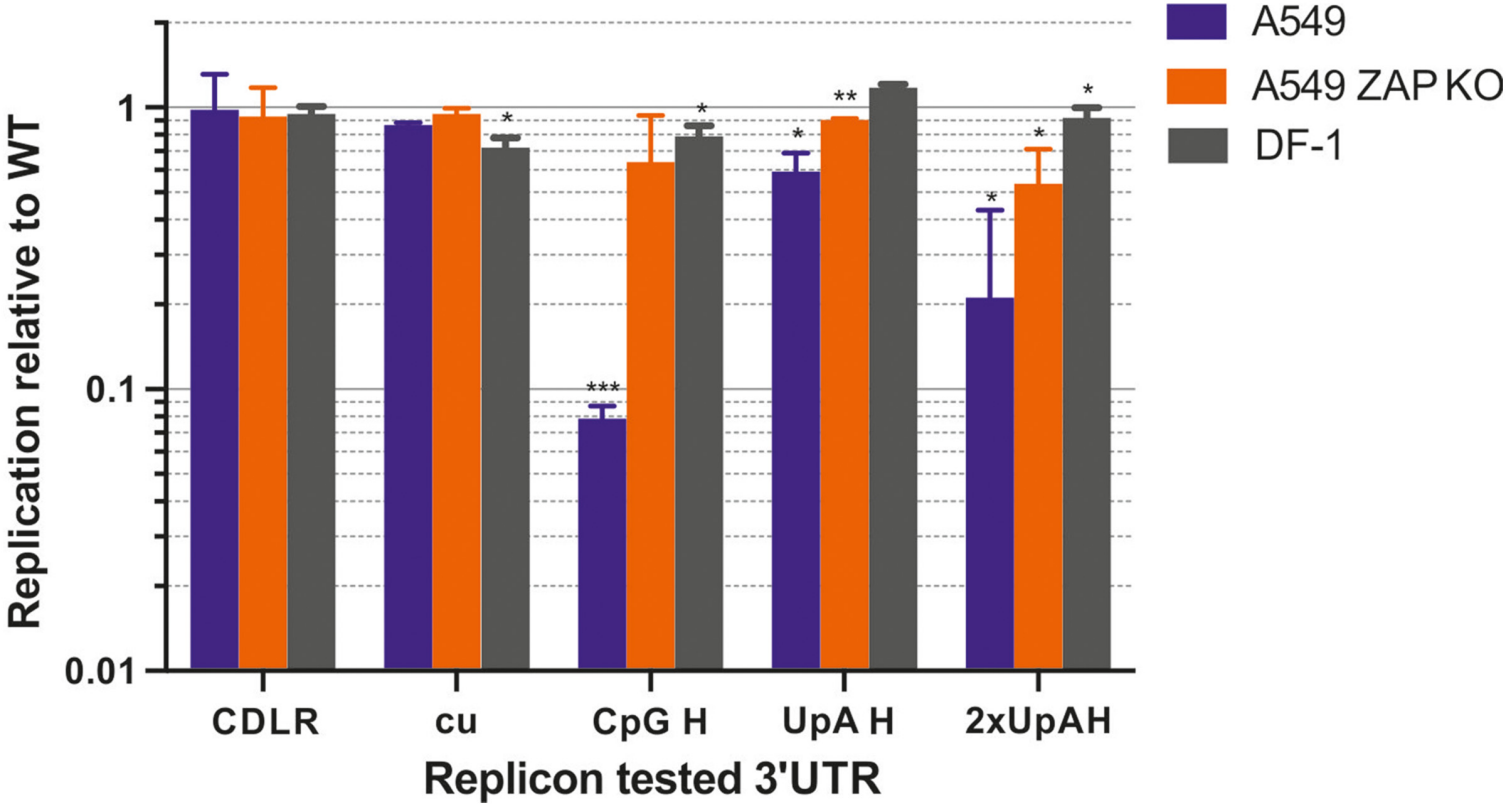
Replication of E7 replicons in control A549 and chicken DF-1 cell line. Cells were transfected with 50 ng/well of in vitro transcribed RNA of E7 replicon with a 3′UTR variant WT, permuted (CDLR), CpG low (cu) and elevated of CpG (CpG-H) and UpA (UpA-H) and assayed for luciferase expression at 6 h post transfection. Relative replication in control human cell lines A549, A549 ZAP k/o, and DF-1 cells. Significance of differences from WT replication were calculated by two-tailed paired *t*-test; asterisks show significance values as follows: (***) *P* < 0.001, (**) *P* < 0.01, and (*) *P* < 0.05

**FIGURE 2.**
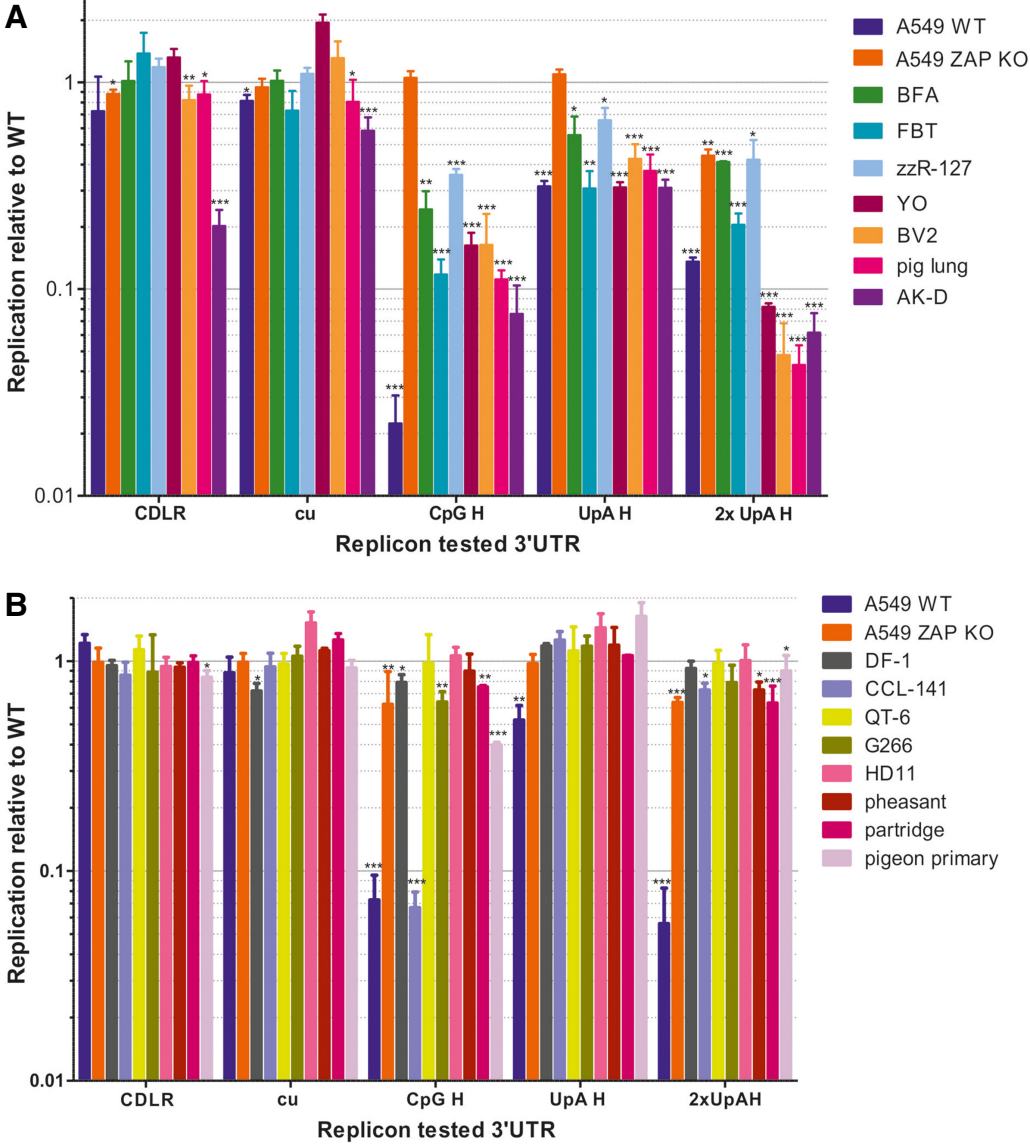
Replication of E7 replicons in a range of (*A*) mammalian and (*B*) avian cell lines. Cells were transfected with 50 ng/well of in vitro transcribed RNA of E7 replicons as described in Figure 1. The bar heights depict mean replication relative to the WT replicon in each cell line; the data were derived from three biological replicates; error bars show standard deviations. Significance of differences from WT replication were calculated by two-tailed paired *t*-test; asterisks show significance values as follows: (***) *P* < 0.001, (**) *P* < 0.002, and (*) *P* < 0.033. Abbreviations: mammalian cell lines: BFA: bovine fetal aorta (host species *Bos taurus*); FBT: fetal bovine turbinate; zzR-127 (*Bos taurus*): goat fetal tongue cell line; YO: derived from a hybrid myeloma YB2/3HL (*Capra hircus*); BV2: microglial cells (*Mus musculus*); AK-D: fetal cat lung (*Felis catus*). Avian cell lines: DF-1: chicken embryo fibroblast (*Gallus gallus*); CCL-141: duck embryo (*Anas platyrhynchos*); QT6: Japanese quail fibrosarcoma (*Coturnix japonica*); G266: male zebra finch (*Taeniopygia guttata*); HD11 chicken macrophage-like (*G. gallus*); primary cells from pigeon, partridge, and quail.

The CpG-H replicon showed a 10-fold reduction in replication compared to WT in the control human A549 cell line as previously observed (Fros et al. 2017) and this attenuation was reversed in the ZAP k/o cell line (Fig. 1). Attenuation of the UpA-H and 2xUpH-A replicon mutants and reversion in the k/o cell line were similarly observed. In contrast to A549 WT cells, minimal or no attenuation of the CpG-H or either UpA-H replicon was evident in the chicken DF-1 cell line. To investigate whether the DF-1 cells typified avian antiviral responses to E7 and the extent to which cell lines from different mammalian species might vary, we transfected E7 replicons into a range of avian and mammalian cell lines and determined the extent of attenuation of CpG-H and UpA mutants by reference to replication levels in the WT E7 replicon (Fig. 2). As controls, mutants with 3′UTR sequences scrambled by CDLR or with minimized frequencies of CpG and UpA (cu) were constructed and tested in parallel. As previously described, replication of both control replicons was comparable to WT replicons in A549 cells and in A549 ZAP k/o cells; replication in each mammalian cells line was comparable in each of the mammalian cells lines with the exception of the replication of the CDLR mutants in AK-D cells although the cu mutant replicated to similar levels to those seen with the WT replicon. In contrast, the CpG-H replicon was attenuated in all mammalian cell lines tested: rat (YO), mouse (BV2), goat (zzR-127), bovine cell lines (FBT and BFA), cat (AK-D), and primary pig lungs showed reductions in replication of six- to 10-fold compared to the WT replicon (Fig. 2A). There was a comparable reduction in the replication of the UpA-high replicon mutants, with the double UpA mutant showing levels of attenuation comparable to that of the CpG high mutant. Attenuation of both UpA-H mutants was reversed in the ZAP k/o cell line, indicative of a direct or indirect role of ZAP in their attenuation.

Parallel assay of a range of avian cell lines derived from members of the Galliformes, Anseriformes, Passeriformes, and Columbaves orders revealed markedly contrasting restriction of the CpG-H mutant (Fig. 2B). Representing Galliformes, cell lines included the macrophage-like HD11 cell line from chicken, QT6 from quail and primary cell lines from pheasant and partridge. We also evaluated replication in the duck embryo cell line CCL-141 (Anseriformes) and zebra finch G266 cell line (Passeriformes) and pigeon primary cells (Columbaves). There was little or no attenuation of CpG-H or UpA-H replicons in any of the galliform-derived cells (DF1 fibroblasts and HD11 macrophages from chicken, QT6 cells from Quail, or primary embryo fibroblasts from pheasant or partridge). Similarly, minimal inhibition was detected in the zebra finch cell line G266. However, the duck CCL-141 cell line and pigeon primary cells exhibited complete or partial restriction of the CpG-H E7 replicon construct. Of note and in marked contrast to mammalian cells, none of the avian cell lines showed any substantial restriction of either UpA-H mutant. This included the duck CCL-141 cell line in which the CpG-H mutant was severely attenuated (Fig. 2B).

**FIGURE 3.**
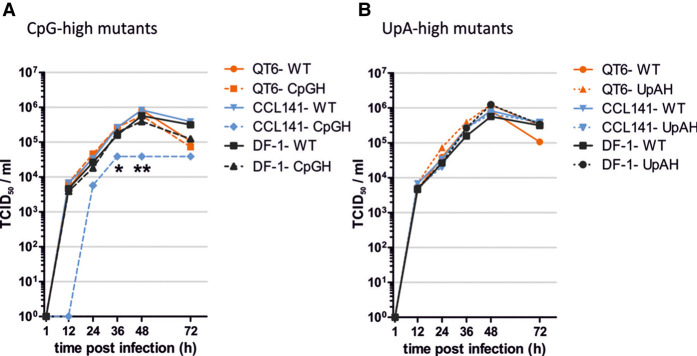
Comparison of the replication kinetics of WT IAV with mutants with elevated (*A*) CpG and (*B*) UpA dinucleotide frequencies in segment 4. Replication was assayed in mutants in the avian cell lines QT6 (Japanese Quail fibrosarcoma—*Coturnix japonica*), DF-1 (chicken embryo fibroblast—*Gallus gallus*) and CCL-141 (duck embryo—*Anas platyrhynchos*). Cells were infected at an MOI of 0.001, and at specified times supernatant was collected and titrated by infectivity using A549 ZAP k/o cells—TCID_50_ values shown on the *y*-axis. Data points represent the mean of two biological replicates; the reduced replication in the replication of the CpG-H compared to WT was significant in unpaired value *t*-test at 24 h and 36 h time points, (*) *P* = 0.03, (**) *P* = 0.04, and by two-way ANOVA (*P* = 0.022). No other comparisons of mutant (CpG-H or UpA-H) or time point with WT were significant at the *P* = 0.05 level.

To verify the apparent difference in restriction of the E7 replicon mutants between avian species in a virus system, we used an established reverse genetics system for influenza A virus to create mutants of IAV with compositionally modified segment 4 sequences encoding hemagglutinin (HA). A total of 100 additional CpG or UpA dinucleotides were introduced without modifying the amino acid sequence of the encoded HA protein sequence. Modifications were made only in the mid-section of this segment to avoid potential unintended effects of mutagenesis on viral transcriptional and packaging signals at the segments ends (whole segment sequences are listed in Supplemental Data Table S1). The replication kinetics of WT and mutants of IAV from cells infected at low MOI (0.001/cell) were comparable in the galliform QT6 and DF-1 cell lines from quail and chicken, respectively (Fig. 3A). However, while replication of WT IAV in the duck-derived cell line, CCL-141, was comparable to that of other cell lines, the CpG-H mutant was substantially attenuated, producing approximately one log less infectious virus at each collection point over the 72-h time course, significant at the 24- and 36-h timepoints. Consistent with the data obtained with the E7 replicon, no attenuation of the UpA mutant IAV was observed (Fig. 3B).

**FIGURE 4.**
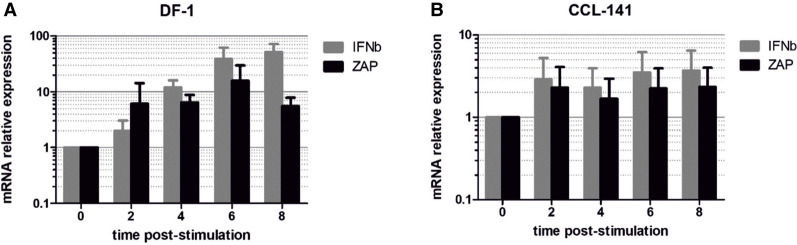
Induction of IFN-β and ZAP after poly(I:C) stimulation. RNA extracted from cell lysates of (*A*) DF-1 chicken and (*B*) CCL-141 duck cell lines at different time points post-stimulation was quantified for ZAP and IFN-β mRNA expression by qRT-PCR. Data shows mean fold-change in expression relative to unstimulated control.

To investigate whether the lack of CpG-mediated restriction observed in chicken cells was the result of reduced expression or a failure of induction of ZAP on infection, we determined mRNA levels of ZAP and IFN-β in DF-1 cells after poly(I:C) stimulation and compared responses with those in CCL-141 cells (Fig. 4). In both cell lines, poly(I:C) treatment induced expression of IFN-β indicating that cells were responsive and both cell lines up-regulated ZAP mRNA at 8 h post-stimulation. Based on this comparison, the failure of restriction of CpG-high mutants in DF-1 cells cannot be attributed to a lack of cellular up-regulation of ZAP expression. Consistent with this finding, there was a comparable absence of CpG- or UpA-mediated restriction in DF-1 cells pretreated with poly-I:C (Supplemental Data Fig. S1).

### Adaptive evolution of avian ZAP

**FIGURE 5.**
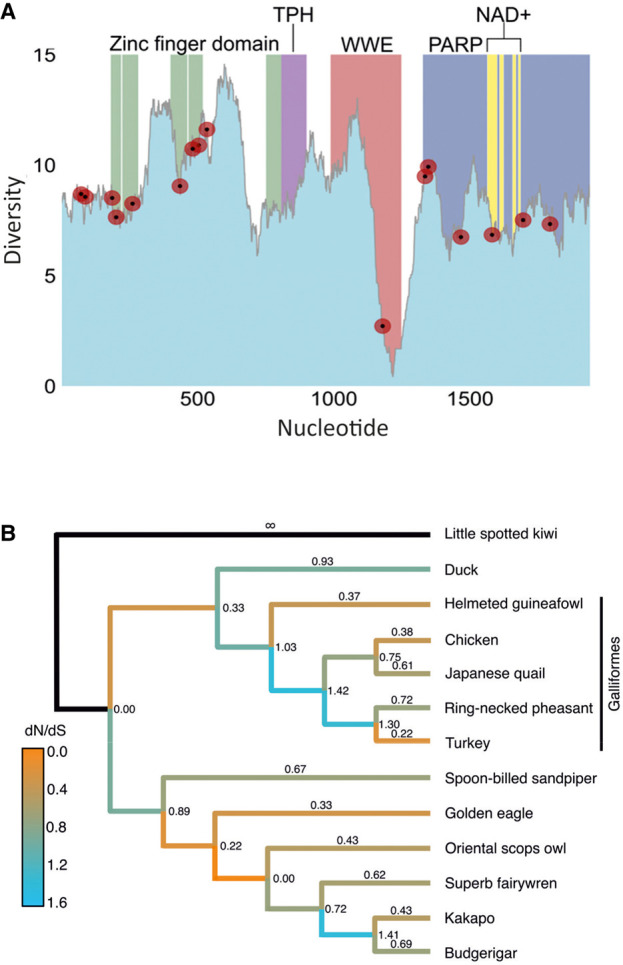
Diversity and positive selection analysis of avian ZAP. (*A*) Using an alignment of 13 avian ZAP sequences (Supplemental Data Table S4A) and following trimming to remove a region of no homology between the fourth and fifth zinc-finger motifs, a sliding-window analysis of sequence diversity was conducted using 100 bp windows and 1 bp increment size. Diversity was calculated as the average pairwise number of variant sites per 100 bp window. The four domains of ZAP are shown in colored blocks, including the NAD+ binding sites in the PARP domain (yellow). Superimposed in red points are positively selected sites as determined by M2a in PAML. Nucleotide position refers to the position in the alignment following trimming—original coordinates of the chicken sequence can be found in Supplemental Data Table S4B. (*B*) Using the same alignment, the free-ratios model in PAML was implemented to determine along which branches positive selection has been strongest. Each branch is labeled with its respective dN/dS value, and branches are colored according to dN/dS.

Evidence for rapid adaptive changes in ZAP gene organization and function in the primate lineage has been proposed to indicate the importance of ZAP in host cell defense (Moriya et al. 1998). The variability in ZAP function in avian cell lines observed in the current study may reflect effects of different selection pressures on the evolution of the avian ZAP gene locus. To investigate whether (and along which lineage) avian ZAP has undergone potential virus-driven adaptive evolution, site and branch models were implemented in PAML to detect positive selection. The majority of sites in the alignment were found to be under purifying or neutral selection (87.93%, M2a; 84.17%, M8; Supplemental Data Table S5), while a smaller proportion (12.07%, M2a; 15.83%, M8) were determined to be under positive selection. Positive selection was highly significant (*P* ≈ 0) for all site model comparisons, compared to the null hypothesis of purifying and neutral evolution. Using the Bayes Empirical Bayes procedure, codons evolving under positive selection were identified (*n* = 16 for M2a, *n* = 27 for M8; Supplemental Data Table S5). Codons under positive selection tended to cluster around the zinc-finger domain (*n* = 7, of which six were found within the CCCH motif itself) and the PARP domain (*n* = 6). Furthermore, one positively selected site, 527V, was found within the NAD+ binding site in the PARP domain (Fig. 5A).

To determine which lineage(s) have undergone positive selection in avian phylogeny, the same alignment was analyzed using the “free-ratios” test in PAML, which assigns a ω value for each branch in the tree. The most striking burst of positive selection was found at the base of the Galliformes, consistent with a change of ZAP function in this lineage (Fig. 5B). Weaker instances of positive selection were found in the Neoaves, but purifying selection predominated elsewhere in the tree. As adaptive evolution frequently associates with genes involved in host-viral arms races (Sawyer et al. 2004; Daugherty et al. 2014), the findings are consistent with rapid host evolution of the ZAP gene in response to RNA virus-induced selection of ZAP-associated antiviral functions within the 150 million year period of avian diversification.

### Composition of mammalian and avian interferon-related genes

If ZAP differentially regulated expression of IFNs, ISGs and non-ISGs through differences in gene composition, then there should be differences in their dinucleotide compositions in avian species that possessed or lacked functional responses to high CpG replicons and viruses investigated in the study. Formally, CpG suppression should be more marked in duck transcriptomes compared to those of chicken based on the observed functional differences in ZAP (Figs. 2, 3). It is further possible that genes preferentially expressed during an antiviral response, such as interferon-stimulated genes and type 1 interferons, may be under more stringent selection against CpG and UpA as they may need to function in cells with elevated levels of ZAP expression.

**FIGURE 6.**
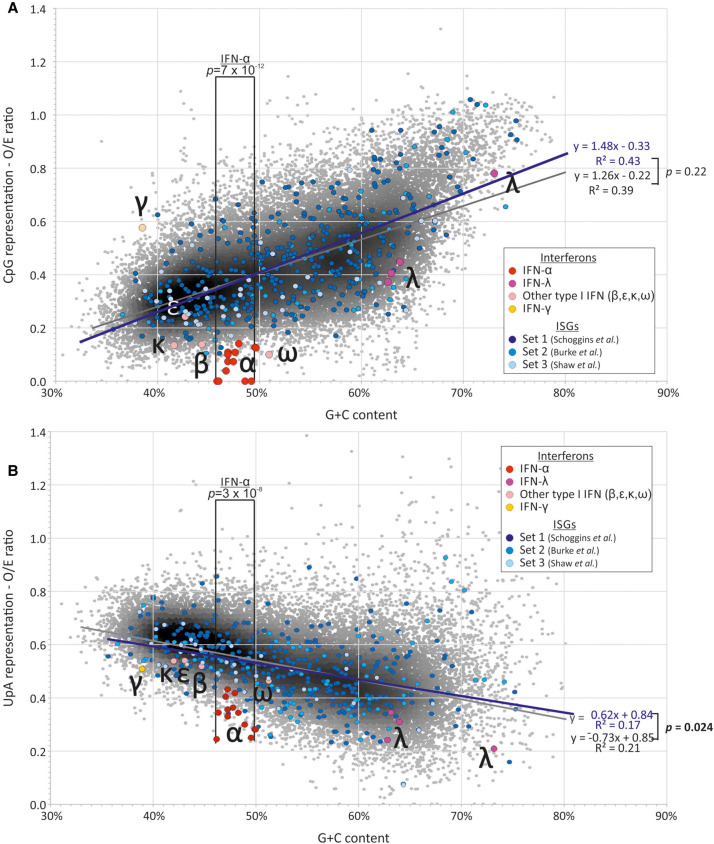
Comparison of dinucleotide frequencies of human ISGs and IFN genes with the bulk human mRNA transcriptome. A comparison of (*A*) CpG and (*B*) UpA dinucleotide representation in human ISGs and interferon genes with the bulk human mRNA transcriptome (of coding sequence lengths ≥450 bases). CpG and UpA representations (observed frequency/frequency expected base on mononucleotide frequencies) of IFN-α paralogs were compared with human mRNA sequences in the G + C content range spanning the former (40.2%–50.0%) using an independent samples *t*-test. CpG and UpA representations in ISGs and mRNA were compared using regression analysis (see Results text; values for individual ISG data sets are shown in Table 2). Sources of ISG sequences: Set 1: Schoggins et al. (2014); Set 2: Burke et al. (2019); Set 3: Shaw et al. (2021).

**FIGURE 7.**
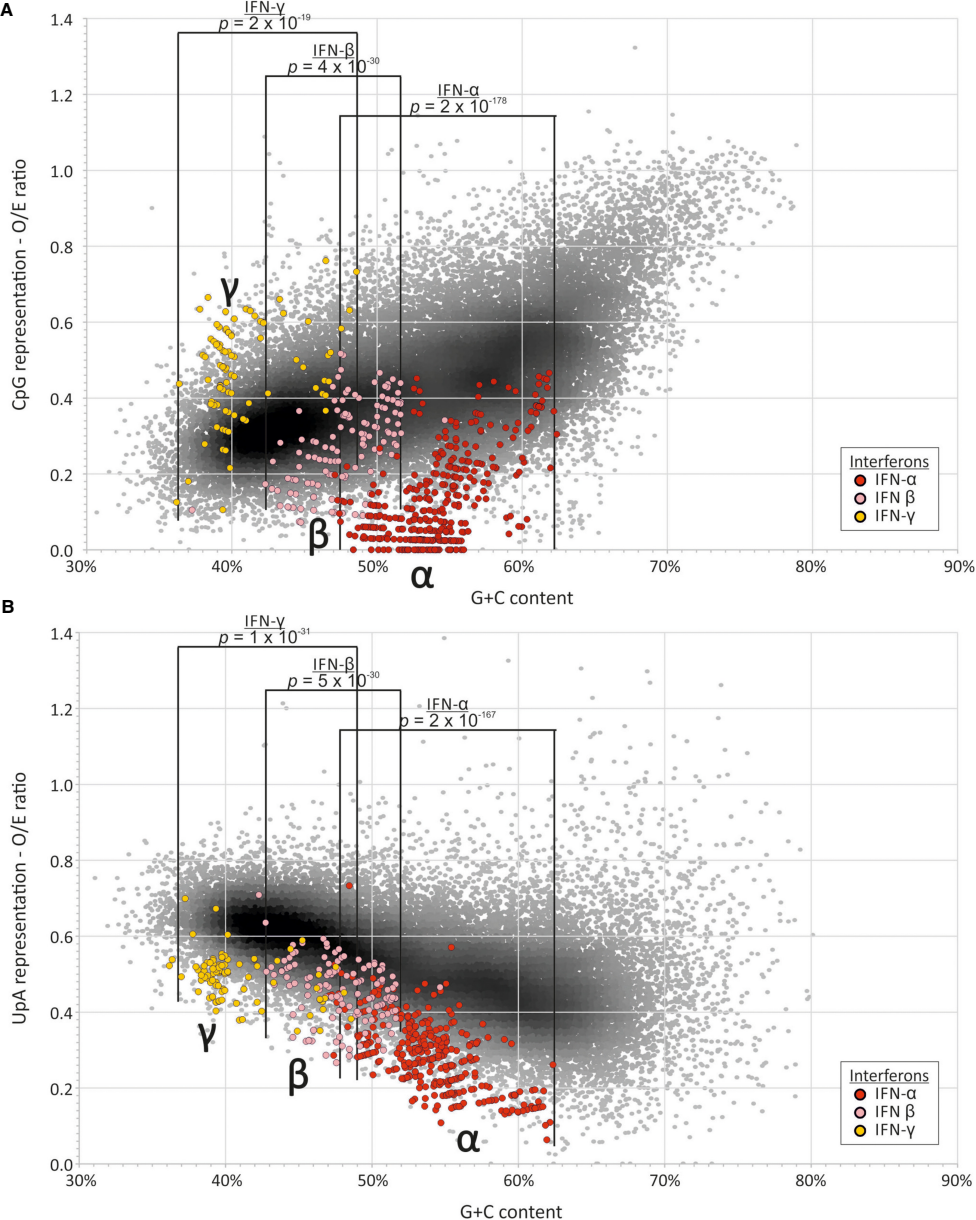
(*A*) CpG and (*B*) UpA representation in mammalian interferon genes. Comparison of dinucleotide representations of IFN-α, IFN-β, and IFN-γ in mammalian species with the bulk human mRNA transcriptome (IFN gene accession numbers and host species are listed in Supplemental Data Table S8). Differences in values over defined G + C content ranges specific for each IFN gene class were analyzed using the independent samples *t*-test.

To investigate these possibilities, sequence compositions of coding region sequences of mRNAs of *G. gallus* (nonrestrictive) and *A. platyrhynchos* (restrictive) were compared with each and with those of human mRNAs using nonredundant RefSeq entries. mRNA sequences from each species showed consistent suppression of CpG and UpA representation, to extents related to their G + C composition (Figs. 6, 7). By linear regression, G + C content was associated with 38%–50% of the variability in CpG representation and 25%–28% of UpA (based on *R*^2^ values). Relationships, expressed as values of m and c in the linear regression relationship (freq. dinucleotide) = (m × G + C content) + c were highly similar between host species, although formal comparison of regression values through calculation of t values (https://stats.idre.ucla.edu/spss/faq/how-can-i-compare-regression-coefficients-between-two-groups/) identified significant differences in trajectories between species (Supplemental Data Table S6A; Supplemental Data Fig. S2). However, while there was functional restriction of high CpG E7 replicons and IAV in the duck-derived CCL-141 cell line but none was detectable in the DF-1 cell line from chickens, cellular mRNA showed a slightly higher CpG representation in duck cells, whereas the expectation would be for the duck transcriptome to show greater CpG suppression if its composition was conditioned by ZAP activity.

Similar comparisons were made between mRNA composition of each species with their corresponding subsets of genes identified as ISGs. For human genes, lists of up-regulated genes on IFN stimulation were obtained from three separate published sources and analyzed collectively (Fig. 6) or individually for composition or regression comparisons (Table 1; Supplemental Data Table S7). As a group, human ISGs showed similar mean values in CpG or UpA representation, and no significant differences from sequences in the wider human mRNA transcriptome (Table 1A). Regression analysis similarly revealed no significant difference in relationships between G + C composition and either CpG or UpA representation (Table 1B). On analyzing individual subsets of ISGs, ISGs reported by Schoggins et al. (2014) and ISGs up-regulated by poly(I:C) treatment (Burke et al. 2019) showed a comparable range of G + C and dinucleotide representations to those of total mRNA sequences (Table 1A). However, the subset of the 50 most up-regulated ISGs reported by Shaw et al. (2021) showed a mean CpG O/E ratio of 0.35, significantly lower than the mean of 0.45 of human mRNAs (*P* = 0.0003). However, the ISGs also showed a lower mean G + C content (49.7% compared to 52.9%; *P* = 0.017). Regressions of this ISG subset and human mRNAs were comparable (*P* = 0.45; Table 1B).

**TABLE 1.**
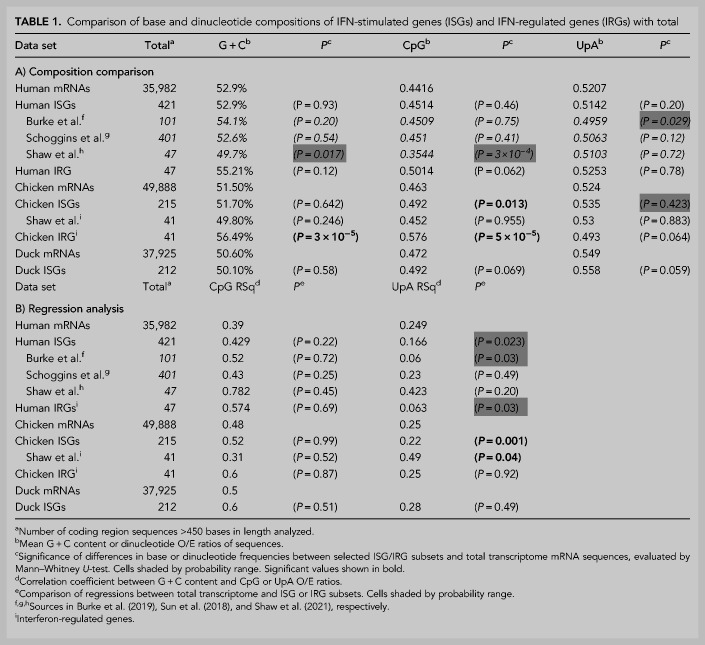
Comparison of base and dinucleotide compositions of IFN-stimulated genes (ISGs) and IFN-regulated genes (IRGs) with total

**FIGURE 8.**
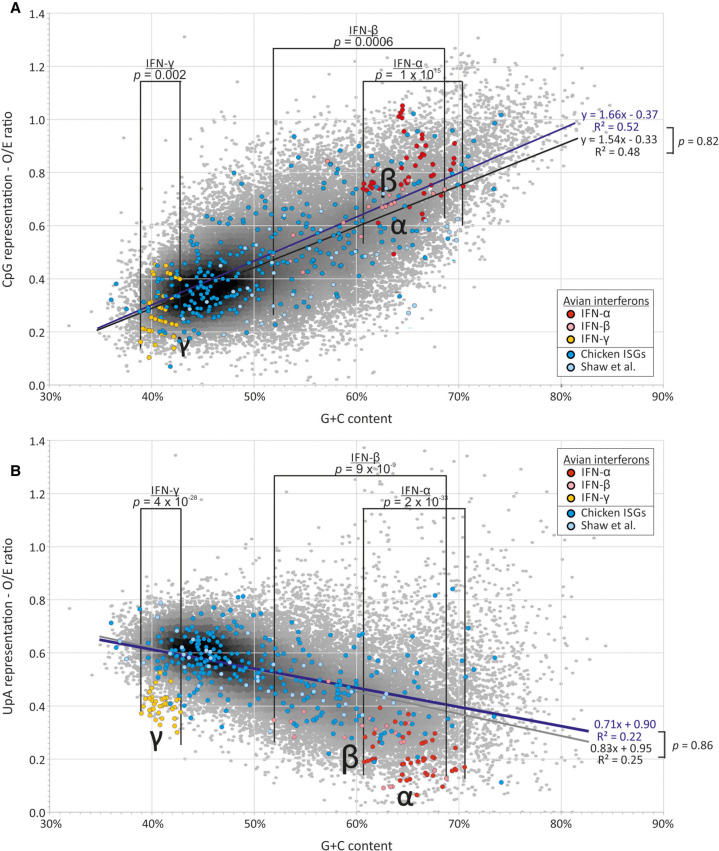
CpG and UpA dinucleotide representation in the chicken transcriptome, ISGs and avian inteferon-α and -γ genes. A comparison of (*A*) CpG and (*B*) UpA dinucleotide representation in chicken ISGs and avian interferon genes (listed in Supplemental Data Tables S8, S10) with chicken mRNA transcriptome sequences (of coding sequence lengths ≥450 bases). Chicken ISGs were identified through homology searching for homologs of human ISGs listed in Supplemental Data Table S8, Schoggins et al. (2014), Burke et al. (2019), and Shaw et al. (2021). Differences in dinucleotide representations over defined G + C content ranges specific for each IFN gene class were analyzed using the independent samples *t*-test.

We obtained similar data for avian ISGs identified as homologs of mRNA sequences of ISGs from the three human ISG data sets (Supplemental Data Table S8). G + C contents of chicken ISGs similarly spanned the range of host mRNA sequences (Fig. 8), with no significant differences in CpG or UpA representation in these or ISG homologs in ducks (Table 1A), apart from a slightly elevated frequency of UpA dinucleotides in duck-derived ISGs. In a separate analysis, the 50 most up-regulated ISGs in chicken (listed in Shaw et al. 2021) similarly showed no significant difference in CpG representation from chicken mRNAs, either by composition comparison or by regression. Both CpG and UpA representations were comparable with host transcriptome and with each other by G + C composition regression analysis (Table 1B; Fig. 7; Supplemental Data Table S6A). Interferon-regulated genes (IRGs) showed significantly higher CpG and UpA compositions, but also a higher G + C content, and there was no significant difference in their distributions by linear regression comparisons.

In contrast to cellular mRNAs and ISGs, IFN genes (listed in Supplemental Data Table S9) showed often marked dinucleotide frequency differences from the host transcriptome. Formal analysis of these differences was complicated by the restricted ranges of G + C contents compared to that of the host transcriptome. Accordingly, frequency comparisons were performed within the G + C content range of the IFN subset being compared, for example between 40.2% and 50% for the comparison of human IFN-α genes with host sequences (Table 2; Fig. 7).

**TABLE 2.**
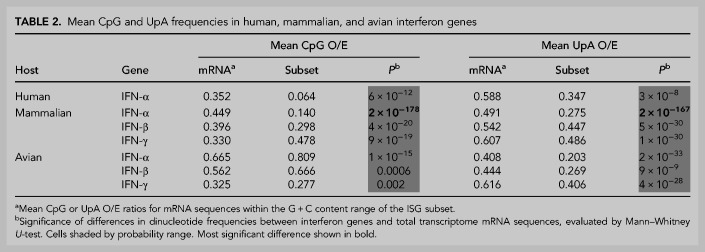
Mean CpG and UpA frequencies in human, mammalian, and avian interferon genes

**FIGURE 9.**
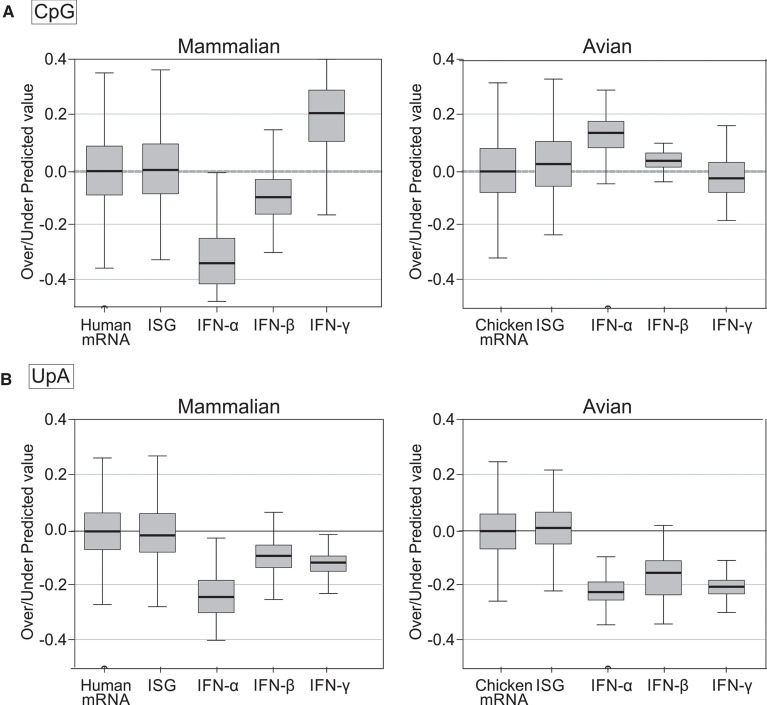
Summary comparison of over- and underpresentation of (*A*) CpG and (*B*) UpA composition in mammalian and avian mRNA sequences and in ISG and IFN subsets. Observed/expected frequencies of CpG and UpA of gene sequences were compared to values predicted from their G + C content using regression formulae for line of best fit for human and chicken mRNA sequences (shown in Figs. 6, 8). Means values and ranges for overrepresentation (positive values) and underrepresentation (negative values) for each sequence are shown in Tukey plots; source listed in Supplemental Data Table S6.

A summary comparison of the degree of over- and underrepresentation of CpG and UpA in mammalian mRNA sequences and those of ISG and IFN subsets was made by calculating the differences in their observed O/E values from those predicted from their G + C content using the previously derived regression formula (1.26 × G + C content) − 0.22 (Fig. 8A) for human mRNA sequences (Fig. 9A; Supplemental Data Table S6B). These values for under- and overrepresentation therefore take the observed relationship between G + C content and O/E dinucleotide frequencies into account. Representations for UpA dinucleotides were calculated similarly (Fig. 9B; Supplemental Data Table S6B). Mammalian IFN-α showed highly suppressed frequencies of both CpG and UpA compared to cellular genes of similar G + C contents (Table 2; Figs. 6–9). IFN-β genes showed lower degrees of suppression of CpG, while IFN-γ gene CpG frequencies were higher than those of the corresponding mRNAs. Both IFN-β and IFN-γ genes showed marked suppression of UpA frequencies.

In contrast to mammalian interferons, avian IFN-α and IFN-β genes (listed in Supplemental Data Table S10) show significantly elevated rather than reduced CpG representations compared to the wider cellular mRNA transcriptomes of chicken (Table 2; Fig. 8A) and duck (Fig. 9A; Supplemental Data Table S6B). In striking contrast, all type I and II IFN genes showed significantly suppressed frequencies of UpA compared to the avian cellular transcriptome (Table 2; Figs. 8B, 9B; Supplemental Data Table S6B), comparable to what was observed in mammalian IFN genes (Fig. 7B, 9B).

### CpG and UpA representation in avian and mammalian RNA viruses

RNA viruses with +strand and −strand genomes infecting vertebrates and retroviruses typically display substantial suppression of CpG and UpA dinucleotide frequencies (Karlin et al. 1994; Rima and McFerran 1997; Simmonds et al. 2013). Representation of both dinucleotides and their G + C dependence are comparable to those observed in cellular mRNA sequences (Simmonds et al. 2013). As ZAP is considered to possess a primarily antiviral function, suppression of CpG and UpA dinucleotides of viruses infecting different hosts may be influenced by potential differences in ZAP activity in different avian and mammalian cells.

**FIGURE 10.**
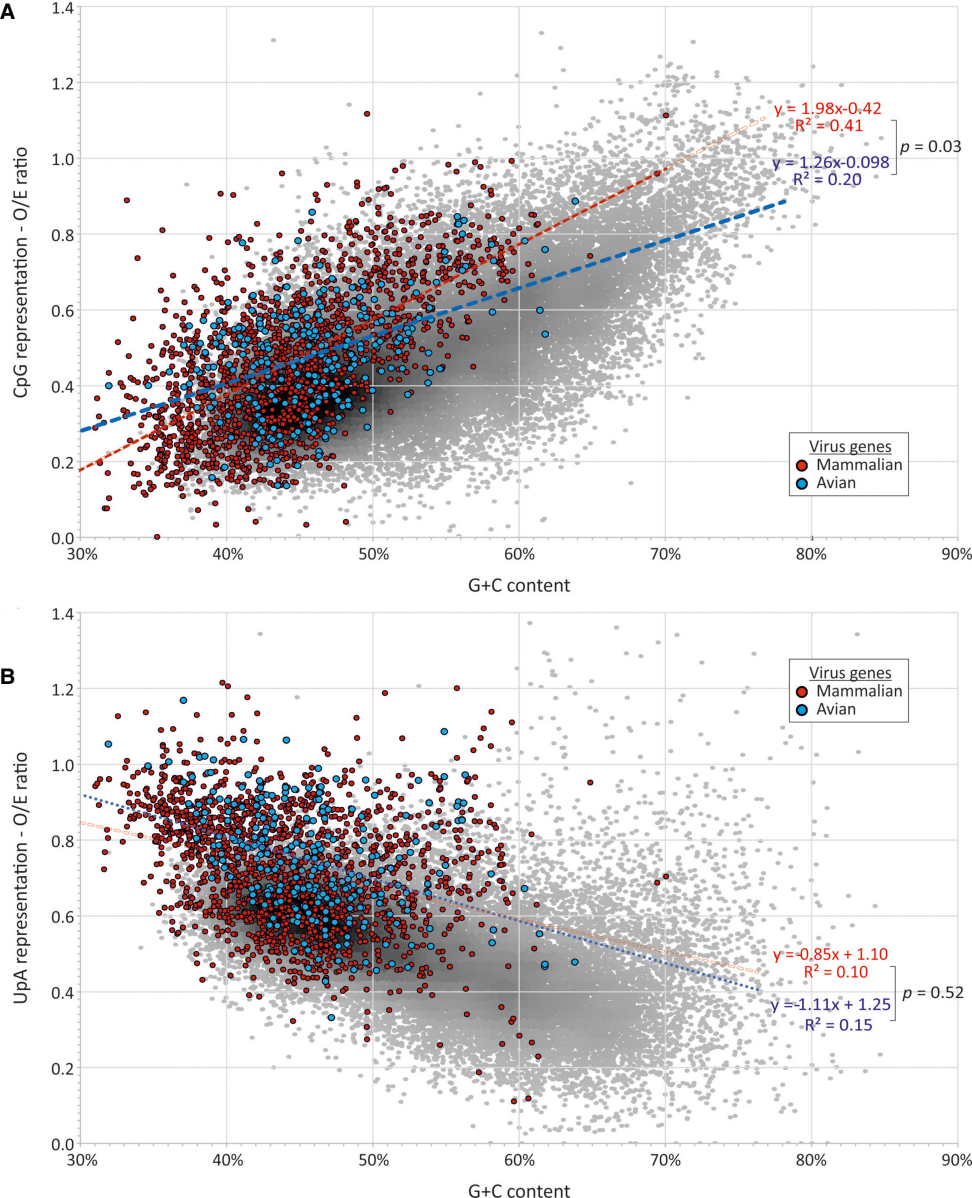
Comparison of CpG and UpA composition in RNA viruses infecting mammals and birds. Observed/expected ratios of (*A*) CpG and (*B*) UpA RNA viruses (listed in Supplemental Data Tables S11, S12) infecting mammals and birds. Ratios have been overlaid on values for avian (chicken) mRNA sequences to provide context. Analysis restricted to coding sequences of length >450 bases.

To investigate this, we collected data sets of RNA viruses from the ICTV virus metadata resource (VMR) representative of each species of + and −strand RNA viruses and retroviruses annotated for mammalian or avian hosts but excluding dual-host viruses such as arboviruses (Supplemental Data Table S11). Genomes were split into component genes and compositional analyses performed on their coding sequences; sequences shorter than 450 bases in length were excluded from analysis. Overall, a total of 1929 genes from 554 mammalian RNA virus genomes and 319 genes from 74 avian viruses drawn from 21 different virus families were compared (Supplemental Data Tables S11, S12). In general, distributions of CpG and UpA frequencies in RNA viruses infecting both mammals and birds were fully overlapping with each other and with dinucleotide compositions of cellular genes (Fig. 10). Although the mean G + C content of RNA viruses was lower than that of human or avian mRNA sequences, suppression of CpG and UpA dinucleotides showed a similar relationship with G + C content as observed in cellular mRNAs. Correlation coefficients and trajectories of G + C/dinucleotide relationships for mammalian and avian RNA viruses were minimally different from each other and comparable to those of avian (Fig. 10B) and mammalian (Fig. 10A) mRNA genes.

**FIGURE 11.**
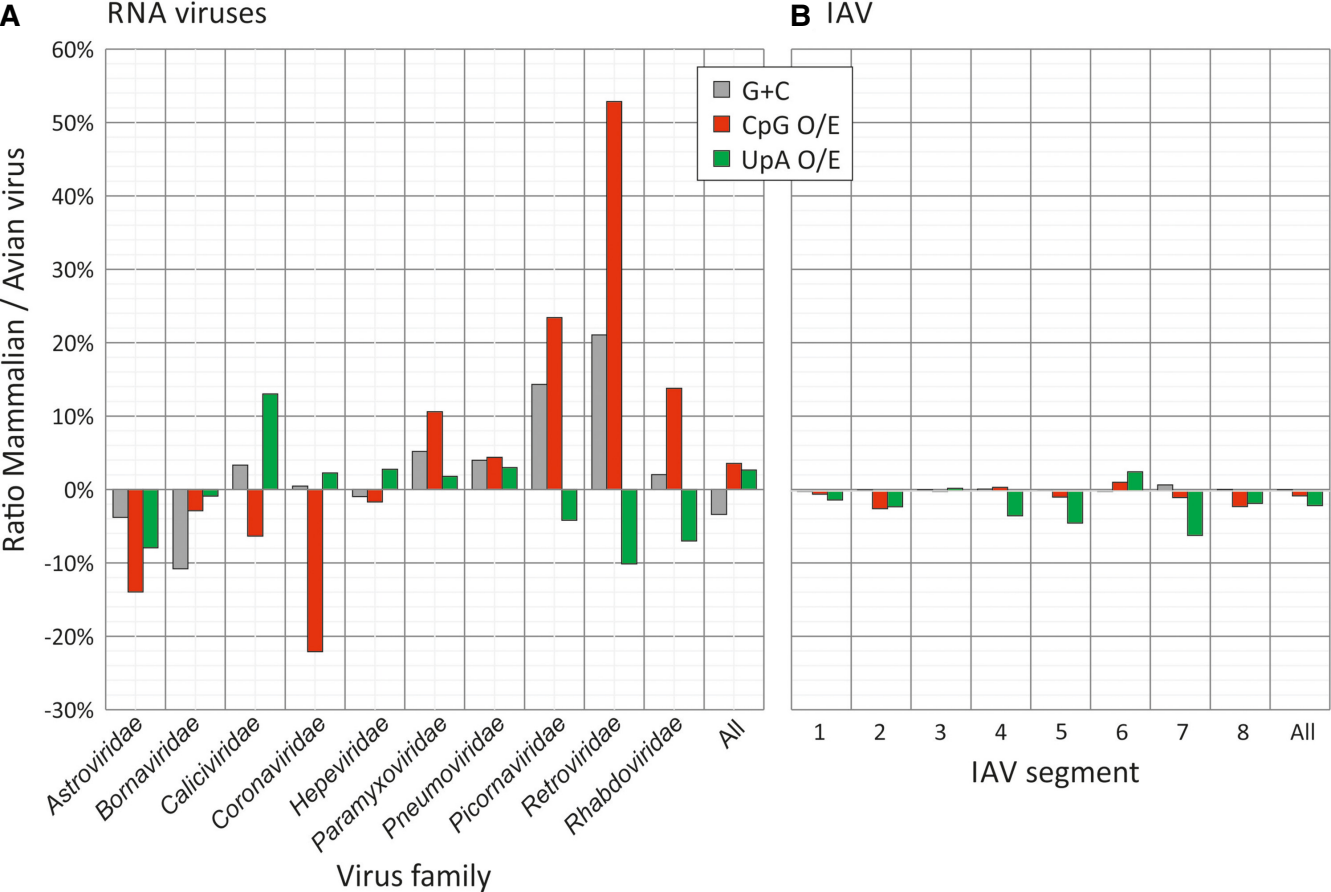
Differences in G + C content and CpG and UpA representation in gene sequences of RNA viruses infecting mammals and birds. Comparison of virus composition between viruses infecting mammals and birds, expressed as fractional differences in G + C content and CpG and UpA dinucleotide representation calculated as f(M)–f(A)/f(All), where f(M) is the mean composition of mammalian viruses, f(A) is of avian viruses, and f(All) is the overall mean. Analyses were performed separately for (*A*) different virus families and (*B*) for different segments of flu strains isolated from mammalian (human) and avian sources (duck, chicken). Further analyses of differences between duck and chicken hosts, analyses where recently zoonotic H5N1 strains have been excluded and CpG and UpA representations calculated independently of protein coding are provided in Supplemental Data Table S15. Source sequences are listed in Supplemental Data Tables S10, S11.

Because viruses assigned to different virus families may show systematic differences in composition (particularly G + C content), another way to compare effects of host is to compare compositions of avian and mammalian viruses assigned to the same family (Fig. 11A; Supplemental Data Table S13A). Differences in G + C content, CpG and UpA representations were observed between the 10 virus families with mammalian and avian members (excluding orthomyxoviruses). However, there was no consistency in whether CpG or UpA were over- or underrepresented in mammalian compared to avian viruses between them; combining the data from all RNA viruses, net differences in all three composition metrics were close to zero.

Influenza A virus (IAV) is an example of a multivertebrate host virus, and serotypes infecting a range of avian and mammalian host species have been extensively characterized genetically. Ducks are regarded as the primary reservoir of IAV worldwide, although the virus will transmit readily to other avian species and to mammals. Adaptation to a new host, such as the H1N1 serotype in human following its pandemic spread from 1918 (Taubenberger et al. 2007) has been proposed to drive reductions of CpG dinucleotide frequencies (Greenbaum et al. 2008, 2009a) potentially associated with a more active ZAP-mediated recognition of avian-adapted viruses (Gonçalves-Carneiro et al. 2021). To investigate whether differences in ZAP function in ducks, chickens and mammalian cells led to systematic differences of CpG and UpA representation, we analyzed dinucleotide compositions in a large data set of representative isolates of IAV with annotated hosts from the Influenza Research Database (mammalian: 2949 isolates, primarily H1N1 and H3N2 serotypes; avian: 6417 isolates; Supplemental Data Tables S14, S15). Coding region sequences in each segment were analysed separately (Fig. 11B).

**FIGURE 12.**
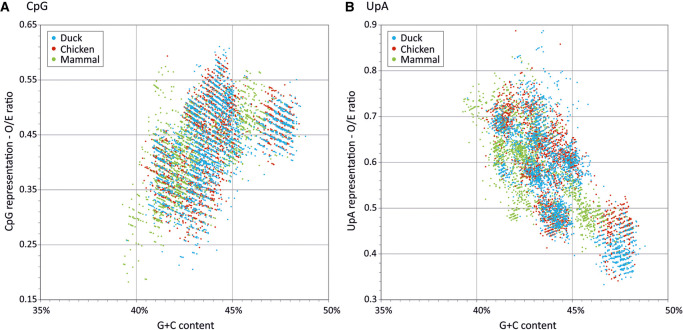
Comparison of (*A*) CpG and (*B*) UpA compositions of mammalian and avian IAV strains. Distributions of CpG and UpA representations plotted against G + C content for IAV strains derived from different hosts; source sequences and serotype totals listed in Supplemental Data Tables S13, S14.

Overall, we found no evidence for any systematic difference in CpG and UpA representation in sequences of IAV strains isolated from humans, chickens and ducks (Fig. 12). As observed in the analysis of different virus families, while there were some differences in CpG and UpA composition of IAV strains infecting humans and birds in different IAV segments, there was no consistent directional trend (Fig. 11B). Combining data for the whole genome, there was a net difference of +1% difference in G + C content, +1.0% in CpG and −1.5% in UpA representation in IAV strains infecting mammals compared to birds (Fig. 11B; Supplemental Data Table S13C). Similar results were obtained on repeating the analysis after exclusion of H5N1 strains (Supplemental Data Table S13D); these have recently zoonotically spread into humans and may therefore preserve genome compositions more typical of avian variants. Finally, the analysis was repeated using CpG and UpA expected/observed ratios that considered only bases that could vary synonymously (corrected frequencies in SSE v.1.4; Simmonds 2012) and which therefore removed potential biases originating from amino acid compositions of viral genes and the presence of fixed dinucleotides within specific codons. This again demonstrated no consistent host-associated differences between IAV segments in their CpG or UpA representations (Supplemental Data Table S16A).

Since ducks and chicken cells varied in their restriction of CpG-high IAV variants or E7 replicons, we performed a further set of comparisons between IAV strains isolated from these two host species (Supplemental Data Table S13B). No consistent differences in G + C contents was observed, although most segments showed marginally lower CpG and UpA representation in duck-derived IAV isolates compared to those of chicken (mean for all segments: −0.64% and −2.0% for CpG and UpA, respectively). Similar findings were obtained using coding corrected dinucleotide representations (Supplemental Data Table S16B).

Overall, this analysis did not show convincing differences in dinucleotide compositions between mammalian and avian RNA viruses, nor between those infecting avian hosts.

## DISCUSSION

This study investigated the extent to which physiological differences in the restriction of CpG compositionally modified viruses, potentially mediated through ZAP, that were observed between cell lines derived from mammals and bird may be reflected in their genome compositions. Of particular interest is the previously proposed effect of ZAP restriction on genes expressed at high level during an antiviral response when ZAP was more active (Shaw et al. 2021). Observed differences in restriction against CpG- and UpA-enriched replicons and viruses between cell lines from these different hosts provided the opportunity to investigate the relationship between ZAP activity and potential differences in depletion of CpG and UpA dinucleotides in cytoplasmic RNA sequences and in the viruses that infected them. While all mammalian cell lines investigated potently restricted CpG-high mutants of E7, avian cells showed variable inhibition of CpG-high E7 and IAV mutants, with restriction most apparent in duck cell lines (order Anseriformes) but not in cells from chicken or other galliforms. Avian cells further differed to mammals by their inability to restrict replication of mutants with elevated UpA frequencies. If CpG and UpA influenced cellular recognition genome composition, then we might expect to observe consistent differences in CpG and UpA representation in host cell transcriptomes, particularly in ISGs and IFN genes where ZAP and potentially a range of other RNA recognizing proteins are up-regulated during an antiviral response. Our findings of compositional equivalence in each of these gene sets in hosts with radically different CpG restriction is reviewed in the light of previously published data on compositional differences in a wide range of genes and host species.

### Host-associated differences in CpG restriction

We documented substantial variability in the abilities of cell lines derived from different avian species to preferentially restrict the replication of high CpG mutants of IAV and the E7 replicon (Fig. 2). It should be noted, however, that the varying permissiveness of the different cell lines for E7 replication may originate from any number of cellular mechanisms and this required us to normalize replication to that of the WT replicon (*y*-axis; Fig. 2). It could be argued that differences in CpG restriction between avian species might originate from intrinsic variability in the propensity of different cell types to express CpG-restriction factors such as ZAP, or in their expression of long or short ZAP isoforms. However, consistent restriction of the CpG-high (and UpA-high) replicons was apparent in all mammalian cell lines used, irrespective of their ontogeny (that included a wide range of cell lines and primary cells from different tissues, including microglia and myeloid cells). In contrast, testing of a similarly wide range of cell types (fibroblasts, various primary cells, fibrosarcoma, and macrophage-like) from birds revealed that most cell lines/types were incapable of restricting (and presumably recognizing) constructs with elevated CpG frequencies. This could be interpreted as evidence for substantially greater variability in ZAP function between avian species. Although we did not compare absolute levels of ZAP expression in the various cell lines, the absence of CpG-restriction in chicken (DF-1) or other avian cell lines was unlikely to be the result of low expression levels of ZAP. ZAP expression in DF-1 was readily inducible by IFN-β (Fig. 4) and by infection of DF-1 cells and of a wide range of other chicken cell types in vivo (Zhu et al. 2019).

The direct experimental format used in the current study differed from the methodology used in a previous investigation of the activity of avian-derived ZAPs, where the replication of CpG modified mutants of HIV-1 was compared in mammalian HEK293T ZAP k/o cells cotransfected human/test species ZAP chimeras and a cognate TRIM25 cofactor required for activity (Gonçalves-Carneiro et al. 2021). Using this human cell transfection system, duck and eagle ZAP/TRIM25-transfected cells mediated marked and selective suppression of CpG-high mutants of HIV-1, consistent with the restriction in CCL-141 (duck) cells observed in the current study and consistent for a potential role of avian ZAP in the restriction of E7 and IAV observed in the current study. However, while chicken ZAP/TRIM25-transfected cells also showed some antiviral activity, restriction was nonselective and similarly active in both CpG-H and WT variants of HIV-1. As our assay readout assigned restriction by normalization to the replication of WT replicon, such nonselective activity in for example, the DF-1 chicken cell line, would have been undetectable. However, if we equate CpG-high/WT replication ratios (Fig. 2) with ZAP/TRIM25 selectivity (Gonçalves-Carneiro et al. 2021), the avian species-associated differences we found were comparable to those identified in the heterologous expression system; specifically, chicken, turkey, and zebra finch showed little CpG selectivity in virus inhibition, in contrast to duck and eagle-derived ZAP/TRIM25.

Additional evidence for host differences in dinucleotide-mediated virus recognition was provided by the absence of restriction of UpA-high E7 replicons (Fig. 2B) and compositionally modified IAV (Fig. 3B) in the duck cell line CCL-141 that was otherwise capable of potent restriction of CpG-high mutants. This contrasts with substantial restriction of the UpA-H double mutant of E7, comparable to that of the CpG-H mutant observed in all mammalian cells (Fig. 2A). As the recognition mechanism of UpA-high mutants remains undetermined and may indeed be mediated through a non-ZAP PRR (Goonawardane et al. 2021), these findings do not necessarily implicate an altered substrate specificity of avian ZAPs. Measurement of the restriction of UpA-high mutants of E7 using the avian ZAP/TRIM25 transfection method would be informative in this respect.

More broadly, the finding of active although variable CpG-mediated restriction in different bird species is consistent with the evidence for positive selection in the ZAP gene in the evolution of birds, comparable to that inferred in primate and wider mammalian evolution (Kerns et al. 2008; Daugherty et al. 2014). The evidence for intense selection at the base of the galliform lineage (Fig. 5) is particularly interesting and may help explain some of the phenotypes seen with cells derived from this group of birds. It is noteworthy that these ZAP-based differences add to other avian lineage-based differences that may explain patterns of viral susceptibility. For example, the loss of RIG-I in galliforms has been attributed to the differences in susceptibility of ducks and chickens to AIV as have the expression and response patterns of other PRRs (Barber et al. 2010; Campbell and Magor 2020). Clearly, ZAP-mediated viral restriction pathways may be an important additional factor defining the outcome of viral infections in different species.

Irrespective of the underlying drivers of variability in CpG restriction, these observations provide an opportunity to investigate whether the observed differences in CpG and UpA recognition play roles in shaping the composition of cellular transcriptomes or of the RNA viruses that infect different hosts. Our analysis of cellular mRNA transcriptomes and the subset of interferon-stimulated genes that may have evolved to function in cells with high levels of ZAP expression, failed to find differences in CpG or UpA suppression between chicken and duck genomes. Collectively, avian genes showed comparable degrees of suppression of both dinucleotides to those observed in mammalian genomes. Our extended analysis of RNA virus genome composition further demonstrated no substantial differences between RNA viruses infecting mammals from those infecting birds, findings that contrast with previous conclusions (Gu et al. 2019; Gonçalves-Carneiro et al. 2021). Of direct relevance to the transmission of IAV between bird species, we found no evidence for systematic compositional differences between IAV strains isolated from ducks and chickens, even though we might hypothesize that the absence of ZAP-mediated discrimination of CpG-high sequences (Gonçalves-Carneiro et al. 2021) in chicken cells might have enabled variants with less CpG-suppressed genomes to have evolved.

More striking is the more general similarity in CpG and UpA representations between mammalian and avian flu strains that conflicts with a previous hypothesis for a strong host-mediated effect on IAV dinucleotide composition (Greenbaum et al. 2008). It was previously found the H1N1 strain that emerged in the 1918 IAV pandemic (“Spanish flu”) from an assumed avian source showed a sustained and decades long systematic reduction in CpG dinucleotide frequencies as the H1N1 strain spread and became established in humans (Greenbaum et al. 2008). This directional compositional change was subsequently interpreted as being indicative of more active ZAP function in human cells after its species jump (Takata et al. 2017; Gonçalves-Carneiro et al. 2021). It was further proposed that recently transmitted IAV strains may be more pathogenic in humans because of their greater CpG frequencies and consequent activation of responses that lead to immunopathology (Greenbaum et al. 2008, 2009a). While superficially attractive as an explanation for the pathogenicity of recently transmitted flu strains, such as those with H5 and H7 HA segments (Lycett et al. 2019), the whole framework might be challenged on several grounds. Firstly, the sequence of the 1918 strain was untypical of avian strains generally, and it has been questioned whether the source of the 1918 pandemic strain was actually avian (Taubenberger et al. 2007). Secondly, the hypothesis for CpG compositional differences arising from different host selection pressures is inconsistent with the finding of no systematic compositional difference between IAV strains recovered from human and ducks (which show CpG-associated restriction) and chickens (that do not restrict) using linear regression that accommodates the effects of G + C content on CpG composition (Fig. 12; Supplemental Data Table S13). Most human strains included in the comparison were the H1N1 and H3N2 serotypes, long established in human populations, while the duck and chicken strains were primarily avian serotypes also with likely long term host associations. The compositional similarities therefore did not arise simply from possible recent cross-species transmission as might be the case for H5N1. The more general grounds for questioning the hypothesis is the previously discussed demonstration of potent ZAP-mediated restriction of high CpG viruses in duck cells (Fig. 2B) and evidence for similar restriction of CpG mutants of HIV-1 by the duck-derived ZAP construct (Gonçalves-Carneiro et al. 2021). As ducks and other aquatic Anseriformes form the principal reservoir of IAV in nature (Webster et al. 1992), it is somewhat problematic to base a hypothesis on an assumption that IAV is less compositionally restricted in avian hosts than in humans.

### Influence of ZAP on host transcriptome composition

The ability of ZAP to recognize and restrict RNA viruses appears to rely on its ability to detect clustered CpG dinucleotides (Takata et al. 2017). ZAP-mediated selection may be the mechanism underlying the previously inferred compositional selection against CpG and UpA suppression and their G + C compositional dependence arising from previous mutational modeling in mammalian transcriptomes (Simmonds et al. 2013). Further indirect evidence for the potential shaping effect of ZAP on host gene composition is provided by analysis of interferon genes. As previously described for human IFN-α genes (Greenbaum et al. 2009a; Li et al. 2009), human and all other mammalian type I IFN genes investigated showed substantially greater CpG suppression than other mRNA sequences (Fig. 7), but a similar dependence on G + C content as found in other mRNA sequences. We also observed much greater suppression of UpA in IFN genes compared together with cellular mRNAs.

Long before the discovery of ZAP-mediated recognition of CpG dinucleotides, Greenbaum et al. proposed that this extreme suppression may enable greater expression of IFNs perhaps at the expense of other cellular genes not engaged in antiviral defense (Greenbaum et al. 2008, 2009a). One might develop the idea further in considering the possible effects of high-level expression of ZAP in interferon-induced cells on virus infection. ZAP overexpression may provide the mechanism for differential gene expression from genes specifically depleted for CpG and UpA dinucleotides. This form of gene regulation may contribute to the antiviral state and is up-regulated by virus infection. Hence, ZAP may enable cellular translation to be diverted toward interferon production as the rest of the of transcriptome is regulated by increased degradation of the virus-infected cells.

Extending this concept to other genes involved in the antiviral response is problematic. It was reported that CpG frequencies were lower in the subset induced by immune stimulation of mouse dendritic cells (Greenbaum et al. 2009a). Similarly, Shaw et al. (2021) have recently demonstrated significantly lower CpG compositions in the 50 most IFN-up-regulated genes in human cells that unregulated or IFN down-regulated genes leading to their conclusion that ZAP may function as a cellular regulator of gene expression, enabling preferential expression of antiviral effector genes during the antiviral state when ZAP expression is up-regulated and functionally more active. However, neither analysis acknowledged the effect of G + C content; reanalysis of the Shaw data set in the current study showed a systematically lower G + C content than the average for cellular mRNAs, and indeed frequencies of CpGs were not systematically different from expected values once this is taken into account by regression analysis (Table 1B). For each subset of human ISGs and their homologs in birds analyzed in the current study, the identified up-regulated genes fall in a similar trajectory to cellular mRNAs, and the comparable CpG and UpA compositions of ISGs from humans (and homologous genes in avian genomes) within the relevant G + C content ranges.

Overall, our extended analyses reach the rather negative conclusion that differences in CpG- and UpA-mediated restriction of virus replication, potentially mediated by ZAP and other PRRs recognizing RNA sequences with abnormal dinucleotide compositions, do not measurably lead to differences in cellular transcriptome compositions of human and avian cells. While there is profound suppression of CpG and UpA frequencies in mammalian type 1 IFN genes, this does not fully extend to those of avian homologs even though there was substantial restriction of viruses and constructs enriched with CpG in duck cells in this and previous studies (Gonçalves-Carneiro et al. 2021). Furthermore, while neither UpA-enriched E7 or IAV mutants were restricted in duck or any other avian cell type, UpA frequencies were globally suppressed in both type I and type II (IFN-γ) IFN genes. There was no systematic difference in CpG and UpA frequency representation between mammalian and avian RNA virus genes, nor between IAV strains recovered from ducks and chickens, despite the functional differences in CpG and UpA restriction in these different hosts. Overall, the study provides no evidence for a role of CpG- or UpA-mediated recognition in differential regulation of gene expression, that might favor ISGs over other cellular genes in IFN-activated cells.

## MATERIALS AND METHODS

### Cell culture provenance and maintenance

As standard, all cells were cultured at 37°C, in 5% CO_2_ and harvested with TryPLE dissociation reagent (Gibco) for 5 min, centrifugation at 1000 rpm for 5 min followed by resuspension in fresh media and reseeding into fresh flasks.

Zebra finch cell Gray 266 (G266) were derived from a naturally occurring tumor of a male zebra finch forehead (Itoh and Arnold 2011) and were kindly donated by Dr. Butter Falk. The cells were maintained in DMEM media supplemented with 10% heat-inactivated fetal calf serum, 2% heat-inactivated chicken serum, 36.7 mmol/L of glucose, and penicillin and streptomycin (Pen/Strep). The cells were replated at a dilution of one-third. Quail cell line QT6, derived from a Japanese quail fibrosarcoma, were purchased from ECACC. The cells were maintained in Ham's 10 buffer supplemented with 2 mM glutamine, 10% tryptose broth, 10% fetal calf serum, and Pen/Strep. The cells were split at 70% to 80% confluency and replated at one in two dilutions.

Human A549 (purchased from ATCC), A549 ZAP^−/−/−^ (CRISPR engineered from WT A549 cells), chicken DF-1, Hek293T, MDBK, zzR-127, BFA, FBT, AK-D, and the primary cell lines for pigeon, partridge, and pheasant were maintained in DMEM- Ham (F12) media supplemented with 10% FCS, 2 mM glutamine, 1% nonessential amino acids, and 1% P/S. For the YO cell line, the media included also 1% sodium pyruvate. They were diluted one in ten when replating.

Duck embryo cell line CCL-141 (ATCC) was maintained in EMEM medium supplemented with 10% FCS, 1% P/S, and subcultured at 70%–80% confluency. They were diluted one in five when replating. FBT and pig lungs’ primary cell lines were cultured in similar media with addition of nystatin.

Primary cell lines from pigeon, partridge, pig lung, and pheasant were supplied by Animal and Plant Health Agency England. zzR-127, YO, and FBT were sourced from the Pirbright Institute cell bank. The AK-D feline cell line was a kind gift from Pr. Ian Goodfellow. BFA was a kind gift from Pr. Rick Randall. DF-1, Hek293T, and MDBK cell lines were from the personal cell collection of the author's cell bank.

### E7 replicon assay

The echovirus 7 (E7) replicon was based on the Wallace strain and modified by insertion of compositionally modified sequences in the 5′UTR as previously described (Fros et al. 2017).

### RNA transcription and transfection

T7 transcription reactions (MEGAscript T7 Transcription Kit, Promega) were assembled according to manufacturer's recommendation with 1 µg of E7 plasmid DNA linearized with NotI or TK-Ren plasmid linearized with XbaI. Following a 2 h incubation and DNase treatment, the transcripts were purified using the RNA Clean and Concentrator Kit (Zymo Research) and the concentration measured using the Qubit Fluorometric Quantitation System (Thermo Fisher Scientific). A total of 50 ng of E7 RNA was transfected to each well together with 5 ng of renilla RNA. The transfection mix was prepared using Lipofectamine MessengerMax (Invitrogen) and OptiMem (Gibco). At 6 h post transfection, the media were discarded and the wells were washed with PBS before adding 50 µL of passive lysis buffer (Promega). Cells were frozen at −20°C to facilitate complete lysis.

### Determination of luciferase activities

Luciferase activities were determined using a GloMax-Multi Detection System (Promega) reader. Each well was injected with dual luciferase reagents (Promega). This method consists of first injecting 50 µL of firefly luciferase reagent to an equal volume of cell lysates. Following a 30 min period for the complete decay of the luminescence, 50 µL of a stop/renilla reagent was injected to measure the renilla luminescence in the same well. Firefly luminescence was normalized using the renilla values. The experiments were carried out in three biological replicates with four technical replicates.

### Reconstitution of chicken cell line with human ZAP

A549 ZAP^−/−/−^ and DF-1 cells were cultured in a 10 cm dish and transfected using LT1 reagent (Mirus laboratory) with 5 µg of huZAP-GFP DNA for 24 h. The cells were transfected with 5 µg of E7 replicons WT, CpG-H and 500 ng of TKRen RNA using Lipofectamine MessengerMAX. Following a 6 h incubation, the cells were trypsinized, resuspended in 1% FCS-PBS buffer, and sorted according to green fluorescence using a BD FACSAria III machine sorter to isolate GFP expressing cells. A total of 30,000 cells were collected in passive lysis buffer in a white 96-well plate, and the luciferase activities were determined as previously described.

### Cloning of IAV mutants

The WSN system was elaborated by Hoffman and colleagues (Hoffmann and Webster 2000) and consists of eight pHW2000 expression plasmids, each encoding one of the viral segments from the H6N1 strain of influenza A virus (IAV A) and driven by a CMV promoter. The system allows manipulation of individual segments of the virus.

The HA segment (segment 4) was selected as it is the most variable segment and contains only one long open reading frame of 1775 bp. The first 171 and last 107 bases were not mutagenized. The mutations introduced into the rest of the HA segment maintained the WT amino acid sequence while increasing the CpG or UpA content and were designed using the SSE software package (Simmonds 2012) (sequences listed in Supplemental Data Table S1). The observed to expected (O/E) ratio of CpG in WT of 0.38 was increased to 1.9 in the CpG high (CpG-H) segment. The mutated segments were ordered from GeneArt (Thermo Fisher). The segments were amplified by PCR using KOD polymerase, creating blunt-end products (Merck) using primers listed in Supplemental Data Table S2. The primers were designed to create overlapping ends with the wild-type sequence of the pHW244-HA vector. The PCR products were DpnI treated and gel purified before proceeding with Gibson assembly.

The Gibson assembly was performed with the NEBuilder HiFi DNA Assembly (NEB) using 50 ng of vector PCR and a twofold excess of insert PCR (CpG-H), following the manufacturer's instructions. The reaction mixes were incubated at 50°C for 30 min. A total of 2 µL of assembled plasmids were transformed in competent TOP10 *E. coli* cells and selected on LB/ampicillin agar plates. The integrity of the mutant plasmids was verified by Sanger sequencing using primer pairs “S4 CGH insert F + R” (Supplemental Data Table S2).

### Rescue of IAV

The transfection reagent was prepared as follows: in one tube, 0.5 µg of DNA for each of the eight plasmids constituting the WSN system were mixed with 25 µL Opti-MEM. For the negative control, one of the plasmids (the HA coding sequence) was omitted. In the second tube, 6 µL of Lipofectamine 2000 were mixed with 125 µL of Opti-MEM. Both tubes were then combined and incubated for 20 min at room temperature.

A confluent T75 flask of HEK 293T cells was trypsinized and harvested by centrifugation and resuspended in 5 mL of DMEM 10% FCS, 1% pen/strep. Five hundred microliters of the cell suspension were then seeded in a six-well plate and mixed with the DNA transfection mix. After 6 h the media were changed to DMEM 0.5% FCS, 1% pen/strep medium. The supernatant was collected following a 48 h incubation period. The viral supernatant was centrifuged at 3000 rpm for 3 min and aliquots were stored at −80°C.

### Plaque assay

To resolve the viral titer of recued viruses, 300,000 MDBK cells were seeded per well of 12-well plates. The following day, a 10-fold serial dilution of the virus stock was prepared with extensive mixing by vortexing between dilutions. The media were aspirated from each well. The cells were washed with PBS, and each well was inoculated with 150 µL of diluted virus. The plates were incubated at room temperature for 1 h with regular rocking for viral absorption. The inoculum was discarded, and wells were washed with PBS. A 1% low melting point agarose (GeneFlow)/0.5% FCS-DMEM overlay was added to each well, following a 15 min setting time at room temperature; the plates were returned to an incubator inverted for 3 d. Plaques stained with crystal violet were visible as clear spots and counted.

### Replication phenotypes with IAV mutants

DF-1, CCL-141, and QT6 were seeded in six-well plates at 500,000 cells and infected with IAV WT or mutant CpG-H or UpA-H virus at MOI 0.001 for 1 h. The inoculum was discarded and the cells were washed with PBS. The cells were cultured in 0.5% FCS-DMEM culture media. At 1, 12, 24, 36, 48, 60, and 72 h post-infection (p.i.), an aliquot was collected and the virus titer determined by TCID_50_ in A549 ZAP^−/−/−^ cells. This was performed with two independent duplicates for each virus and cell line.

### Quantitative PCR

Cells were stimulated using poly (I:C). DF-1 cells were stimulated with 10 µg/mL of poly (I:C) added to the media, and QT6, pigeon, G266, and CCL-141 cell lines were transfected with 1 µg of poly (I:C) using Lipofectamine 2000. At 0, 2, 4, 6, and 8 h post-stimulation, cells were harvested and RNA was purified using a Total RNA Purification Kit (RNeasy Qiagen). An amount of 80 ng of total RNA was added as template for real-time PCR using QuantiFast SYBR Green Master Mix (Qiagen) in the StepOnePlus instrument (Applied Biosystems). The program for cDNA synthesis and RNA quantitation was as follows: for the reverse transcription step (50°C for 10 min, 95°C for 5 min) and for quantitation of 40 cycles (95°C for 10 sec, 60°C for 30 sec).

Expression of each gene was normalized to an internal control (HPRT1) transcript, and these values were then normalized to the nonstimulated control cells to yield a fold induction value. Primers used for the detection of HPRT1, IFNβ, and ZAP are listed in Supplemental Data Table S3.

### Detection of positive selection in ZAP genes

Avian ZAP (encoded by the *ZC3HAV1* gene) sequences were obtained from Ensembl (Supplemental Data Table S4). Sequences were aligned using Muscle v. 3.8.425 (Edgar 2004) and trimmed to remove the highly diverse region between the fourth and fifth zinc-finger motif. To test for positive selection across sites in the alignment, maximum likelihood analysis of ratios of nonsynonymous to synonymous nucleotide substitutions (dN/dS; ω) was performed with the codeml package of programs in PAML v. 4.9 (Yang 1997, 2007). Various site models were fitted to the multiple alignments: M1a (neutral model; two site classes: 0 < ω_0_ < 1 and ω_1_ = 1); M2a (positive selection; three site classes: 0 < ω_0_ < 1, ω_1_ = 1, and ω_2_ > 1); M7 (neutral model; values of ω fit to a beta distribution where ω > 1 disallowed); M8 (positive selection; similar to M7 but with an additional codon class of ω > 1); and M8a (neutral model; similar to M8 but with a fixed codon class at ω = 1). Likelihood ratio tests were performed on pairs of models to assess whether models allowing positively selected codons gave a significantly better fit to the data than neutral models (model comparisons were M1a vs. M2a, M7 vs. M8, and M8a vs. M8). In situations where the null hypothesis of neutral codon evolution could be rejected (*P* < 0.05), the posterior probability of codons under selection in M2a and M8 was inferred using the BEB algorithm (Yang et al. 2005). In addition, to test for positive selection in different branches of the phylogeny, the “free-ratios” model (which fits one ω for every branch in the tree) was implemented in PAML (Yang 1998).

To calculate the nucleotide diversity in the avian ZAP sequences used for PAML analysis, the alignment was analyzed using the PopGenome package in R (Pfeifer et al. 2014). A sliding window of 100 bp and an increment of 1 bp was used to calculate nucleotide diversity across the alignment, which is expressed as the average pairwise number of variant sites per 100 bp window.

### Analysis of dinucleotide frequencies in host genomes

Nonredundant mRNA sequences of human, chicken, and duck were downloaded from the NCBI database (http://www.ncbi.nlm.nih.gov/gene), with coding region sequences shorter than 450 bases excluded. Mono- and dinucleotide frequencies and ratios of observed dinucleotide frequencies to those expected from mononucleotide composition (G + C content in the case of DNA sequences) were calculated using the program Composition Scan in the SSE package (Simmonds 2012). All statistical analyses were performed using SPSS version 26.

## SUPPLEMENTAL MATERIAL

Supplemental material is available for this article.

## Supplementary Material

Supplemental Material
