# Supplementary material for: Comparison of CpG- and UpA-mediated restriction of RNA virus replication in mammalian and avian cells and investigation of potential ZAP-mediated shaping of host transcriptome compositions
Source: RNA. 2022 Aug;28(8):1089–109. doi: 10.1261/rna.079102.122 (PMC9297844; doi:10.1261/rna.079102.122)
Supplement: Supplemental Material [file supp_079102.122_Supplemental_Material_.zip › Supplemental_Figure_S1.docx]

FIGURE S1

EFFECT OF POLY(I:C) STIMULATION OF DF-1 CELL LINE.

Cells were incubated with 10 µg/ml of poly(I:C) for 2h before transfection of all variants of E7 replicons. Luciferase activities recorded at 6 h were normalised to WT and error bars show standard deviation of 4 replicates; no significant differences were observed between pre-treated and control cells for any replicon.
