# Supplementary material for: Comparison of CpG- and UpA-mediated restriction of RNA virus replication in mammalian and avian cells and investigation of potential ZAP-mediated shaping of host transcriptome compositions
Source: RNA. 2022 Aug;28(8):1089–109. doi: 10.1261/rna.079102.122 (PMC9297844; doi:10.1261/rna.079102.122)
Supplement: Supplemental Material [file supp_079102.122_Supplemental_Material_.zip › Supplemental_Figure_S2.docx]

FIGURE S2

COMPARISON OF LINEAR REGRESSION OF ISG SEQUENCES WITH

CORRESPONDING BULK mRNA SEQUENCES


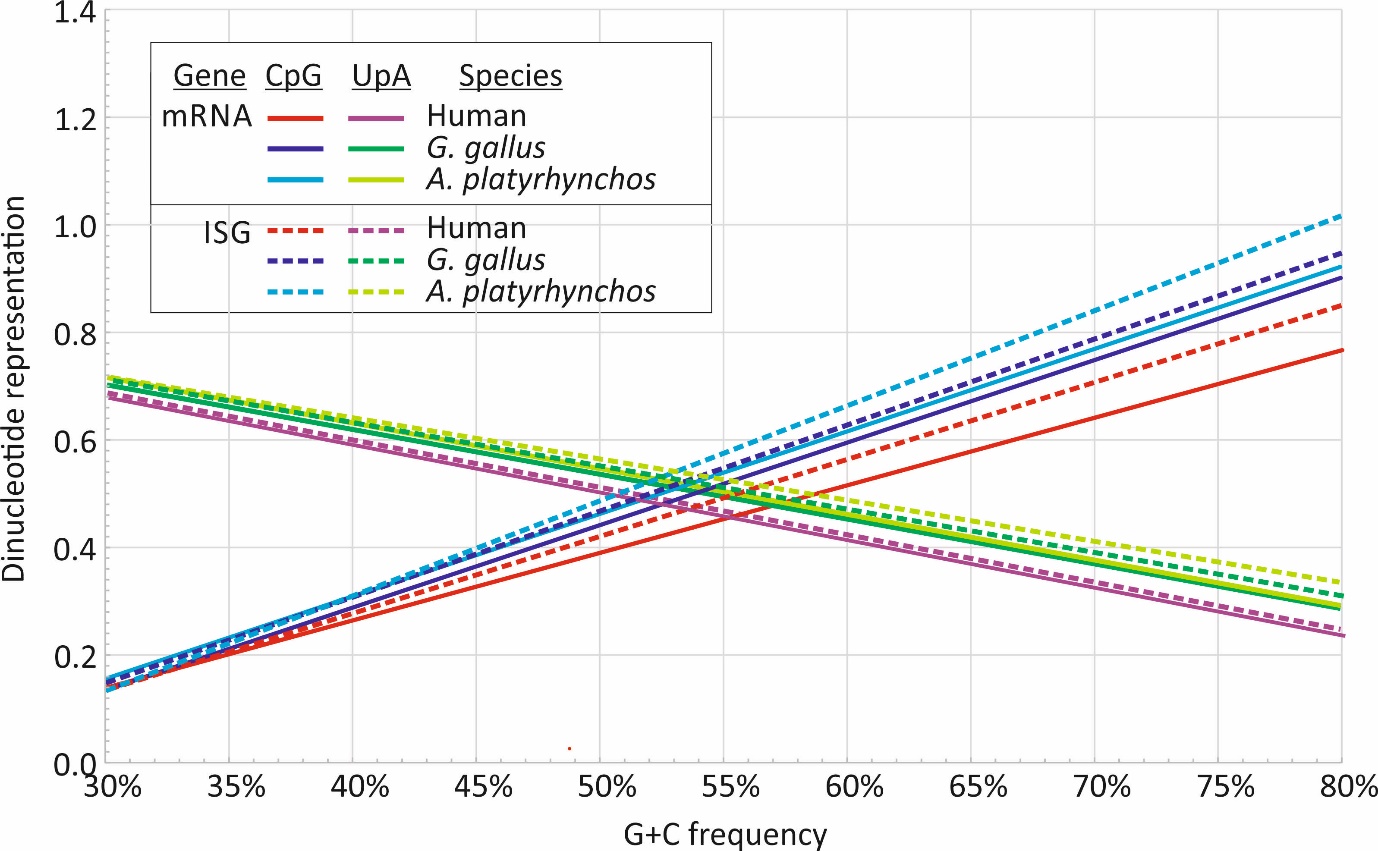


Regression lines for bulk mRNA and ISG sequences from human, chicken and duck genomes. Formulas for regression lines and results of statistical comparisons are shown in Tables S5 and S6.
