# Supplementary material for: Comparison of CpG- and UpA-mediated restriction of RNA virus replication in mammalian and avian cells and investigation of potential ZAP-mediated shaping of host transcriptome compositions
Source: RNA. 2022 Aug;28(8):1089–109. doi: 10.1261/rna.079102.122 (PMC9297844; doi:10.1261/rna.079102.122)
Supplement: Supplemental Material [file supp_079102.122_Supplemental_Material_.zip › Supplemental_Legends.docx]

SUPPLEMENTARY FILES

Supplemental_Table_S1.docx: sequences of the IAV segment 4 (HA) inserts

Supplemental_Table_S2.docx: sequences of primers used to amplify and clone IAV segment 4 mutants

Supplemental_Table_S3.docx: sequences of primers used for qPCR

Supplemental_Table_S4.docx: Sources of ZAP gene (*ZC3HAV1*) sequences used for PAML and diversity analysis

Supplemental_Table_S5.docx: Sites in the ZAP gene (*ZC3HAV1*) gene under selection

Supplemental_Table_S6.docx: Comparison of linear regressions of G+C content with CpG and UpA mRNA frequencies and degrees of under-and over-representation in ISG and IFN gene subsets

Supplemental_Table_S7.docx: Comparison of linear regressions of G+C content with CpG and UpA representation

Supplemental_Table_S8.docx: identified homologues of human ISGs in the chicken genome

Supplemental_Table_S9.docx: Interferon α, β and γ genes analysed from different mammalian species

Supplemental_Table_S10.docx: Avian interferon α, β and γ genes

Supplemental_Table_S11.docx: Total number of virus genomes and component genes analysed for composition

Supplemental_Table_S12.docx: Accession numbers of RNA virus sequences from ICTV VMR

Supplemental_Table_S13.docx: Comparison of CpG and UpA representation in avian and mammalian RNA viruses

Supplemental_Table_S14.docx: Accession numbers of IAV sequences from the influenza research databases

Supplemental_Table_S15.docx: IAV serotypes analysed for dinucleotide composition

Supplemental_Table_S16.docx: Comparison of codon normalised compositions of IAV strains infecting different hosts

Supplemental_Figure_S1.docx: Effect of poly(I:C) stimulation of DF-1 cell line

Supplemental_Figure_S2.docx: Comparison of linear regression of ISG sequences with corresponding bulk mRNA sequences
