# Supplementary material for: Comparison of CpG- and UpA-mediated restriction of RNA virus replication in mammalian and avian cells and investigation of potential ZAP-mediated shaping of host transcriptome compositions
Source: RNA. 2022 Aug;28(8):1089–109. doi: 10.1261/rna.079102.122 (PMC9297844; doi:10.1261/rna.079102.122)
Supplement: Supplemental Material [file supp_079102.122_Supplemental_Material_.zip › Supplemental_Table_S1.docx]

TABLE S1

SEQUENCES OF THE IAV SEGMENT 4 (HA) INSERTS

>WT (full sequence)

ATGAAGGCAAAACTACTGGTCCTGTTATATGCATTTGTAGCTACAGATGCAGACACAATATGTATAGGCTACCATGCGAACAACTCAACCGACACTGTTGACACAATACTCGAGAAGAATGTGGCAGTGACACATTCTGTTAACCTGCTCGAAGACAGCCACAACGGGAAACTATGTAAATTAAAAGGAATAGCCCCACTACAATTGGGGAAATGTAACATCACCGGATGGCTCTTGGGAAATCCAGAATGCGACTCACTGCTTCCAGCGAGATCATGGTCCTACATTGTAGAAACACCAAACTCTGAGAATGGAGCATGTTATCCAGGAGATCTCATCGACTATGAGGAACTGAGGGAGCAATTGAGCTCAGTATCATCATTAGAAAGATTCGAAATATTTCCCAAGGAAAGTTCATGGCCCAACCACACATTCAACGGAGTAACAGTATCATGCTCCCATAGGGGAAAAAGCAGTTTTTACAGAAATTTGCTATGGCTGACGAAGAAGGGGGATTCATACCCAAAGCTGACCAATTCCTATGTGAACAATAAAGGGAAAGAAGTCCTTGTACTATGGGGTGTTCATCACCCGTCTAGCAGTGATGAGCAACAGAGTCTCTATAGTAATGGAAATGCTTATGTCTCTGTAGCGTCTTCAAATTATAACAGGAGATTCACCCCGGAAATAGCTGCAAGGCCCAAAGTAAGAGATCAACATGGGAGGATGAACTATTACTGGACCTTGCTAGAACCCGGAGACACAATAATATTTGAGGCAACTGGTAATCTAATAGCACCATGGTATGCTTTCGCACTGAGTAGAGGGTTTGAGTCCGGCATCATCACCTCAAACGCGTCAATGCATGAGTGTAACACGAAGTGTCAAACACCCCAGGGAGCTATAAACAGCAATCTCCCTTTCCAGAATATACACCCAGTCACAATAGGAGAGTGCCCAAAATATGTCAGGAGTACCAAATTGAGGATGGTTACAGGACTAAGAAACATCCCATCCATTCAATACAGAGGTCTATTTGGAGCCATTGCTGGTTTTATTGAGGGGGGATGGACTGGAATGATAGATGGATGGTATGGTTATCATCATCAGAATGAACAGGGATCAGGCTATGCAGCGGATCAAAAAAGCACACAAAATGCCATTAACGGGATTACAAACAAGGTGAACTCTGTTATCGAGAAAATGAACACTCAATTCACAGCTGTGGGTAAAGAATTCAACAACTTAGAAAAAAGGATGGAAAATTTAAATAAAAAAGTTGATGATGGGTTTCTGGACATTTGGACATATAATGCAGAATTGTTAGTTCTACTGGAAAATGAAAGGACTTTGGATTTCCATGACTTAAATGTGAAGAATCTGTACGAGAAAGTAAAAAGCCAATTAAAGAATAATGCCAAAGAAATCGGAAATGGGTGTTTTGAGTTCTACCACAAGTGTGACAATGAATGCATGGAAAGTGTAAGAAATGGGACTTATGATTATCCAAAATATTCAGAAGAATCAAAGTTGAACAGGGAAAAGATAGATGGAGTGAAATTGGAATCAATGGGGGTGTATCAGATTCTGGCGATCTACTCAACTGTCGCCAGTTCACTGGTGCTTTTGGTCTCCCTGGGGGCAATCAGTTTCTGGATGTGTTCTAATGGGTCTTTGCAGTGCAGAATATGCATCTGA

>CpG-H

GAATTGGCGGAAGGCCGTCAAGGCCACGTGTCTTGTCCGCTTAATTAACACATTCGGTTAACCTGCTCGAAGACAGCCACAACGGGAAACTATGTAAATTAAAAGGAATAGCGCCGCTACAATTGGGGAAATGTAACATCACCGGATGGCTCTTGGGAAATCCCGAATGCGACTCGCTGCTTCCGGCGCGATCGTGGTCGTACATCGTAGAAACGCCGAACTCGGAGAACGGCGCGTGTTATCCGGGCGATCTCATCGACTACGAGGAACTGCGCGAGCAATTGAGCTCCGTATCGTCGTTAGAACGATTCGAAATATTTCCGAAGGAAAGTTCGTGGCCGAACCACACGTTCAACGGCGTAACGGTATCGTGCTCGCATAGGGGAAAAAGCAGTTTTTACCGAAATTTGCTATGGCTGACGAAGAAGGGCGATTCGTACCCGAAGCTGACGAATTCGTACGTGAACAATAAAGGGAAAGAAGTCCTCGTACTATGGGGCGTTCATCACCCGTCTAGCAGCGACGAGCAACAGAGTCTCTATAGTAACGGAAACGCGTACGTCTCGGTAGCGTCGTCGAATTATAACCGGCGATTCACGCCGGAAATAGCCGCGCGGCCGAAAGTACGCGATCAACACGGGCGGATGAACTATTACTGGACGTTGCTAGAACCCGGCGACACGATAATATTCGAGGCGACGGGTAATCTAATAGCGCCGTGGTACGCGTTCGCGCTGAGTAGAGGGTTCGAGTCCGGCATCATCACGTCGAACGCGTCGATGCACGAGTGTAACACGAAGTGTCAAACGCCGCAGGGCGCTATAAACAGCAATCTCCCGTTCCAGAATATACACCCCGTCACGATAGGCGAGTGCCCGAAATACGTCCGGAGTACGAAATTGCGGATGGTTACGGGACTACGAAACATCCCGTCGATTCAATACCGCGGTCTATTCGGCGCGATCGCCGGTTTTATCGAGGGCGGATGGACGGGAATGATAGACGGATGGTACGGTTATCATCATCAGAACGAACAGGGATCCGGCTACGCGGCGGATCAAAAAAGCACGCAAAACGCGATTAACGGGATTACGAACAAGGTGAACTCCGTTATCGAGAAAATGAACACGCAATTCACGGCCGTCGGTAAAGAATTCAACAACTTAGAAAAACGGATGGAAAATTTAAATAAAAAAGTCGACGACGGGTTTCTCGACATTTGGACGTATAACGCGGAATTGTTAGTTCTACTCGAAAACGAACGGACGTTGGATTTCCACGACTTAAACGTGAAGAATCTGTACGAGAAAGTAAAAAGCCAATTAAAGAATAACGCGAAAGAAATCGGAAACGGGTGTTTCGAGTTCTACCACAAGTGCGACAACGAATGCATGGAAAGCGTACGAAACGGGACGTACGATTATCCGAAATATTCGGAAGAATCGAAGTTGAACCGCGAAAAGATAGACGGCGTGAAATTGGAATCGATGGGCGTGTAGGCGCGCCGGAGCACAAGACTGGCCTCATGGGCCTTCCGCTCACTGCC

>UpA-H

CACTATAGGGCGAATTGAAGGAAGGCCGTCAAGGCCGCATGGTACCCACATTCTGTTAACCTACTCGAAGATAGCCATAACGGTAAACTATGTAAATTAAAAGGTATAGCCCCACTACAATTAGGTAAATGTAATATTACCGGATGGCTATTAGGTAATCCAGAATGCGACTCACTACTACCAGCGAGATCATGGTCCTATATAGTAGAAACACCTAACTCTGAGAATGGAGCATGTTATCCAGGAGATCTAATCGACTATGAGGAACTAAGGGAGCAATTAAGCTCAGTATCATCATTAGAAAGATTCGAAATATTTCCTAAGGAAAGTTCATGGCCTAACCATACATTTAACGGAGTAACAGTATCATGCTCCCATAGGGGTAAAAGTAGTTTTTATAGAAATTTACTATGGCTAACGAAGAAGGGGGATTCATACCCTAAGCTTACTAATTCCTATGTAAATAATAAAGGTAAAGAAGTACTAGTACTATGGGGTGTACATCACCCGTCTAGTAGTGATGAGCAACAGAGTCTATATAGTAATGGTAATGCTTATGTATCTGTAGCGTCTTCTAATTATAATAGGAGATTTACCCCGGAAATAGCTGCTAGGCCTAAAGTAAGAGATCAACATGGTAGGATGAACTATTACTGGACCTTACTAGAACCCGGAGATACTATAATATTTGAGGCTACTGGTAATCTAATAGCACCATGGTATGCTTTCGCACTAAGTAGAGGGTTTGAGTCCGGTATTATAACCTCTAACGCGTCTATGCATGAGTGTAATACGAAGTGTCAAACACCCCAGGGAGCTATAAATAGTAATCTACCTTTCCAGAATATACACCCAGTTACTATAGGAGAGTGCCCTAAATATGTAAGGAGTACTAAATTAAGGATGGTTACAGGACTAAGAAATATACCATCTATACAATATAGAGGTCTATTTGGAGCTATAGCTGGTTTTATAGAGGGGGGATGGACTGGTATGATAGATGGATGGTATGGTTATCATCATCAGAATGAACAGGGATCAGGCTATGCAGCGGATCAAAAAAGTACACAAAATGCTATTAACGGTATTACTAATAAGGTTAACTCTGTTATCGAGAAAATGAATACTCAATTTACAGCTGTAGGTAAAGAATTTAATAACTTAGAAAAAAGGATGGAAAATTTAAATAAAAAAGTAGATGATGGGTTTCTAGATATATGGACATATAATGCAGAATTATTAGTACTACTAGAAAATGAAAGGACTTTAGATTTCCATGACTTAAATGTTAAGAATCTATACGAGAAAGTAAAAAGCCAATTAAAGAATAATGCTAAAGAAATCGGTAATGGGTGTTTTGAGTTCTACCATAAGTGTGATAATGAATGTATGGAAAGTGTAAGAAATGGTACTTATGATTATCCTAAATATTCAGAAGAATCTAAGTTAAATAGGGAAAAGATAGATGGAGTTAAATTAGAATCTATGGGGGTGTAGAGCTCCTGGGCCTCATGGGCCTTCCTTTCACTGCCCGCTTTCCAG
