# Supplementary material for: Comparison of CpG- and UpA-mediated restriction of RNA virus replication in mammalian and avian cells and investigation of potential ZAP-mediated shaping of host transcriptome compositions
Source: RNA. 2022 Aug;28(8):1089–109. doi: 10.1261/rna.079102.122 (PMC9297844; doi:10.1261/rna.079102.122)
Supplement: Supplemental Material [file supp_079102.122_Supplemental_Material_.zip › Supplemental_Table_S10.docx]

TABLE S10

AVIAN INTERFERON α, β and γ GENES

Gene Species_Accession number

IFN-α Anas_platyrhynchos_platyrhynchos.CAU_duck1.0.dna_sm.toplevel_Z:1801986-1802456_(reversed)

IFN-α Chaetura_pelagica_C11431834:321-795_(reversed)

IFN-α Chaetura_pelagica_scaffold548:10270-10744_(reversed)

IFN-α Corvus_brachyrhynchos_scaffold139:1684992-1685502_(reversed)

IFN-α Corvus_brachyrhynchos_scaffold139:1694658-1695201_(reversed)

IFN-α Cuculus_canorus_scaffold818:510922-511349

IFN-α Gallus_gallus.GRCg6a.dna_sm.toplevel_Z:7377585-7378166

IFN-α Gallus_gallus.GRCg6a.dna_sm.toplevel_Z:7381557-7382138

IFN-α Gallus_gallus.GRCg6a.dna_sm.toplevel_Z:7385529-7386110

IFN-α Gallus_gallus.GRCg6a.dna_sm.toplevel_Z:7387338-7387919

IFN-α Gallus_gallus.GRCg6a.dna_sm.toplevel_Z:7391315-7391896

IFN-α Gallus_gallus.GRCg6a.dna_sm.toplevel_Z:7395287-7395868

IFN-α Gallus_gallus.GRCg6a.dna_sm.toplevel_Z:7399256-7399837

IFN-α Gallus_gallus.GRCg6a.dna_sm.toplevel_Z:7401325-7401906

IFN-α Gallus_gallus.GRCg6a.dna_sm.toplevel_Z:7405294-7405874

IFN-α Gallus_gallus.GRCg6a.dna_sm.toplevel_Z:7410336-7410917

IFN-α Gallus_gallus.GRCg6a.dna_sm.toplevel_Z:7414305-7414886

IFN-α Gallus_gallus.GRCg6a.dna_sm.toplevel_Z:7422010-7422591

IFN-α Haliaeetus_leucocephalus_Scaffold863:2727-3282_(reversed)

IFN-α HM007810.1_Casuarius_casuarius_interferon_alpha_gene_cds

IFN-α Manacus_vitellinus_scaffold662:4605-5004_(reversed)

IFN-α Mesitornis_unicolor_scaffold11076:185-631

IFN-α MG833835.1_Columba_livia_interferon_alpha_precursor_(IFNA)_gene_complete_cds

IFN-α Nestor_notabilis_C14677681:15-421

IFN-α Nipponia_nippon_Scaffold2307:3076-3619

IFN-α Pelecanus_crispus_C7171222:243-813_(reversed)

IFN-α Phalacrocorax_carbo_scaffold40798:1565-2124_(reversed)

IFN-α Picoides_pubescens_scaffold219:1537914-1538484

IFN-α Struthio_camelus.20130116.OM_superscaffold26:10398481-10399040

IFN-α Taeniopygia_guttata.taeGut3.2.4.dna_sm.toplevel_Z:38050937-38051444

IFN-α U28140.1_Meleagris_gallopavo_interferon_cds

IFN-α XM005060418.2_Ficedula_albicollis_interferon-like_transcript_variant_X2

IFN-α XM005236164.3_Falco_peregrinus_interferon-like

IFN-α XM009687092.1_Struthio_camelus_australis_interferon-like

IFN-α XM009886412.1_Charadrius_vociferus_interferon-like

IFN-α XM009974126.1_Tyto_alba_interferon-like

IFN-α XM010124638.1_Chlamydotis_macqueenii_interferon-like

IFN-α XM010561124.1_Haliaeetus_leucocephalus_interferon-like

IFN-α XM010726527.2_Meleagris_gallopavo_interferon-like

IFN-α XM010727621.2_Meleagris_gallopavo_interferon

IFN-α XM010728566.1_Meleagris_gallopavo_interferon-like

IFN-α XM014259137.1_Pseudopodoces_humilis_interferon-like

IFN-α XM014259141.1_Pseudopodoces_humilis_interferon-like

IFN-α XM014268937.2_Zonotrichia_albicollis_interferon

IFN-α XM014308634.1_Geospiza_fortis_interferon-like

IFN-α XM017746589.1_Corvus_brachyrhynchos_interferon-like

IFN-α XM023944242.1_Cyanistes_caeruleus_interferon-like

IFN-α XM023947935.1_Cyanistes_caeruleus_interferon-like

IFN-α XM026865334.1_Athene_cunicularia_interferon-like

IFN-α XM027670382.1_Neopelma_chrysocephalum_interferon-like

IFN-α XM027707865.1_Neopelma_chrysocephalum_interferon-like

IFN-α XM027731094.1_Pipra_filicauda_interferon-like

IFN-α XM027731096.1_Pipra_filicauda_interferon-like

IFN-α XM027731097.1_Pipra_filicauda_interferon-like

IFN-α XM027731098.1_Pipra_filicauda_interferon-like

IFN-α XM030005126.1_Aquila_chrysaetos_chrysaetos_interferon-like

IFN-α XM030005127.1_Aquila_chrysaetos_chrysaetos_interferon-like_transcript_variant_X1

IFN-α XM030005128.1_Aquila_chrysaetos_chrysaetos_interferon-like_transcript_variant_X2

IFN-α XM030005129.1_Aquila_chrysaetos_chrysaetos_interferon-like

IFN-α XM030258193.1_Taeniopygia_guttata_interferon-like

IFN-α XM030258194.1_Taeniopygia_guttata_interferon-like

IFN-β ENSCMMP00000018868_Muscovy_Duck_domestic_type_

IFN-β ENSAZOP00000004406_Eastern_spot-billed_duck_Anas_zonorhyncha

IFN-β ENSAPLP00020026911_Mallard_Anas_platyrhynchos

IFN-β ENSAPLP00000021652_Mallard_Anas_platyrhynchos

IFN-β ENSACDP00005025916_Swan_goose_Anser_cygnoides Galliform

IFN-β ENSABRP00000018960_Pink-footed_goose_Anser_brachyrhynchus

IFN-β ENSAZOP00000004471_Eastern_spot-billed_duck_Anas_zonorhyncha

IFN-β ENSCPGP00000015638_Spoon-billed_sandpiper_Calidris_pygmaea

IFN-β ENSANIP00000008294_Eurasian_sparrowhawk_Accipiter_nisus

IFN-β ENSACCP00020023191_Golden_eagle_Aquila_chrysaetos_chrysaetos

IFN-β ENSACCP00020023182_Golden_eagle_Aquila_chrysaetos_chrysaetos

IFN-β ENSACCP00020023162_Golden_eagle_Aquila_chrysaetos_chrysaetos

IFN-β ENSCCEP00000000423_Blue_tit_Cyanistes_caeruleus

IFN-β ENSCPVP00000018040_Small_tree_finch_Camarhynchus_parvulus

IFN-β ENSNPEP00000006195_Chilean_tinamou_Nothoprocta_perdicaria

IFN-β ENSARWP00000011501_Okarito_brown_kiwi_Apteryx_rowi

IFN-β ENSAOWP00000014397_Little_spotted_kiwi_Apteryx_owenii

IFN-β ENSAHAP00000024921_Great_spotted_kiwi_Apteryx_haastii

IFN-β ENSNMEP00000017399_Helmeted_guineafowl_Numida_meleagris

IFN-β ENSCJPP00005005906_Japanese_quail_Coturnix_japonica

IFN-β ENSNMEP00000002413_Helmeted_guineafowl_Numida_meleagris

IFN-β ENSMGAP00000016316_Turkey_Meleagris_gallopavo

IFN-β ENSPSTP00000005634_Indian_peafowl_Pavo_cristatus

IFN-γ Acanthisitta_chloris.cds_Ach_R008406:1-490

IFN-γ Anas_platyrhynchos_platyrhynchos.CAU_duck1.0.cds.all.fa_ENSAPLT00000013404.2:1-495

IFN-γ Antrostomus_carolinensis.cds_Cca_R000530:1-494

IFN-γ Apaloderma_vittatum.cds_Avi_R011663:1-487

IFN-γ Aptenodytes_forsteri.cds_Afo_R009741:1-495

IFN-γ Balearica_regulorum.cds_Bre_R002930:1-495

IFN-γ Buceros_rhinoceros.cds_Brh_R006464:1-495

IFN-γ Calypte_anna.cds_Aan_R002602:1-490

IFN-γ Cariama_cristata.cds_Ccr_R002113:1-495

IFN-γ Cathartes_aura.cds_Cau_R002264:1-495

IFN-γ Chaetura_pelagica.cds_Cpe_R007590:1-495

IFN-γ Charadrius_vociferus.cds_Cvo_R001510:1-490

IFN-γ Chlamydotis_macqueenii.cds_Cun_R005582:1-495

IFN-γ Colius_striatus.cds_Cst_R001217:1-495

IFN-γ Columba_livia.cds_Cli_R015848:1-495

IFN-γ Corvus_brachyrhynchos.cds_Cbr_R008853:1-495

IFN-γ Cuculus_canorus.cds_Cca_R009561:1-495

IFN-γ Egretta_garzetta.cds_Ega_R014273:1-468

IFN-γ Eurypyga_helias.cds_Ehe_R003070:1-495

IFN-γ Fulmarus_glacialis.cds_Fgl_R012296:1-495

IFN-γ Gallus_gallus.GRCg6a.cds.all.fa_ENSGALT00000016105.4:1-495

IFN-γ Gavia_stellata.cds_Gst_R010542:1-495

IFN-γ Geospiza_fortis.gene.cds_Gfo_R004015:1-495

IFN-γ Leptosomus_discolor.cds_Ldi_R014075:1-495

IFN-γ Manacus_vitellinus.cds_Mvi_R002783:1-495

IFN-γ Meleagris_gallopavo.Turkey_2.01.cds.all.fa_ENSMGAT00000011672.2:1-495

IFN-γ Merops_nubicus.cds_Mnu_R007969:1-492

IFN-γ Mesitornis_unicolor.cds_Mun_R011541:1-485

IFN-γ Nestor_notabilis.cds_Nno_R012732:1-495

IFN-γ Nipponia_nippon.cds_Nni_R011197:1-495

IFN-γ Opisthocomus_hoazin.cds_Oho_R004413:1-495

IFN-γ Pelecanus_crispus.cds_Pcr_R003511:1-495

IFN-γ Phaethon_lepturus.cds_Ple_R011034:1-492

IFN-γ Phalacrocorax_carbo.cds_Pca_R005790:1-495

IFN-γ Phoenicopterus_ruber.cds_Pru_R010707:1-468

IFN-γ Picoides_pubescens.cds_Ppu_R010430:1-490

IFN-γ Podiceps_cristatus.cds_Pcr_R011001:1-477

IFN-γ Pterocles_gutturalis.cds_Pgu_R013465:1-495

IFN-γ Pygoscelis_adeliae.cds_Pad_R001028:1-495

IFN-γ Struthio_camelus.OM.gene.20130116.cds_Sca_R006319:1-494

IFN-γ Taeniopygia_guttata.taeGut3.2.4.cds.all.fa_ENSTGUT00000006979.1:1-498

IFN-γ Tauraco_erythrolophus.cds_Ter_R001782:1-495

IFN-γ Tyto_alba.cds_Tal_R012950:1-495
