# Supplementary material for: Comparison of CpG- and UpA-mediated restriction of RNA virus replication in mammalian and avian cells and investigation of potential ZAP-mediated shaping of host transcriptome compositions
Source: RNA. 2022 Aug;28(8):1089–109. doi: 10.1261/rna.079102.122 (PMC9297844; doi:10.1261/rna.079102.122)
Supplement: Supplemental Material [file supp_079102.122_Supplemental_Material_.zip › Supplemental_Table_S11.docx]

TABLE S11

TOTAL NUMBER OF VIRUS GENOMES AND COMPONENT GENES ANALYSED FOR COMPOSITION

|  | **Genomes** | | **Genes** | |
| --- | --- | --- | --- | --- |
| **Family** | **Avian** | **Mamm.** | **Avian** | **Mamm.** |
| *Arenaviridae* | 0 | 74 | 0 | 144 |
| *Arteriviridae* | 0 | 20 | 0 | 230 |
| *Astroviridae* | 3 | 8 | 8 | 23 |
| *Bornaviridae* | 4 | 2 | 24 | 11 |
| *Caliciviridae* | 2 | 9 | 4 | 23 |
| *Coronaviridae* | 7 | 31 | 63 | 264 |
| *Filoviridae* | 0 | 10 | 0 | 82 |
| *Flaviviridae* | 0 | 57 | 0 | 57 |
| *Hantaviridae* | 0 | 82 | 0 | 82 |
| *Hepeviridae* | 4 | 13 | 12 | 52 |
| *Kolmioviridae* | 0 | 8 | 0 | 9 |
| *Matonaviridae* | 0 | 1 | 0 | 2 |
| *Nairoviridae* | 0 | 3 | 0 | 3 |
| *Orthomyxoviridae* | 0 | 3 | 0 | 27 |
| *Paramyxoviridae* | 20 | 46 | 129 | 357 |
| *Peribunyaviridae* | 0 | 9 | 0 | 10 |
| *Picornaviridae* | 21 | 77 | 21 | 80 |
| *Pneumoviridae* | 4 | 9 | 36 | 92 |
| *Retroviridae* | 8 | 43 | 15 | 213 |
| *Rhabdoviridae* | 1 | 27 | 7 | 149 |
| *Tobaniviridae* | 0 | 3 | 0 | 19 |
|  |  |  |  |  |
| *Total* | 74 | 535 | 319 | 1911 |
