# Supplementary material for: Comparison of CpG- and UpA-mediated restriction of RNA virus replication in mammalian and avian cells and investigation of potential ZAP-mediated shaping of host transcriptome compositions
Source: RNA. 2022 Aug;28(8):1089–109. doi: 10.1261/rna.079102.122 (PMC9297844; doi:10.1261/rna.079102.122)
Supplement: Supplemental Material [file supp_079102.122_Supplemental_Material_.zip › Supplemental_Table_S12.docx]

TABLE S12

ACCESSION NUMBERS OF RNA VIRUS SEQUENCES FROM ICTV VMR

Mammalian viruses:

U04608, LN713680, JF828358, FJ217161, AF522874, AY729654, FJ217162, AF086833, DQ217792, DQ447649, MG599980, MG599981, JQ001776, HQ660129, AF017149, KF278639, AF212302, DQ100461, AY900001, MG573140, MG573141, MG516455, JN689227, AF014953, AJ608288, JQ411014, AB016162, KC802221, X98291, AJ849636, AY286409, MF943130, FJ362497, AF079780, AF178654, KT215610, AF457102, EU326526, AB195968, JX857409, JQ697837, HQ660095, X57559, AB543336, AF052755, EF095490, AB040874, BK005918, X64275, JX051319, JX051320, JX112711, KF774436, KP271124, AF298895, GU128080, GU128081, GU128082, AF371337, FJ168779, AY525843, FJ168778, AF295543, AF092942, AF013254, AY729016, AY743909, KM085029, KC676792, KM204992, KC984953, KJ179955, KM205026, AB609604, KM204998, MG021441, EF614259, AF081020, JF311903, EU293119, EF157976, EF157977, KU244266, JX193798, EF614260, EF614261, EU293108, KY006983, M13215, GU170201, EF614258, KP324828, AY840978, JX569193, AY216502, AY012687, AY358022, AY358023, AY924390, AY924391, AY358026, U41071, AF427517, K02734, EU260464, EU260463, AY216519, AF512832, EU627611, AF512831, KJ855307, KJ855308, AY358024, AY129247, DQ328878, DQ328877, U73034, J04324, EU627612, AF512830, KC669693, KC669698, FJ952385, FJ952384, AB586645, AB586644, AB972431, AB972430, AB693151, AB693150, AY847351, AY847350, AY358021, AY129248, KP867641, KM272987, GU078661, GU078660, DQ328876, AY342390, AY772169, AY772170, EU914104, EU914103, KP867642, KM272988, AY216514, U34248, EU627613, AF485261, AY494081, AF485262, KM020190, KM020191, AY216517, AF485256, AB972429, AB972428, KP050226, KP050227, J04340, M20304, AY924393, AF485263, KJ909795, KJ909794, AY924395, AF228063, KM102249, KM102248, KM102247, KR072623, KR072622, KR072621, KU950715, KU950714, KU950713, AF291704, AF291703, AF291702, KC880347, KC880344, KC880341, GQ244526, L36930, L36929, KC631784, KC631783, KC631782, KX551962, KX551961, KX551960, GQ200821, DQ284451, DQ285566, EF543525, EF543526, EF543524, AJ410617, AJ410616, AJ410615, KT899703, KT899702, KT899701, X55901, M14627, M14626, HQ834697, HQ834696, HQ834695, KJ857320, KJ857337, KJ857341, KJ857321, KJ857338, KJ857342, HQ404253, HM756287, HM756286, EU788002, AY363179, AY267347, AB620102, AB620101, AB620100, EF646763, X55129, HM015221, HM015219, HM015223, JQ082302, JQ082301, JQ082300, X56492, S47716, AY273791, L37902, L37903, L37904, KC490918, KC490919, KC490922, KU934008, AJ005637, KJ420567, KJ420541, KJ420559, DQ825770, DQ825771, AY526097, KR537444, KR537445, KR537446, KP792660, KP792659, KP792658, JQ675603, JQ675602, JQ675601, KM225260, KM225261, KM225262, M14880, AF101982, AF102017, K00423, K01395, J02095, J02094, J02096, AB126191, AB126192, AB126193, AB126194, AB126195, AB126196, AB283001, JQ922305, JQ922306, JQ922307, JQ922308, JQ922309, JQ922310, JQ922311, MF324848, X53459, KP026921, AF180391, KR139839, KM677927, KR862307, KT166441, KC787658, KP126831, JX473849, KT447550, KM110938, KC787630, U87392, KP280006, KU302440, M96262, U15146, JN116253, KF430219, JQ989270, KJ473807, AF304460, KF294380, LC119077, HM245925, EU420138, EU420139, KJ473806, KJ473809, AF353511, DQ648858, EF203064, AY567487, KY073745, AJ271965, AY585228, KM349742, AY597011, AY700211, KF636752, KC545383, JX869059, EF065509, EF065505, KU762338, EF065513, AY274119, JQ065043, EU111742, KM589359, AY427798, JQ860350, MF327529, KP236128, EU236594, DQ272578, AY593829, EU142040, KM589358, M81861, X56019, JQ864242, FJ438902, FJ438907, FJ438908, FJ555055, JN867758, AB937989, KX644937, JN819202, MF188967, AY421760, M88483, V01149, AY426531, D00214, DQ092770, AF363453, AF326759, KP345887, AF326766, KX156158, KU587555, FJ445111, DQ473485, EF077279, X96871, M14707, KR703607, KT452742, KT452637, KT452735, KT452685, KT452730, KT452691, KT452658, JQ941880, AB040749, AB084788, EU787450, LC055961, KX644936, KY670597, KM396707, KM396708, JQ814851, KP054273, KP100644, KY512802, JF973687, S45208, AF327920, HF677705, KF006989, JQ316470, KY432926, KY855432, MF175072, JF973686, KX783423, KX783424, KF387721, GQ179640, AF406813, AY064708, DQ641257, KJ641698, AJ011380, LC113907, Z25771, KM017741, KF787112, FJ222451, GQ415660, FJ973620, AY179509, Y15937, Z69620, M67473, AY082891, M87661, EU391643, AY694184, FJ355928, L40021, U76874, KP325401, U22304, AF009606, M62321, D90208, D00944, D10988, D17763, D63821, GU814265, Y13184, Y12083, D63822, EF108306, KC551800, KC815310, KC411784, KJ950938, KX905133, KJ950939, KC411806, KC411777, KC796074, KC796077, KC796078, KP641127, U22303, U94421, GU566734, U44402, U63715, AB003289, U45966, AY196904, AB021287, AY949771, AB003292, AF070476, KC145265, KC410872, KC796080, KC796076, KT439329, KC796088, KC815311, KU351669, M31182, U18059, X87939, AF037405, AY781152, EF100713, AF144617, FJ040215, JX428945, KJ950914, KR011347, M73218, L08816, AF082843, EF077630, AB573435, AB602441, KJ496143, KX387865, GU345042, KM516906, LC057247, JN998606, JQ001749, M15240, M21012, X60193, L22063, AF018077, AJ584848, AJ584847, AJ584844, AJ584849, M80216, AF033815, AF033807, M23385, AF033818, AF074966, M10060, AF391797, AF052723, K02712, M26927, AF151794, AF033813, Z11128, V01201, M32690, M33677, AF033820, M25381, AF033819, M30502, U21603, U03982, M58410, L06906, U94514, AF201902, Y08851, KM233624, AJ544579, JQ867463, LC094267, KX087159, M74895, JQ867466, AB923518, MF280817, EU010385, GU356394, U04327, HM245790, GU356395, KP143760

Avian viruses:

KC464471, GU249595, KR612223, KF578398, EU338414, GU206351, EF569970, FJ231524, FJ215863, HM147142, JQ886184, KX258200, KX932454, MF033136, AY562991, EU910942, KC333050, LC041132, KY511044, KY452442, KY452443, KY452444, EU782025, EU877976, AY640317, AB548428, AY590688, HG934339, FJ952155, JQ065048, FJ376619, FJ376622, JQ065044, JQ065047, JQ065049, M95169, KJ000696, AY563023, DQ226541, KC614703, KF979333, KF979334, JQ691613, KP230449, KC935379, KC663628, KC876003, KF961186, KY488458, MF405436, KM203656, GU182408, GU182406, MF977321, KU977108, KF741227, AJ225173, Y15936, AB033998, AF206663, HQ010042, JQ347522, AY535004, EF206691, GU954430, JN597006, M14008, M37980, AF033809, Y00302, AF033808, M10455, L21974, AY842951
