# Supplementary material for: Comparison of CpG- and UpA-mediated restriction of RNA virus replication in mammalian and avian cells and investigation of potential ZAP-mediated shaping of host transcriptome compositions
Source: RNA. 2022 Aug;28(8):1089–109. doi: 10.1261/rna.079102.122 (PMC9297844; doi:10.1261/rna.079102.122)
Supplement: Supplemental Material [file supp_079102.122_Supplemental_Material_.zip › Supplemental_Table_S13.docx]

TABLE S13

COMPARISON OF CpG AND UpA REPRESENTATION IN AVIAN AND MAMMALIAN RNA VIRUSES

A) Comparison of compositions of RNA viruses infecting avian and mammalian hosts

|  | **Species totals^1^** | | **G+C composition** | | | | **CpG O/E** | | | | **UpA O/E** | | | |
| --- | --- | --- | --- | --- | --- | --- | --- | --- | --- | --- | --- | --- | --- | --- |
| **Family** | **Mammal** | **Avian** | **Mamm** | **Avian** | **Diff^.^** | ***P^3^*** | **Mamm** | **Avian** | **Diff.** | ***p*** | **Mamm** | **Avian** | **Diff.** | ***p*** |
| *Astroviridae* | 23 | 8 | 46.2% | 44.5% | -3.8% | 0.34 | 0.489 | 0.425 | -14% | 0.34 | 0.710 | 0.656 | -8.0% | 0.05 |
| *Bornaviridae* | 8 | 16 | 48.6% | 43.6% | -11% | 0.01 | 0.632 | 0.614 | -2.9% | 0.35 | 0.747 | 0.740 | -0.9% | 0.49 |
| *Caliciviridae* | 19 | 4 | 49.7% | 51.4% | 3.3% | 0.37 | 0.520 | 0.488 | -6.4% | 0.67 | 0.577 | 0.658 | 13.0% | 0.08 |
| *Coronaviridae* | 190 | 30 | 41.2% | 41.3% | 0.5% | 0.88 | 0.551 | 0.441 | -22% | **7E-07** | 0.852 | 0.871 | 2.2% | 0.20 |
| *Hepeviridae* | 37 | 8 | 56.3% | 55.7% | -1.0% | 0.74 | 0.809 | 0.796 | -1.7% | 0.99 | 0.902 | 0.927 | 2.7% | 0.41 |
| *Paramyxoviridae* | 346 | 128 | 43.5% | 45.9% | 5.2% | **4E-06** | 0.439 | 0.489 | 10.6% | **6E-07** | 0.720 | 0.733 | 1.8% | 0.48 |
| *Picornaviridae* | 80 | 21 | 45.0% | 46.8% | 4.0% | 0.21 | 0.431 | 0.451 | 4.4% | 0.71 | 0.626 | 0.645 | 3.0% | 0.31 |
| *Pneumoviridae* | 69 | 32 | 39.1% | 45.1% | 14.3% | **4E-08** | 0.235 | 0.298 | 23.4% | **4E-03** | 0.771 | 0.739 | -4.2% | 0.23 |
| *Retroviridae* | 189 | 14 | 44.7% | 55.2% | 21.1% | **1E-06** | 0.376 | 0.646 | 52.8% | **5E-07** | 0.829 | 0.749 | -10.2% | 0.06 |
| *Rhabdoviridae* | 139 | 5 | 43.7% | 44.6% | 2.0% | 0.79 | 0.429 | 0.492 | 13.8% | 0.18 | 0.607 | 0.566 | -7.0% | 0.66 |
|  |  |  |  |  |  |  |  |  |  |  |  |  |  |  |
| All | 1665 | 266 | 46.00% | 44.47% | -3.4% | **1E-05** | 0.464 | 0.480 | 3.6% | **0.04** | 0.723 | 0.743 | 2.6% | 0.08 |

B) Comparison of compositions of different segments of IAV strains infecting ducks and chickens^4^

|  | **G+C composition** | | | | **CpG O/E** | | | | **UpA O/E** | | | |
| --- | --- | --- | --- | --- | --- | --- | --- | --- | --- | --- | --- | --- |
| **Segment** | **Duck** | **Chicken** | **Diff.** | ***P*^5^** | **Duck** | **Chicken** | **Diff.** | ***p*^5^** | **Duck** | **Chicken** | **Diff.** | ***p*^5^** |
| 1 | 44.56% | 44.62% | -0.06% | 0.111 | 0.4633 | 0.4677 | -0.44% | 0.072 | 0.6058 | 0.618 | -1.22% | 3.8E-06 |
| 2 | 43.37% | 43.24% | 0.13% | 5.5E-04 | 0.4306 | 0.4546 | -2.40% | 1.6E-13 | 0.5723 | 0.5936 | -2.13% | <1E-30 |
| 3 | 44.14% | 43.97% | 0.17% | 1.5E-07 | 0.5157 | 0.5164 | -0.07% | 0.46 | 0.4818 | 0.4782 | 0.36% | 0.008 |
| 4 | 41.88% | 41.62% | 0.26% | 1.0E-03 | 0.387 | 0.3821 | 0.49% | 0.10 | 0.6472 | 0.681 | -3.38% | <1E-30 |
| 5 | 47.42% | 47.40% | 0.02% | 0.74 | 0.4644 | 0.4726 | -0.82% | 1.9E-04 | 0.4107 | 0.4542 | -4.35% | <1E-30 |
| 6 | 43.24% | 43.29% | -0.05% | 0.15 | 0.3485 | 0.3364 | 1.21% | 2.2E-04 | 0.7131 | 0.6868 | 2.63% | 1.3E-09 |
| 7 | 50.62% | 49.79% | 0.83% | <1E-30 | 0.4884 | 0.4973 | -0.89% | 4.9E-05 | 0.5909 | 0.6514 | -6.05% | <1E-30 |
| 8 | 46.09% | 45.89% | 0.20% | 3.5E-04 | 0.4922 | 0.5133 | -2.11% | 5.3E-06 | 0.4446 | 0.4613 | -1.67% | 1.9E-06 |
|  |  |  |  |  |  |  |  |  |  |  |  |  |
| All | 45.16% | 44.98% | 0.18% | 0.07 | 0.4487 | 0.4551 | -0.64% | 0.042 | 0.5583 | 0.5781 | -1.98% | 8.9E-16 |

C) Comparison of compositions of different segments of IAV strains infecting birds and mammals^4^

|  | **G+C composition** | | | | **CpG O/E** | | | | **UpA O/E** | | | |
| --- | --- | --- | --- | --- | --- | --- | --- | --- | --- | --- | --- | --- |
| **Segment** | **Avian** | **Mamm.** | **Diff.** | ***p*^5^** | **Avian** | **Mamm.** | **Diff.** | ***p*^5^** | **Avian** | **Mamm.** | **Diff.** | ***p*^5^** |
| 1 | 44.58% | 43.79% | 0.79% | <1E-30 | 0.4651 | 0.4315 | 3.36% | <1E-30 | 0.6108 | 0.606 | 0.48% | 0.05 |
| 2 | 43.32% | 42.35% | 0.97% | <1E-30 | 0.4405 | 0.3957 | 4.48% | <1E-30 | 0.5811 | 0.6071 | -2.60% | <1E-30 |
| 3 | 44.07% | 43.12% | 0.95% | <1E-30 | 0.516 | 0.456 | 6.00% | <1E-30 | 0.4803 | 0.5055 | -2.52% | <1E-30 |
| 4 | 41.77% | 41.44% | 0.33% | 2.0E-06 | 0.385 | 0.3703 | 1.47% | <1E-30 | 0.6612 | 0.6462 | 1.50% | 7.0E-07 |
| 5 | 47.41% | 46.14% | 1.27% | <1E-30 | 0.4678 | 0.482 | -1.42% | 1.8E-13 | 0.4287 | 0.4792 | -5.05% | <1E-30 |
| 6 | 43.26% | 42.41% | 0.85% | <1E-30 | 0.3435 | 0.3829 | -3.94% | <1E-30 | 0.7022 | 0.6973 | 0.49% | 0.98 |
| 7 | 50.27% | 48.76% | 1.51% | <1E-30 | 0.4921 | 0.4784 | 1.37% | 7.8E-05 | 0.616 | 0.6304 | -1.44% | 3.5E-05 |
| 8 | 46.01% | 45.61% | 0.40% | 6.6E-13 | 0.5009 | 0.5637 | -6.28% | <1E-30 | 0.4515 | 0.4895 | -3.80% | <1E-30 |
|  |  |  |  |  |  |  |  |  |  |  |  |  |
| All | 45.09% | 44.20% | 0.89% | 0.07 | 0.4514 | 0.4451 | 0.63% | 0.042 | 0.5665 | 0.5827 | -1.62% | 8.9E-16 |

D) Comparison of compositions of different segments of IAV strains infecting birds and mammals excluding H5N1

|  | **G+C composition** | | | | **CpG O/E** | | | | **UpA O/E** | | | |
| --- | --- | --- | --- | --- | --- | --- | --- | --- | --- | --- | --- | --- |
| **Segment** | **Avian** | **Mamm.** | **Diff.** | ***p*** | **Avian** | **Mamm.** | **Diff.** | ***p*** | **Avian** | **Mamm.** | **Diff.** | ***p*** |
| 1 | 44.58% | 43.67% | 0.91% | <1E-30 | 0.4651 | 0.4228 | 4.23% | <1E-30 | 0.6108 | 0.6043 | 0.65% | 0.04 |
| 2 | 43.32% | 42.20% | 1.12% | <1E-30 | 0.4405 | 0.375 | 6.55% | <1E-30 | 0.5811 | 0.614 | -3.29% | <1E-30 |
| 3 | 44.07% | 43.07% | 1.00% | <1E-30 | 0.516 | 0.4449 | 7.11% | <1E-30 | 0.4803 | 0.5066 | -2.63% | <1E-30 |
| 4 | 41.77% | 41.61% | 0.16% | 0.22 | 0.385 | 0.3692 | 1.58% | 0.01 | 0.6612 | 0.6294 | 3.18% | <1E-30 |
| 5 | 47.41% | 45.93% | 1.48% | <1E-30 | 0.4678 | 0.4841 | -1.63% | 8.7E-14 | 0.4287 | 0.4836 | -5.49% | <1E-30 |
| 6 | 43.26% | 42.22% | 1.04% | <1E-30 | 0.3435 | 0.3919 | -4.84% | <1E-30 | 0.7022 | 0.698 | 0.42% | 0.63 |
| 7 | 50.27% | 48.58% | 1.69% | <1E-30 | 0.4921 | 0.4681 | 2.40% | 1.4E-11 | 0.616 | 0.6237 | -0.77% | 0.03 |
| 8 | 46.01% | 45.53% | 0.48% | 1.3E-15 | 0.5009 | 0.5727 | -7.18% | <1E-30 | 0.4515 | 0.4926 | -4.11% | <1E-30 |
|  |  |  |  |  |  |  |  |  |  |  |  |  |
| All | 45.09% | 44.10% | 0.99% | <1E-30 | 0.4514 | 0.4411 | 1.03% | <1E-30 | 0.5665 | 0.5815 | -1.50% | 1.6E-10 |

^1^Total number of genes sequences compared derived from separate species or representative sequences within each family containing both avian and mammalian viruses; data derived from the ICTV virus metadata resource: <https://talk.ictvonline.org/taxonomy/vmr/m/vmr-file-repository/12323> (accession numbers listed in Table S11; Suppl. Data).

^2^Directionality colour code – Blue: higher values in first column; red: lower values in first column.

^3^Cells shaded by probability range.

^4^Coding regions from IAV segments 1-8 from viruses representing a range of serotypes listed in the Influenza Research Database <https://www.fludb.org/brc/influenza_sequence_search_segment_display.spg?method=ShowCleanSearch&decorator=influenza> (accession numbers listed in Table S12; (Suppl. Data)

^5^For analysis of IAV sequences, sequences possessed phylogenetic structure and metrics of composition therefore did not constitute independent observations in the majority of cases. Consequently, *p* values are substantially over-stated, but can be used as an approximate indicator of degrees of their compositional differences.
