# Supplementary material for: Comparison of CpG- and UpA-mediated restriction of RNA virus replication in mammalian and avian cells and investigation of potential ZAP-mediated shaping of host transcriptome compositions
Source: RNA. 2022 Aug;28(8):1089–109. doi: 10.1261/rna.079102.122 (PMC9297844; doi:10.1261/rna.079102.122)
Supplement: Supplemental Material [file supp_079102.122_Supplemental_Material_.zip › Supplemental_Table_S14.docx]

TABLE S14

ACCESSION NUMBERS OF IAV SEQUENCES FROM THE INFLUENZA RESEARCH DATABASES

Duck:

JN716357, JN716341, JN716347, JN716352, JN716326, JN716321, JN716346, JN716331, JN716336, JN716322, JN716348, JN716353, JN716338, JN716323, JN716358, JN716332, JN716337, JN716342, JN716327, JN716351, JN716335, JN716320, JN716325, JN716330, JN716345, JN605389, JN605377, JN716350, JN716355, JN716340, JN605379, JN605383, JN716356, JN605391, JN605387, JN605399, JN605403, JN605375, JN605395, JQ906590, JQ906586, JQ906567, JQ906563, JQ906582, JQ906570, JQ906566, JQ906578, JQ906574, JQ906571, JQ906568, JQ906564, JQ906576, JQ906572, JQ906591, JQ906579, JQ906575, JQ906587, JQ906583, JQ906562, JN716319, JN716344, JN716329, JN716334, JN716349, JN716328, JN716333, JN716354, JN716343, JN716324, JN605382, JN605386, JN605390, JN605378, JN605374, JN605402, JN716339, JN605394, JN605398, JN605381, AB569493, AB569492, AB569495, AB569494, AB569483, AB569480, AB569479, AB569482, AB569481, AB569496, AB569527, AB569526, AB569529, AB569528, AB569525, AB569498, AB569497, AB569524, AB569499, AB569478, AB212282, AB212281, AB212284, AB212283, AB212280, AB212277, EF061119, AB212279, AB212278, HM172412, HM172288, HM172126, AB569477, AB569476, HM172186, HM172330, HM172385, HM172232, HM172109, JN605400, AB569563, JN605392, JN605396, AB569562, AB569559, AB569558, AB569561, AB569560, JN605372, JN605393, JN605397, JN605385, JN605373, JN605401, JN605380, JN605384, JN605388, JN605376, AB569557, AB569544, AB569543, AB569546, AB569545, AB569542, AB569531, AB569530, AB569541, AB569540, AB569547, AB569554, AB569553, AB569556, AB569555, AB569552, AB569549, AB569548, AB569551, AB569550, JN646735, JN646728, JN646694, JN646742, JN646721, JN646700, JN646693, JN646714, JN646707, JN646701, JN646695, JN646743, JN646709, JN646702, JN646736, JN646715, JN646708, JN646729, JN646722, JN646741, JQ906569, JQ906565, JQ906577, JQ906573, JQ906561, JQ906584, JQ906580, JQ906592, JQ906588, JQ906581, JN646720, JN646713, JN646734, JN646727, JN646706, JQ906589, JQ906585, JN646699, JN646692, JN646733, JN646726, JN646747, JN646740, JN646719, JN646698, JN646746, JN646712, JN646705, DQ997416, DQ997413, DQ997409, DQ997411, DQ997414, DQ997415, DQ997412, DQ997410, JN646739, JN646703, JN646696, JN646717, JN646710, JN646744, JN646723, JN646716, JN646737, JN646730, JN646724, JN646718, JN646711, JN646732, JN646725, JN646704, JN646738, JN646731, JN646697, JN646745, DQ376682, DQ376718, DQ376895, DQ376790, DQ376754, DQ376862, DQ376898, DQ376646, DQ376826, DQ376859, DQ376879, DQ376787, DQ376807, DQ376843, DQ376681, DQ376643, DQ376823, DQ376715, DQ376751, DQ376773, DQ376798, DQ376834, DQ376726, DQ376618, DQ376870, GU050359, GU050360, GU050361, GU050358, DQ376690, DQ376737, DQ376629, DQ376665, DQ376700, DQ376809, DQ376762, DQ376654, DQ376845, DQ376881, JQ794472, JQ794473, JQ794474, JQ794471, JQ794470, JQ794477, JQ794450, JQ794475, JQ794476, GU186663, GU186660, GU186657, DQ366304, DQ366303, GU186658, GU186661, GU186662, GU186659, GU186656, JQ794447, DQ376867, DQ376903, DQ376651, DQ376831, DQ376771, DQ376735, DQ376627, DQ376663, DQ376699, DQ376759, JQ794446, JQ794451, JQ794448, JQ794449, JQ794452, DQ376687, DQ376723, JQ794453, DQ376795, GU050357, HM145007, HM144838, HM145411, HM145580, HM144669, HM145345, HM145514, HM145176, HM144499, HM145242, HM145515, HM145684, HM144500, HM145346, HM144904, HM145073, HM144396, HM144736, HM144566, HM145683, HM144485, HM145331, HM144655, HM145162, HM145500, HM144777, HM144608, HM145669, HM144946, HM144824, HM144574, HM145081, HM144912, HM144744, HM144404, HM145588, HM144993, HM145250, HM145419, AB284991, AB284990, AB285091, AB285090, AB284989, AB284986, AB284985, AB284988, AB284987, AB285092, GU050364, AB285098, GU050362, GU050363, AB285097, AB285094, AB285093, AB285096, AB285095, AB304151, HM145338, HM145507, HM145169, HM144492, HM145676, HM144670, HM145177, HM145008, HM144839, HM144662, AB304148, AB304147, AB304150, AB304149, AB304146, HM145000, HM144831, AB304145, AB304144, AB593431, AB593430, AB593433, AB593432, AB593429, DQ366325, DQ366324, AB593428, DQ366326, AB593434, AB593449, AB593448, AB593451, AB593450, AB593447, AB593444, AB593435, AB593446, AB593445, DQ366323, EU931016, EU931013, EU931026, EU931027, EU931014, EU931017, EU931018, EU931015, EU931012, EU931025, DQ366320, DQ366319, DQ366322, DQ366321, EU931024, EU931023, EU931020, EU931021, EU931022, GU220598, GU220597, GU220600, GU220599, GU220596, AB593473, AB593472, AB593475, AB593474, GU220601, EF061123, EF061122, EF061125, EF061126, EF061120, GU220603, GU220602, EF061124, EF061121, AB593471, AB593482, AB593481, AB593460, AB593483, AB593480, AB593477, AB593476, AB593479, AB593478, AB593461, AB593468, AB593467, AB593470, AB593469, AB593466, AB593463, AB593462, AB593465, AB593464, EU931019, EU930911, EU930908, EU930909, EU930910, EU930913, EU930904, EU930901, EU930914, EU930915, EU930912, EU930917, EU930918, EU930931, EU930920, EU930919, EU930922, EU930923, EU930916, EU930921, EU930902, EU930891, DQ366310, EU930889, EU930890, DQ366309, DQ366306, DQ366305, DQ366308, DQ366307, EU930884, EU930905, EU930906, EU930903, EU930900, EU930907, EU930886, EU930887, EU930888, EU930885, EU930956, EU930961, EU930958, EU930959, EU930962, EU930941, EU930942, EU930963, EU930944, EU930957, EU930990, EU930991, EU930992, EU930989, EU930988, EU930995, EU930960, EU930993, EU930994, EU930943, EU930928, EU930925, EU930938, EU930939, EU930926, EU930929, EU930930, EU930927, EU930924, EU930937, EU930946, EU930947, EU930940, EU930945, EU930936, EU930935, EU930932, EU930933, EU930934, EU195418, EU195417, EU195420, EU195419, EU195416, EU195413, EU195396, EU195415, EU195414, EU195405, EU195412, EU195411, EU195398, EU195397, EU195410, EU195407, EU195406, EU195409, EU195408, EU195395, AB530990, AB530989, AB530992, AB530991, EF634344, EF634341, EF634340, EF634343, EF634342, AB530993, EU195392, EU195391, EU195394, EU195393, EU195390, AB530995, AB530994, EU195389, AB530996, HM145070, HM144393, HM144733, HM144563, HM145239, GQ923225, GQ923222, HM145408, HM145577, HM144901, HM144734, HM144564, HM145700, HM144902, HM145071, HM145409, HM145578, HM144394, HM145240, GQ923223, GQ923476, EU195404, GQ923474, GQ923475, EU195403, EU195400, EU195399, EU195402, EU195401, GQ923469, GQ923226, GQ923227, GQ923224, GQ923221, GQ923228, GQ923471, GQ923472, GQ923473, GQ923470, EF634339, DQ017517, DQ017516, GQ923340, DQ017506, DQ017502, DQ017514, DQ017505, DQ017503, DQ017515, GQ923339, GQ923348, GQ923337, GQ923346, GQ923347, GQ923334, GQ923333, GQ923338, GQ923335, GQ923336, DQ017513, DQ017508, DQ017511, DQ017504, DQ017512, DQ017507, DQ017510, DQ017509, GQ176126, GQ176125, GQ176124, GQ176127, GQ176128, GQ176121, HQ259228, GQ176123, GQ176122, DQ997217, DQ997213, DQ997214, EF634338, EF634337, DQ997211, DQ997215, DQ997216, DQ997212, DQ997218, HQ259227, HQ259230, HQ259229, HQ259232, HQ259231, GQ923345, GQ923344, GQ923341, GQ923342, GQ923343, HQ259233, HQ259224, HQ259223, HQ259226, HQ259225, HQ259222, HQ259235, HQ259234, HQ259221, HQ259236, EU980484, EU980485, EU980482, EF607858, EU980486, EU980503, EU980504, EU980505, EU980502, EU980481, EU980489, EF607859, EU980487, EU980488, EU980491, EU980483, EU980480, EU980492, EU980493, EF607860, GQ923310, GQ923311, GQ923396, GQ923313, GQ923312, GQ923315, GQ923316, GQ923309, GQ923314, GQ923395, EU980508, GQ923393, EU980506, EU980507, GQ923390, GQ923389, GQ923394, GQ923391, GQ923392, FJ357083, FJ357084, FJ357077, FJ357082, EU871917, EU871916, EF607888, EU871914, EU871915, FJ357080, EU871868, EU871873, EU871870, EU871871, EU871874, FJ357078, FJ357079, EU871875, FJ357081, EU871918, EU980496, EU980497, EU980498, EU980495, EU980494, EU980501, EU980490, EU980499, EU980500, FJ357076, FJ357073, FJ357070, EU871919, EU871920, FJ357071, FJ357074, FJ357075, FJ357072, FJ357069, HM145043, HM144555, HM145400, HM144725, HM145231, HM145569, HM144728, HM144558, HM145738, HM144896, HM144893, HM144559, HM145066, HM144897, HM144729, HM144389, HM145573, HM145062, HM145235, HM145404, HM145065, HM145024, HM144855, HM145410, HM145579, HM144686, HM145362, HM145531, HM145193, HM144516, HM145241, HM145403, HM145572, HM144388, HM145234, HM144903, HM145072, HM144395, HM144735, HM144565, HM145380, HM145549, HM145211, HM144534, HM145718, HM144659, HM145166, HM144997, HM144828, HM144704, HM145212, HM144535, HM144874, HM144705, HM145381, HM145042, HM144873, HM145550, HM145719, HM144489, HM144817, HM144648, HM145574, HM144986, HM145155, HM145493, HM145662, HM144478, HM145324, HM145405, HM145673, HM144898, HM145335, HM145504, HM144730, HM144390, HM145236, HM144560, HM145067, EU743317, EU743314, EU743315, EU743316, EU743319, GQ923289, GQ923286, EU743320, EU743321, EU743318, EU871837, EU871838, EU871840, EU871839, EU871842, EU871843, EU871836, EU871841, GQ923287, GQ923212, GQ923185, GQ923210, GQ923211, GQ923182, GQ923181, GQ923186, GQ923183, GQ923184, GQ923205, GQ923290, GQ923291, GQ923288, GQ923285, GQ923292, GQ923207, GQ923208, GQ923209, GQ923206, GQ923187, GQ923502, GQ923503, GQ923188, GQ923505, GQ923504, GQ923507, GQ923508, GQ923501, GQ923506, HM144900, HM144732, HM172379, HM172405, HM144562, HM145238, HM145407, HM145069, HM144392, HM172334, GU182148, GU182147, GU182150, GU182149, HM172294, HM172221, HM172097, HM172129, HM172177, HM145576, HM172088, HM172331, HM172203, HM172245, HM172381, DQ997170, DQ997164, HM172407, DQ997166, HM172160, HM144722, HM145228, HM145059, HM144890, HM144552, HM145735, HM172273, HM145397, HM145566, HM144892, HM144724, HM145716, HM145061, HM145230, HM145568, HM145737, HM144554, HM145399, HM145547, HM145645, HM145040, HM145307, HM145476, HM144871, HM144532, HM145378, HM144702, HM145209, FJ784872, FJ784807, FJ784823, FJ784791, FJ784855, FJ784839, GU182152, GU182151, GU182154, GU182153, FJ784775, FJ784792, FJ784856, FJ784888, FJ784776, FJ784808, FJ784871, FJ784887, FJ784824, FJ784840, DQ997165, AB612898, AB612913, AB612900, AB612899, AB612912, AB612909, AB612908, AB612911, AB612910, AB612901, AY676029, AY676025, AY676037, AY676033, AY676021, AB612903, AB612902, AB612905, AB612904, AB612907, AB275284, AB275283, AB275286, AB275285, AB274965, AB612888, AB612887, AB274964, AB274963, AB275287, AB262466, AB262465, AB612906, AB262467, AB262464, AB262461, AB262460, AB262463, AB262462, EU594353, EU594352, DQ465398, DQ465397, EU594351, EU594348, EU594347, EU594350, EU594349, DQ465399, DQ997168, DQ997169, DQ997163, DQ997167, DQ465404, DQ465401, DQ465400, DQ465403, DQ465402, EU594346, FJ784852, FJ784804, FJ784772, FJ784788, FJ784820, AY676045, AY676041, FJ784836, AY676049, FJ784884, HM172166, HM172216, HM172263, HM172119, HM172071, HM172449, FJ784868, HM172311, HM172360, JN852782, JN852779, JN852780, JN852781, JN852784, AY676052, AY676048, JN852785, JN852786, JN852783, DQ449641, DQ449642, EU871835, DQ449644, DQ449643, DQ449646, DQ449647, DQ449640, DQ449645, AY676044, DQ376901, HM745402, DQ376830, DQ376866, HM745401, HM745398, HM745397, HM745400, HM745399, DQ376650, AY676032, AY676028, AY676040, AY676036, AY676024, DQ376721, DQ376758, DQ376794, DQ376685, DQ104701, EU249547, DQ104703, EU249548, EU249546, GU186725, GU186726, EU249545, GU186728, EU249550, GQ257442, GQ257443, GQ257444, GQ257441, DQ435283, GQ257447, EU249549, GQ257445, GQ257446, GU186727, EU871832, EU871829, GU186464, GU186465, EU871830, EU871833, EU871834, EU871831, EU871828, GU186463, GU186730, GU186731, GU186724, GU186729, GU186462, GU186461, GU186458, GU186459, GU186460, HM745396, HM172178, HM172231, HM172306, HM172158, HM172105, HM172416, HM172307, HM172326, HM172389, HM145721, HM145045, HM144876, HQ285884, HQ285883, HM144707, HM145383, HM145552, HM145214, HM144537, HM172155, FJ784809, FJ784825, FJ784841, FJ784793, HM144969, HM145138, HM144461, HM144800, HM144631, FJ784777, HM172098, HM172325, HM172209, HM172229, HM172384, FJ784873, FJ784761, HM172422, FJ784857, GU324775, GU324774, GU324777, GU324776, GU324773, HM145022, HM144853, GU324772, GU324771, GU324778, HM172151, HM172200, HM745395, HM172290, HM172242, HM172391, HM172437, HM172100, HM172339, HM144684, GU182163, HQ285890, GU182165, GU182164, HQ285889, HQ285886, HQ285885, HQ285888, HQ285887, GU182166, HM145360, HM145529, HM145191, HM144514, HM145698, GU182168, GU182167, GU182170, GU182169, HM144691, HM145198, HM145029, HM144860, HM144521, HM145705, HM145028, HM145367, HM145536, HM145706, HM145030, HM144861, HM145538, HM145707, HM144692, HM145368, HM145537, HM145199, HM144522, HM144859, HM145534, HM145703, HM144519, HM145365, HM145038, HM145207, HM144530, HM144869, HM144700, HM145196, HM144520, HM145366, HM144690, HM145197, HM145535, HM144858, HM144689, HM145704, HM145027, HM145202, HM144525, HM144864, HM144695, HM145371, HM145025, HM144856, HM145540, HM145709, HM145033, HM144865, HM144696, HM145711, HM145034, HM145203, HM145541, HM145710, HM144526, HM145372, HM144687, HM145708, HM145031, HM145370, HM145539, HM144862, HM144523, HM145369, HM144693, HM145200, HM144524, HM145363, HM145532, HM145194, HM144517, HM145701, HM144694, HM145201, HM145032, HM144863, HM145376, JF965230, JF965325, JF965021, JF965262, JF965189, JF965061, JF965020, JF965150, JF965110, JF965062, JF965022, JF965263, JF965112, JF965063, JF965231, JF965151, JF965111, JF965326, JF965190, JF965289, JF965109, JF965060, JF965188, JF965149, JF965019, JF965299, JF965214, JF965288, JF965256, JF965324, JF965215, JF965177, JF965257, JF965300, JF965136, JF965261, JF965229, JF965084, JF965047, AJ849934, CY046120, CY046121, AM040281, AJ632268, CY046117, AB465628, CY046119, CY046118, CY046134, CY046138, AJ973609, HM145545, HM145714, AJ972921, CY046136, CY046135, CY046137, AJ971297, AB465623, GU727675, GU727674, GU727677, GU727676, JF965264, JF965191, JF965152, JF965232, JF965327, GU727678, AB465593, AB468064, AB465595, AB465621, AB468063, GU727680, GU727679, AB465626, GU727681, HM145358, HM145527, HM145189, HM144512, HM145696, EU263355, EU263354, EU263357, EU263356, HM144682, EU086251, EU086234, EU086235, EU086236, EU086259, HM145020, HM144851, EU086260, EU086262, EU263353, HM144872, HM144703, HM145695, HM145041, HM145210, HM145548, HM145717, HM144533, HM145379, HM145526, EU263350, HM145019, EU263352, EU263351, HM144850, HM144511, HM145357, HM144681, HM145188, AB473938, AB473937, AB125928, AB473939, GU186773, GU186772, GU186769, GU186770, GU186771, AB473940, AB277754, AB612885, AB277755, AB612886, AB612884, AB473941, AB251944, AB612883, AB473942, GU186774, HM144706, HM145213, HM145044, HM144875, HM144536, HM145720, EU086254, HM145382, HM145551, DQ997519, DQ997516, DQ997520, GU186775, GU186776, DQ997514, DQ997517, DQ997518, DQ997515, DQ997513, HM145018, HM144529, HM145375, HM144699, HM145206, HM145544, HM144867, HM144698, HM145713, HM145036, HM144868, HQ425335, HQ425336, HQ425338, HQ425337, HQ425334, HQ425331, HM145037, HQ425333, HQ425332, HM145205, HM145035, HM144866, HM145533, HM145702, HM144697, HM145373, HM145542, HM145204, HM144527, HM145364, HM145543, HM145712, HM144528, HM145374, HM145026, HM145195, HM144518, HM144857, HM144688, DQ366337, DQ366336, DQ366339, DQ366338, DQ366335, EU263347, EU263346, EU263349, EU263348, DQ366340, HM145187, HM144510, HM144849, HM144680, HM145356, DQ366342, DQ366341, HM145525, HM145694, EU263345, AY737306, AY737308, HM172432, AY737307, AY737305, AY737302, AY737301, AY737304, AY737303, HM172387, EU263342, HM172300, EU263344, EU263343, HM172140, HM172116, HM172321, HM172210, HM172230, GQ923180, HM145078, HM144401, HM144741, HM144571, HM145247, HM144960, HM144791, HM145416, HM145585, HM144909, HM144792, HM144623, HM145638, HM144961, HM145130, HM145468, HM145637, HM144453, HM145299, HM144622, HM145635, HM145014, HM145297, HM145466, HM144845, HM144506, HM145352, HM144676, HM145183, HM144451, HM145298, HM145467, HM145129, HM144452, HM145636, HM144621, HM145128, HM144959, HM144790, HM144432, HM145278, HM144602, HM145109, HM145447, HM144847, HM144678, HM145616, HM145016, HM144771, HM144572, HM145079, HM144910, HM144742, HM144402, HM145586, HM144940, HM145248, HM145417, HM145185, HM144962, HM144793, HM145522, HM145691, HM144624, HM145300, HM145469, HM145131, HM144454, HM145353, HM145523, HM145692, HM144508, HM145354, HM145015, HM145184, HM144507, HM144846, HM144677, HM145521, HM144577, HM145084, HM144915, HM144747, HM144407, HM145591, HM144899, HM145253, HM145422, HM145686, HM145010, HM144841, HM145518, HM145687, HM144672, HM145348, HM145517, HM145179, HM144502, HM144731, HM145414, HM145583, HM144399, HM145245, HM144956, HM145125, HM144448, HM144787, HM144618, HM145076, HM144391, HM145237, HM144561, HM145068, HM145406, HM144739, HM144569, HM145575, HM144907, HM145086, HM144409, HM144749, HM144579, HM145255, HM145013, HM144844, HM145424, HM145593, HM144917, HM144750, HM144580, HM145690, HM144918, HM145087, HM145425, HM145594, HM144410, HM145256, HM144675, HM145688, HM145011, HM145350, HM145519, HM144842, HM144503, HM145349, HM144673, HM145180, HM144504, HM145351, HM145520, HM145182, HM144505, HM145689, HM144674, HM145181, HM145012, HM144843, HM145509, HM145678, HM144494, HM145340, HM144942, HM145111, HM144434, HM144773, HM144604, HM145171, HM144398, HM145244, HM144568, HM145075, HM145413, HM144833, HM144664, HM145582, HM145002, HM145280, HM144567, HM145074, HM144905, HM144737, HM144397, HM145581, HM144923, HM145243, HM145412, HM145617, HM144941, HM144772, HM145449, HM145618, HM144603, HM145279, HM145448, HM145110, HM144433, HM145682, HM145005, HM145344, HM145513, HM144836, HM144497, HM145343, HM144667, HM145174, HM144498, HM145284, HM145453, HM145115, HM144438, HM145622, HM144668, HM145175, HM145006, HM144837, HM145512, HM145172, HM144495, HM144834, HM144665, HM145341, HM144906, HM144738, HM145510, HM145679, HM145003, HM144835, HM144666, HM145681, HM145004, HM145173, HM145511, HM145680, HM144496, HM145342, HM144755, HM145355, HM145524, HM145186, HM144509, HM145693, HM144582, HM145089, HM144920, HM144752, HM144679, HM145091, HM144414, HM144754, HM144584, HM145260, HM145017, HM144848, HM145429, HM145598, HM144412, HM144794, HM144625, HM145640, HM144963, HM145132, HM145470, HM145639, HM144455, HM145301, HM145471, HM145596, HM144964, HM145258, HM145427, HM144795, HM144456, HM145302, HM144626, HM145133, HM145508, HM145677, HM144493, HM145339, HM144967, HM145136, HM144459, HM144798, HM144629, HM145170, HM144415, HM145261, HM144585, HM145092, HM145430, HM144832, HM144663, HM145599, HM145001, HM145305, HM144627, HM145134, HM144965, HM144796, HM144457, HM145641, HM144922, HM145303, HM145472, HM145642, HM144966, HM144797, HM145474, HM145643, HM144628, HM145304, HM145473, HM145135, HM144458, GQ117174, GQ117175, GQ117172, GQ117169, GQ117176, GQ117155, GQ117156, GQ117157, GQ117154, GQ117171, GQ117196, GQ117193, GQ117194, GQ117195, GQ117198, GQ117173, GQ117170, GQ117199, GQ117200, GQ117153, GQ117138, GQ117139, GQ117152, GQ117141, GQ117140, GQ117143, GQ117144, GQ117137, GQ117142, GQ117151, GQ117160, GQ117149, GQ117158, GQ117159, GQ117146, GQ117145, GQ117150, GQ117147, GQ117148, GQ117255, GQ117256, GQ117249, GQ117254, GQ117245, GQ117244, GQ117241, GQ117242, GQ117243, GQ117252, GQ257433, GQ257438, GQ257435, GQ257436, GQ257439, GQ117250, GQ117251, GQ257440, GQ117253, GQ117246, GQ117203, GQ117204, GQ117205, GQ117202, GQ117201, GQ117208, GQ117197, GQ117206, GQ117207, GQ117232, GQ117229, GQ117226, GQ117247, GQ117248, GQ117227, GQ117230, GQ117231, GQ117228, GQ117225, DQ064482, AY233393, AY233389, AY233391, AY233394, AY233388, AB473546, AB473545, AY233387, AB473547, AY233392, EU026112, EU026113, EU026114, EU026111, EU026110, EU026117, AY233390, EU026115, EU026116, AB473544, GQ923177, GQ923174, AB268552, AB473548, GQ923175, GQ923178, GQ923179, GQ923176, GQ923173, AB268553, AB473541, AB473540, AB473543, AB473542, AB268556, AB268554, AB268557, AB268555, AB302788, DQ064508, DQ064535, DQ064454, DQ064373, DQ064562, EU742654, EU742655, EU742656, EU742653, DQ064427, DQ064455, DQ064374, DQ064401, DQ064428, DQ064509, DQ064481, DQ064400, DQ064536, DQ064563, EU742652, EU026103, EU026104, EU735841, EU026106, EU026105, EU026108, EU026109, EU026102, EU026107, EU735840, EU742659, EU735838, EU742657, EU742658, EU735835, EU735834, EU735839, EU735836, EU735837, HM145009, HM144840, HM145420, HM145589, HM144671, HM145347, HM145516, HM145178, HM144501, HM145251, HM145421, HM145590, HM144406, HM145252, HM144913, HM145082, HM144405, HM144745, HM144575, HM145685, AY854190, AY856863, AY854191, AY856864, AY856862, GU596987, GU596986, AY856861, GU596988, AY856865, DQ997532, DQ997533, DQ997534, DQ997536, DQ997531, DQ997530, AY856866, DQ997535, DQ997529, HM144785, HM144616, HM145631, HM144954, HM145123, HM145461, HM145630, HM144446, HM145292, HM145462, HM145632, HM144955, HM145294, HM145463, HM144786, HM144447, HM145293, HM144617, HM145124, HM144953, HM145290, HM145459, HM145121, HM144444, HM145628, HM144576, HM145083, HM144914, HM144746, HM144614, HM145122, HM144445, HM144784, HM144615, HM145291, HM144952, HM144783, HM145460, HM145629, GU596985, DQ232608, GQ923537, DQ234076, DQ232606, GQ923534, GQ923533, GQ923538, GQ923535, GQ923536, DQ230522, GQ923546, GQ923547, GQ923544, GQ923541, GQ923548, DQ230524, DQ232610, DQ234074, DQ234078, GQ923539, GQ117268, GQ117265, GQ117266, GQ117267, GQ117270, GQ257437, GQ257434, GQ117271, GQ117272, GQ117269, GQ923558, GQ923559, GQ923540, GQ923561, GQ923560, GQ923563, GQ923564, GQ923557, GQ923562, GQ162789, GQ162787, GQ227611, GQ227610, GQ162788, GQ162791, GQ162792, GQ162790, GQ162786, GQ227607, GU596982, GU596981, GU596984, GU596983, GQ227609, GQ227606, GQ227604, GQ227608, GQ227605, GQ162793, GQ923421, GQ923426, GQ923423, GQ923424, GQ923427, GQ923542, GQ923543, GQ923428, GQ923545, GQ923422, GQ923431, GQ923432, GQ923433, GQ923430, GQ923429, GQ923436, GQ923425, GQ923434, GQ923435, GQ184327, GQ203124, GQ184332, GQ169500, GQ203123, JF800145, JF800146, GQ203122, JF800148, GQ219713, GQ219714, JF800147, JF800150, JF800151, JF800144, JF800149, AB615239, AB615238, AB615241, AB615240, AB615237, AB615234, AB615236, AB615235, HM849010, HM849007, HM849004, HM849005, HM849008, HM849009, HM849006, HM849003, GQ907326, GQ907331, GQ907328, GQ907329, GQ907332, GQ907333, GQ907330, GQ907327, AB523763, AB523762, AB523761, AB523758, AB523757, AB523760, AB523759, AB523756, GQ907334, GQ907339, GQ907336, GQ907337, GQ907340, GQ907341, GQ907335, GQ907344, GQ907345, GQ907346, GQ907343, GQ907342, GQ907349, GQ907338, GQ907347, GQ907348, AY950277, AY950284, AY950270, EU329175, EU329174, EU329177, EU329176, AY950263, AY950256, AY950235, AY950242, AY950249, GU186786, GU186787, HM145592, GU186789, GU186788, GU186791, GU186792, GU186785, GU186790, HM145423, HM145634, HM144916, HM145296, HM145465, HM144748, HM144408, HM145254, HM144578, HM145085, HM172269, EU329187, EU329188, EU329185, EU329186, EU329189, EU329179, EU329178, EU329181, EU329180, EU329184, HM172226, HM172084, HM172146, HM172190, HM172314, EU329182, EU329183, HM172403, HM172423, HM145114, HM144437, HM144776, HM144607, HM145283, HM144992, HM144823, HM145452, HM145621, HM144945, HM144825, HM144656, HM145672, HM144994, HM145163, HM145501, HM145670, HM144486, HM145332, HM144654, HM145619, HM144938, HM145281, HM145450, HM144769, HM144430, HM145276, HM144600, HM145107, HM144435, HM145330, HM145499, HM145161, HM144484, HM145668, HM144605, HM145112, HM144943, HM144774, HM144440, HM145286, HM144610, HM145117, HM145455, HM144805, HM144636, HM145624, HM144974, HM144779, HM144611, HM145118, HM144949, HM144780, HM144441, HM145625, HM144948, HM145287, HM145456, HM145143, HM144996, HM144827, HM145480, HM145649, HM144658, HM145334, HM145503, HM145165, HM144488, HM145311, HM145481, HM145650, HM144466, HM145312, HM144973, HM145142, HM144465, HM144804, HM144635, HM145445, HM144581, HM145088, HM144919, HM144751, HM144411, HM145595, HM144998, HM145257, HM145426, HM145597, HM144921, HM144753, HM145418, HM145587, HM144583, HM145259, HM145428, HM145090, HM144413, HM144829, HM145415, HM145584, HM144400, HM145246, HM144958, HM145127, HM144450, HM144789, HM144620, HM145077, HM144490, HM145336, HM144660, HM145167, HM145505, HM144740, HM144570, HM145674, HM144908, HM145150, HM144473, HM144812, HM144643, HM145319, HM145064, HM144895, HM145488, HM145657, HM144981, HM144814, HM144645, HM145614, HM144983, HM145152, HM145490, HM145659, HM144475, HM145321, HM144727, HM145656, HM144911, HM145318, HM145487, HM144743, HM144403, HM145249, HM144573, HM145080, HM144472, HM145402, HM145571, HM145233, HM144557, HM145740, HM144642, HM145149, HM144980, HM144811, GQ257379, GQ257380, GQ257373, GQ257378, DQ021642, FJ517285, FJ517282, FJ517283, FJ517284, GQ257376, EU871884, EU871889, EU871886, EU871887, EU871890, GQ257374, GQ257375, EU871891, GQ257377, FJ517286, EU743467, EU743468, EU743469, EU743466, EU743465, EU743472, GQ923249, EU743470, EU743471, EU871883, EU871880, EU871877, FJ517287, FJ517288, EU871878, EU871881, EU871882, EU871879, EU871876, FJ517301, DQ021624, FJ517299, FJ517300, EU743502, EU743501, EU743505, EU743503, EU743504, FJ517296, EU743513, EU743514, EU743511, EU743508, EU743515, FJ517297, FJ517298, DQ021630, DQ021722, EU743506, EU743532, EU743530, DQ021714, EU743531, EU743533, EU871888, EU871885, EU743534, EU743535, DQ021564, DQ021716, FJ517290, EU743507, FJ517292, FJ517291, FJ517294, FJ517295, FJ517289, FJ517293, GQ923246, FJ357133, FJ357134, FJ357131, FJ357128, FJ357135, FJ357122, FJ357123, FJ357124, FJ357121, FJ357130, GQ257482, GQ257479, GQ257480, GQ257481, GQ257484, FJ357132, FJ357129, GQ257485, GQ257486, FJ357120, EU026083, EU026084, EU026097, EU026086, EU026085, EU026088, EU026089, EU026082, EU026087, EU026096, FJ357127, EU026094, FJ357125, FJ357126, EU026091, EU026090, EU026095, EU026092, EU026093, GQ923243, GQ923244, GQ923237, GQ923242, FJ686742, FJ686741, FJ686738, FJ686739, FJ686740, GQ923240, GQ923245, GQ923250, GQ923247, GQ923248, GQ923251, GQ923238, GQ923239, GQ923252, GQ923241, FJ686743, FJ357138, FJ357139, FJ357140, FJ357137, FJ357136, FJ357143, GQ257483, FJ357141, FJ357142, GQ257494, GQ257491, GQ257488, FJ686744, FJ686745, GQ257489, GQ257492, GQ257493, GQ257490, GQ257487, GQ923271, GQ923272, GQ923273, GQ923270, GQ923269, GQ923276, FJ686734, GQ923274, GQ923275, GQ923284, GQ923281, GQ923278, GQ923163, GQ923164, GQ923279, GQ923282, GQ923283, GQ923280, GQ923277, FJ686731, FJ686728, FJ686729, FJ686722, FJ686727, FJ686718, FJ686717, FJ686714, FJ686715, FJ686716, FJ686725, FJ686730, FJ686735, FJ686732, FJ686733, FJ686736, FJ686723, FJ686724, FJ686737, FJ686726, DQ017491, DQ017492, DQ017486, DQ017499, DQ017500, HM849021, HM849018, DQ017495, DQ017501, DQ017493, DQ017498, DQ017494, EU094470, DQ017490, DQ017488, DQ017497, DQ017489, DQ017496, DQ017487, HM849019, GU186538, GQ923161, GU186536, GU186537, GQ923158, GQ923157, GQ923162, GQ923159, GQ923160, GU186531, HM849022, HM849023, HM849020, HM849017, HM849024, GU186533, GU186534, GU186535, GU186532, FJ686719, DQ021647, DQ021726, GU052843, GU052844, EU743496, EU743498, EU743499, EU743497, EU743495, GU052842, GQ923571, GQ923572, GQ923565, GQ923570, GU052841, GU052840, GU052837, GU052838, GU052839, EU743500, EU743457, EU743462, EU743459, EU743460, EU743463, EU743509, EU743510, EU743464, EU743512, EU743458, GQ923215, GQ923216, GQ923217, GQ923214, GQ923213, GQ923220, EU743461, GQ923218, GQ923219, EF564746, EF418187, EF212862, EF221765, EF491876, EF517399, EF392844, EF405825, EF530048, FJ031998, FJ032005, FJ032004, FJ686720, FJ686721, FJ032003, FJ032000, FJ031999, FJ032002, FJ032001, GQ923457, GQ923450, GQ923451, GQ923448, GQ923445, GQ923452, GQ923567, GQ923568, GQ923569, GQ923566, GQ923447, GQ923456, GQ923453, GQ923454, GQ923455, GQ923458, GQ923449, GQ923446, GQ923459, GQ923460, EU026018, EU026015, EU026027, EU026028, EU026016, EU026019, EU026020, EU026017, EU026014, EU026026, EU026035, EU026036, EU026029, EU026034, EU026025, EU026024, EU026022, EU026023, EF607908, EU026021, EU743584, EU743589, EU743586, EU743587, EU743590, EU743577, EU743578, EU743591, EU743580, EU743585, EU743594, EU743595, EU743596, EU743593, EU743592, EU743599, EU743588, EU743597, EU743598, EU980517, EU980518, GQ257470, EU980520, EU980519, EU980522, EU980523, EU980516, EU980521, GQ257469, GQ257478, GQ257467, GQ257476, GQ257477, GQ257464, GQ257463, GQ257468, GQ257465, GQ257466, GQ257459, EU026042, EU026043, EU026040, EU026037, EU026044, EU026031, EU026032, EU026033, EU026030, EU026039, GQ257458, GQ257455, GQ257456, GQ257457, GQ257460, EU026041, EU026038, GQ257461, GQ257462, EU743579, EU743523, FJ517318, EU743521, EU743522, FJ517315, FJ517314, FJ517319, FJ517316, FJ517317, EU743516, FJ517272, FJ517273, FJ517270, FJ517267, FJ517274, EU743518, EU743519, EU743520, EU743517, FJ517320, EU743539, EU743536, EU743537, EU743538, EU743541, EU871872, EU871869, EU743542, EU743543, EU743540, EU871893, EU871894, FJ517321, EU871896, EU871895, EU871898, EU871899, EU871892, EU871897, GU066782, GU066781, EU743574, EU743575, GU066780, DQ864508, DQ864506, DQ864509, GU066779, EU743573, EU743582, EU743583, EU743576, EU743581, EU743572, EU743571, EU743568, EU743569, EU743570, DQ864507, EU743552, EU743557, EU743554, EU743555, EU743558, FJ517268, FJ517269, EU743559, FJ517271, EU743553, EU743546, EU743547, EU743548, EU743545, EU743544, EU743551, EU743556, EU743549, EU743550, FJ686709, FJ686706, FJ686707, FJ686708, FJ686711, FJ686814, FJ686811, FJ686712, FJ686713, FJ686710, GQ257504, GQ257505, GQ257518, GQ257507, GQ257506, GQ257509, GQ257510, GQ257503, GQ257508, FJ686812, FJ686809, FJ686702, FJ686807, FJ686808, FJ686699, FJ686698, FJ686703, FJ686700, FJ686701, FJ686802, FJ686815, FJ686816, FJ686813, FJ686810, FJ686817, FJ686804, FJ686805, FJ686806, FJ686803, EU026066, EU026071, EU026068, EU026069, EU026072, EU026059, EU026060, EU026073, EU026062, EU026067, EU026076, EU026077, EU026078, EU026075, EU026074, EU026081, EU026070, EU026079, EU026080, EU026061, GQ257515, GQ257512, GQ257525, GQ257526, GQ257513, GQ257516, GQ257517, GQ257514, GQ257511, GQ257524, EU026064, EU026065, EU026058, EU026063, GQ257523, GQ257522, GQ257519, GQ257520, GQ257521, FJ686704, FJ686764, FJ686765, FJ686766, FJ686763, FJ686762, FJ686769, FJ686758, FJ686767, FJ686768, FJ686777, FJ686774, FJ686771, FJ686784, FJ686785, FJ686772, FJ686775, FJ686776, FJ686773, FJ686770, FJ686755, FJ686752, FJ686753, FJ686746, FJ686751, GQ257475, GQ257474, GQ257471, GQ257472, GQ257473, FJ686749, FJ686754, FJ686759, FJ686756, FJ686757, FJ686760, FJ686747, FJ686748, FJ686761, FJ686750, FJ357115, FJ357112, FJ357113, FJ357114, FJ357117, GQ257499, GQ257496, FJ357118, FJ357119, FJ357116, FJ686795, FJ686796, FJ686705, FJ686798, FJ686797, FJ686800, FJ686801, FJ686794, FJ686799, GQ257497, FJ686793, FJ686782, FJ686791, FJ686792, FJ686779, FJ686778, FJ686783, FJ686780, FJ686781, FJ686786, GQ257500, GQ257501, GQ257498, GQ257495, GQ257502, FJ686788, FJ686789, FJ686790, FJ686787, EU094471, EU743561, EU743562, EU743564, EU743563, EU743566, EU743567, EU743560, EU743565, AB636527, AB636526, AB636528, AB636525, GU050409, GU050406, EU441923, EU441922, GU050407, GU050410, GU050411, GU050408, GU050405, EU441924, AB636522, AB636521, AB636524, AB636523, EU441926, EU441925, EU441929, EU441927, EU441928, GQ923410, GQ923411, GQ923408, GQ923405, GQ923412, GQ923303, GQ923304, GQ923305, GQ923302, GQ923407, GQ923296, GQ923293, GQ923294, GQ923295, GQ923298, GQ923409, GQ923406, GQ923299, GQ923300, GQ923301, AB490823, AB490824, GQ923308, AB490830, GQ923306, GQ923307, AB490829, AB490826, AB490825, AB490828, AB490827, GU050412, EU743296, EU743293, EU743294, EU743295, EU743298, HM144999, HM144830, EU743299, EU743300, EU743297, GQ923486, GQ923487, GQ923484, GQ923489, GQ923488, GQ923491, GQ923492, GQ923485, GQ923490, HM144661, EF592492, EU094477, EF592494, EF592493, EU094476, EU094473, EU094472, EU094475, EU094474, EF592495, HM145337, HM145506, HM145168, HM144491, HM145675, EF592497, EF592496, EF592499, EF592498, FJ349251, FJ349248, FJ349250, FJ349249, GQ923481, GQ923478, EU880348, EU880349, GQ923479, GQ923482, GQ923483, GQ923480, GQ923477, EU880347, FJ349253, FJ349254, FJ349247, FJ349252, EU880346, EU880345, EU880342, EU880343, EU880344, GQ923297, GU186745, GU186748, GU186747, GU186750, GU186752, GU186751, JN860171, JN860172, JN860173, JN860170, GU186746, GU186749, JN860169, JN860174, EU930986, EU930985, EU930980, EU930987, EU930966, EU930965, EU930968, EU931011, EU931010, EU931009, EU930984, EU930983, EU930982, EU930981, EU930967, HM050398, HM050392, HM050393, HM050397, JN860175, JN860176, HM050396, EU930970, EU930969, EU930964, EU930971, GU595163, HM050394, HM050395, EU084937, EU084938, AY296071, EU084936, AY300942, AY300968, AY300992, AY300983, EU084939, AY300933, AY300959, EU931004, AF250476, AF250477, AF250482, AF250483, AF250481, AF250478, AF250479, AF250480, AB593436, AB593437, AB593438, EU931008, EU931007, EU931006, EU931005, AB593443, AB593442, AB593439, AB593440, AB593441, HM570063, HM570058, HM570061, HM570064, HM570065, EU500853, EU500854, EU500855, EU500852, EU500849, EU500850, EU500851, EU500856, HM570060, HM570059, HM570062, AY296077, EU084943, AY300939, EU084942, AY075036, EU084940, EU084941, EU084951, AY296080, EU084952, EU084950, AY300965, AY300989, EU084949, AY075035, AY075033, GU186688, AY075034, GU186689, GU186691, GU186690, EU743310, EU743309, EU743308, EU743307, FJ357053, FJ357056, FJ357055, FJ357058, FJ357060, FJ357059, FJ357054, FJ357050, FJ357045, FJ357048, FJ357051, FJ357062, FJ357065, FJ357052, EU743312, EU743311, EU743306, EU743313, FJ357047, FJ357046, FJ357049, FJ357063, FJ357037, FJ357040, FJ357039, FJ357042, FJ357057, FJ357044, FJ357043, FJ357066, FJ357061, FJ357064, FJ357067, FJ357038, FJ357041, FJ357068, AB284321, AB284322, AB284323, AB284324, AB523772, AB523773, AB523774, AB284328, AB284325, AB284326, AB284327, AB523775, FM174396, FM174397, AB523779, AB523776, AB523777, AB523778, HM172401, HM172409, AB450647, HM172356, HM172182, HM172238, HM172115, AB450631, AB450523, AB450507, GU186652, AB450539, AB450609, AB450585, AB450563, GU186760, HM172270, HM172152, GU186759, GU186756, GU186753, GU186758, HM172175, HM172441, HM172304, HM172133, HM172402, HM172227, HM172107, HM172322, GU186649, GU186637, GU186638, GU186639, GU186632, GU186633, GU186634, GU186635, AB569491, AB569486, AB569485, AB569484, AB569487, AB569490, AB569489, AB569488, GU186654, GU186655, GQ227556, GU186653, GU186650, GU186651, GU186648, GQ227555, GQ227558, GQ227557, GU186636, GQ227554, GQ227552, GQ227553, GQ227551, AM914027, AM914029, AM914030, AM914028, FM174403, FM174404, AM914026, EU430499, EU430497, EU430502, EU430503, EU430498, EU430500, EU430501, EU430496, AM914019, AM914020, FM174399, AM914017, FM174398, AM914016, AM914018, FM174400, AM914024, AM914025, FM174402, AM914022, FM174401, AM914021, AM914023, EU430505, EU492506, EU492524, EU492518, EU492492, EU492534, EU492504, EU492498, EU492512, GU186757, GU186754, GU186755, EU492488, EU492530, EU492500, EU492494, EU430510, EU430507, EU430506, EU430504, EU430509, EU430508, EU430511, HQ200450, EU492528, EU492522, EU492516, EU492510, HQ200455, HQ200456, HQ200457, HQ200451, HQ200453, AY518367, HQ200454, HQ200452, AY518366, AY518362, AY518365, AY518361, AY518363, AY518364, GQ122448, GQ401161, GQ401160, GQ122388, GQ122450, GQ122449, GQ401158, GQ401163, GQ401162, AY518360, GQ401159, GQ401157, GQ401164, GQ122408, JF965285, JF965044, JF965082, JF965211, JF965318, JF965253, JF965133, JF965254, JF965286, JF965045, JF965174, JF965212, JF965319, GU727656, GU727655, GU727654, GU727659, GU727658, GU727657, JF965317, JF965081, JF965132, JF965173, JF965252, JF965284, JF965043, GQ122465, GQ122463, GQ122410, GU727651, GU727650, GQ122464, GQ122390, GQ122452, GQ122453, GQ122451, GQ122462, GQ122461, GQ122460, JF965213, JF965298, JF965255, JF965083, JF965134, JF965175, JF965287, JF965176, GU727653, GU727652, JF965046, JF965094, JF965135, GU727660, JF965244, JF965311, JF965203, JF965075, JF965035, JF965276, JF965164, JF965275, JF965243, JF965310, JF965124, JF965074, JF965034, JF965166, JF965126, JF965076, JF965080, JF965131, JF965172, JF965036, JF965204, JF965165, JF965125, JF965277, JF965245, JF965312, JF965309, JF965201, JF965162, JF965057, JF965274, JF965242, JF965122, JF965241, JF965308, JF965200, JF965072, JF965032, JF965273, JF965073, JF965033, JF965259, JF965202, JF965163, JF965123, JF965227, JF965147, JF965107, JF965058, JF965322, JF965186, JF965217, JF965042, JF965208, JF965169, JF965128, JF965281, JF965249, JF965314, JF965095, JF965337, JF965207, JF965168, JF965039, JF965280, JF965248, JF965282, JF965041, JF965079, JF965209, JF965315, JF965250, JF965130, JF965129, JF965078, JF965040, JF965171, JF965210, JF965170, JF965137, JF965093, JF965037, JF965333, JF965206, JF965167, JF965278, JF965316, JF965251, JF965283, JF965246, JF965313, JF965205, JF965260, JF965228, JF965323, JF965127, JF965077, JF965038, JF965187, JF965018, JF965279, JF965247, JF965148, JF965108, JF965059, JF965234, JF965329, JF965193, JF965066, JF965025, JF965266, JF965154, GU727673, GU727672, GU727671, JF965114, JF965065, JF965024, JF965156, JF965116, JF965089, JF965236, JF965331, JF965195, JF965026, JF965194, JF965155, JF965115, JF965267, JF965235, JF965330, JF965183, JF965144, JF965104, JF965296, JF965224, JF965336, JF965097, GU727663, GU727662, GU727661, JF965054, GU727665, GU727664, GU727667, GU727666, JF965265, GU727670, GU727669, GU727668, JF965233, JF965113, JF965064, JF965023, JF965328, JF965192, JF965153, JF965268, JF965159, JF965119, JF965069, JF965239, JF965307, JF965198, JF965029, JF965185, JF965146, JF965106, JF965258, JF965226, JF965321, JF965031, JF965272, JF965240, JF965161, JF965121, JF965071, JF965335, JF965070, JF965030, JF965271, JF965199, JF965160, JF965120, JF965088, JF965055, JF965269, JF965184, JF965145, JF965105, JF965237, JF965117, JF965067, JF965027, JF965332, JF965196, JF965157, JF965238, JF965306, JF965197, JF965091, JF965056, JF965270, JF965158, JF965297, JF965225, JF965305, JF965118, JF965068, JF965028

Chicken:

EU086241, DQ064553, DQ064526, EU086238, EU086237, EU086240, EU086239, DQ064391, DQ064472, HQ117883, DQ064418, DQ064499, DQ064364, DQ064445, EU086242, EU086263, EU086261, EU086258, EU081868, EU081867, EU081866, EU081865, EU086256, EU086244, EU086243, EU086248, EU086245, EU086253, EU086250, HQ117884, HM172188, HM172157, HM172279, HM172259, HM172370, HM172323, HM172079, AY724258, AY724264, AY724263, AY724257, AY724251, AY724261, AY724260, HM172424, HQ117889, HQ117890, GU186564, HQ117888, HQ117885, HQ117886, HQ117887, GU186559, GU186558, GU186561, GU186560, GU186563, GU186562, GU186557, JN869547, JN869548, JN869549, JN869546, JN869543, JN869544, JN869545, JN869538, JN869539, JN869540, JN869537, JN869550, JN869535, JN869536, DQ064470, DQ064415, DQ064388, DQ064469, DQ064442, DQ064523, DQ064496, DQ064361, DQ064443, DQ064416, DQ064389, DQ064362, DQ064551, DQ064524, DQ064497, JN869541, EU086231, EU086226, EU086229, EU086232, DQ366333, DQ366334, EU086233, EU081870, EU081869, EU081864, EU081871, EU086228, EU086227, EU086230, DQ366332, DQ064363, DQ064444, DQ064417, DQ064498, JN869542, DQ064552, DQ064525, DQ366329, DQ366330, DQ366331, DQ366328, DQ064390, DQ064471, DQ366327, AY724259, EU532036, EU532055, GQ202041, EU346941, EU532061, EU573941, EU532044, GQ202037, GQ202039, GQ202038, GQ202042, GQ202040, GQ202043, GQ202036, EU882861, GQ202058, FJ534548, FJ534549, GQ202059, GQ202056, GQ202062, GQ202057, GQ202045, GQ202044, EU914201, GQ202048, GQ202047, GQ202046, GQ202049, DQ064555, AY950251, AY950244, AY950237, AY950230, AY950279, AY950272, AY950265, FJ534538, FJ534544, FJ534539, FJ534545, AY950258, FJ534543, FJ534542, DQ064475, DQ064420, DQ064393, DQ064474, DQ064447, DQ064528, DQ064501, DQ064366, DQ064448, DQ064421, DQ064394, DQ064367, DQ064556, DQ064529, DQ064502, DQ343150, DQ351865, DQ349118, DQ351868, DQ064473, DQ351871, DQ351875, FJ499470, FJ499463, FJ499469, FJ499467, DQ351859, DQ351862, FJ499468, DQ064392, DQ351866, DQ349116, DQ351860, DQ343152, DQ351872, DQ351873, DQ351867, DQ064365, DQ064446, DQ064419, DQ064500, DQ351861, DQ064554, DQ064527, FJ499464, HM172366, HM172324, HM172081, HM172411, DQ349117, DQ351858, DQ351863, GQ202061, GQ202060, GQ202063, HM172271, HM172217, HM172197, HM172137, DQ351864, GQ202053, GQ202052, GQ202055, FJ534547, FJ499466, FJ499465, FJ534546, DQ351874, DQ351869, DQ343151, DQ351870, GQ202054, GQ202051, GQ202050, EU742893, AY240883, EU742891, EU742894, FR687293, FR687294, EU742895, EU743104, EU743103, GU186483, EU743105, EU742890, EU742889, EU742892, FR687260, FR687256, FR687267, FR687261, FR687279, AB465627, FR687272, FR687284, FR687280, FR687257, FR687268, FR687283, FR687291, FR687296, FR687271, EU743101, JN869514, JN869515, JN869516, DQ064463, DQ064436, DQ064409, DQ064382, JN869520, EF070740, EF070739, JN869519, JF715008, JN869517, JN869518, DQ064355, HM172373, HM172335, HM172096, HM172421, EU743100, EU743099, EU743102, DQ064544, DQ064517, DQ064490, HM172272, HM172223, HM172169, HM172122, JF965291, JF965050, JF965096, JF965219, JF965139, JF965178, JF965301, JF965220, JF965292, JF965051, JF965302, JF965100, JF965140, JF965179, JF965099, GQ122442, GQ122443, GQ122444, GQ122543, GQ122423, GQ122541, GQ122542, GQ122446, JF965049, JF965085, GQ122447, GQ122387, GQ122407, GQ122445, JF965086, JF965320, JF965223, JF965295, JF965182, JF965092, JF965103, JF965143, AB465620, AB465594, AB465622, AB465592, AB465625, AB465629, AB465624, JF965053, JF965221, JF965293, JF965052, JF965303, JF965101, JF965141, JF965180, JF965304, JF965222, JF965294, JF965181, JF965087, JF965102, JF965142, EF070738, AY737287, AY737288, AY737289, AY737286, AY737299, AY737298, AY737300, DQ064546, DQ064519, DQ064492, AY737285, AY737290, AY737291, AY737292, AY737297, AY609311, AY609312, AY609313, AY609310, DQ064383, DQ064464, AY609309, AY737294, AY737295, AY737296, AY737293, AY609314, AY609315, AY609316, DQ064357, DQ064467, DQ064549, DQ064522, DQ064386, DQ064359, DQ064440, DQ064413, DQ064387, DQ064468, DQ064550, DQ064414, DQ064495, DQ064360, DQ064441, DQ064494, DQ064548, DQ064520, DQ064493, DQ064465, DQ064438, DQ064411, DQ064384, DQ064466, DQ064547, DQ064521, DQ064385, DQ064358, DQ064439, DQ064412, GQ176135, GQ176132, AJ620347, GQ176134, GQ176131, GQ176136, GQ176133, AJ620349, AJ619676, AJ619678, AJ620352, AJ620348, AJ619677, AJ620350, GQ176130, EF070736, HM172406, HM172388, EF070737, EF070733, EF070735, EF070734, HM172124, HM172286, GQ176129, HM172187, HM172313, HM172075, HM172228, FM177116, EU874898, EU874900, EU874901, EU874899, EU874902, EU874903, EU874897, DQ064356, DQ064437, DQ064410, DQ064491, EU874904, DQ064545, DQ064518, FM177139, FM177121, FM177122, FM177123, FM177120, FM177117, FM177118, FM177119, FM177136, FM177137, FM177138, FM177135, FM177132, FM177133, FM177134, GQ148868, GQ140283, GQ120553, GQ140269, GQ120539, GQ148825, GQ148853, GQ140264, GQ148863, GQ140278, GQ148854, GQ148840, GQ120540, GQ148826, GQ148839, GQ140281, GQ120551, GQ148838, GQ148866, EF492248, EF492334, GQ140267, GQ148867, GQ140282, GQ120552, GQ140268, GQ120538, GQ148824, GQ148852, GQ120548, GQ148856, GQ140272, GQ148871, GQ148828, GQ120555, GQ148842, GQ120542, GQ148829, GQ148857, EF492410, GQ120543, GQ140286, GQ120556, GQ148843, GQ140285, EF492406, EF492377, EF492348, GQ148849, GQ148835, GQ120535, GQ148821, EF492336, GQ140271, GQ148870, EF492250, EF492227, EF492308, EF492279, EF492265, DQ683044, GQ140265, EF492298, EF492367, EF492242, EF492327, GQ120536, GQ148822, GQ148850, GQ148836, GQ148864, GQ140279, GQ120549, EF492396, DQ683041, EF492403, EF492374, EF492262, EF492240, EF492324, EF492295, EF492247, EF492333, EF492425, EF492276, EF492345, EF492224, EF492305, GQ140266, EF492329, EF492300, EF492267, EF492243, EF492427, EF492398, EF492369, EF492225, EF492306, EF492277, EF492346, DQ683046, EF492404, EF492375, DQ683045, GQ120537, GQ148823, GQ148851, GQ148837, GQ148865, GQ140280, GQ120550, EF492328, EF492299, EF492266, EF501983, EF492426, EF492397, EF492368, EF492381, DQ991321, DQ991329, DQ991322, DQ991320, DQ991327, DQ991319, DQ991328, DQ991332, FJ610115, FJ610114, DQ991324, DQ991330, DQ991331, DQ991323, DQ991326, GQ148875, GQ140290, GQ120560, GQ140276, GQ120546, GQ148832, GQ148860, DQ991325, DQ991317, DQ991318, GQ148861, GQ148847, GQ120547, GQ148833, FJ610113, HM172134, HM172275, FJ384748, HM172171, HM172352, HM172092, HM172234, FJ384750, FJ384751, FJ384759, FJ384758, FJ384756, FJ384749, FJ384757, HM172368, FJ610112, DQ064561, DQ064534, FJ610109, FJ610108, FJ610111, FJ610110, DQ064399, DQ064480, HM172417, DQ064426, DQ064507, DQ064372, DQ064453, EF492286, EF492254, EF492340, EF492315, EF492384, EF492355, EF492234, GQ148844, GQ120544, GQ148830, GQ120557, GQ140273, GQ148872, GQ140287, EF492413, EF492269, DQ683032, EF492411, EF492283, EF492352, EF492231, EF492312, EF492284, EF492253, EF492339, EF492313, EF492382, EF492353, EF492232, GQ148858, EF492236, EF492317, EF492288, EF492357, DQ683047, EF492415, EF492386, GQ140289, GQ120559, GQ148846, GQ148874, EF492256, EF492331, GQ140275, EF492272, GQ148845, GQ120545, GQ148831, GQ120558, GQ140274, GQ148873, GQ140288, EF492221, EF492330, EF492301, EF492370, GQ148859, EF492428, EF492399, AY684707, AY684708, AY684709, AY684706, AY684703, AY684704, AY684705, AY770079, AY770081, AY770078, AY770082, AY684710, AY770084, AY770083, FJ784867, GU186674, GU186673, GU186676, GU186675, GU186678, GU186677, GU186672, FJ784787, FJ784771, FJ784883, FJ784851, FJ784835, FJ784819, FJ784803, AY770077, DQ997137, DQ997133, DQ997135, DQ997131, DQ997119, DQ997117, DQ997132, HM172367, HM172333, HM172074, HM172425, DQ997134, DQ997138, DQ997136, DQ997115, EU365371, EU365372, EU365373, EU365370, AY770080, EU365368, EU365369, DQ997118, DQ997122, DQ997116, DQ997120, EU365374, EU365375, DQ997121, AY950276, AY950269, AY950234, AY950283, AY950247, AY950240, AY950261, DQ064557, DQ064530, DQ064503, AY950262, AY950255, AY950248, AY950241, AY950254, AY950267, AY950232, AY950253, AY950274, FJ534541, FJ534540, AY950281, AY950275, AY950268, AY950233, AY950282, AY950246, AY950239, AY950260, DQ064368, DQ064478, HM172434, HM172374, DQ064397, DQ064370, DQ064451, DQ064424, HM172127, HM172291, GU186679, HM172201, HM172340, HM172080, HM172236, DQ064505, DQ064558, DQ064531, DQ064504, DQ064476, DQ064449, DQ064422, DQ064395, DQ064477, DQ064559, DQ064532, DQ064396, DQ064369, DQ064450, DQ064423, HM172255, EF362426, EF362429, EF362428, EF362431, EF362422, EF362433, EF362432, GQ917235, GQ917231, GQ917236, GQ917234, EF362427, EF362430, GQ917233, EF362419, AB261854, AB261855, AB261856, AB261853, AB261850, AB261851, AB261852, EF362418, EF362421, EF362420, EF362423, AB261857, EF362425, EF362424, GQ917232, EF492391, EF492362, EF492239, EF492420, GQ120534, GQ148820, GQ148848, EF492422, EF492393, EF492364, DQ683039, EF492322, EF492293, EF492260, GQ148834, GU186742, EF473080, GU186740, GU186743, GQ917237, GQ917238, GU186744, GQ148862, GQ140277, GQ120561, GQ140263, EF473082, EF473079, GU186741, GU182139, GU182140, GU182141, HM172293, HM172233, HM172170, HM172141, GU182146, FJ784838, FJ784822, GU182145, GU182142, GU182143, GU182144, HM172112, FJ784815, FJ784799, FJ784847, FJ784831, HM172195, HM172150, HM172268, HM172427, HM172393, HM172353, FJ784863, FJ784783, FJ784767, FJ784879, FJ784806, GU182162, GU182161, HM145699, GU182160, GU182157, GU182158, GU182159, HM144685, HM144854, HM145023, HM145192, HM145530, HM145361, HM144515, GU182156, FJ784870, HM172428, HM172404, FJ784886, FJ784854, FJ784790, FJ784774, HM172130, HM172303, GU182155, HM172173, HM172319, HM172114, HM172224, AY648294, AY648293, AY648292, AY650274, AY650273, AY650272, AY650271, AY648291, AY648288, AY648287, AY648290, AY648289, AY650270, AY650276, AY653039, AY650275, HM006748, AY253753, AY253754, AY253755, AY743216, AY253750, AY253751, AY253752, FJ610134, FJ610137, FJ610136, FJ610139, AY253756, FJ610141, FJ610140, EU084931, HM006753, HM006754, HM006755, HM006752, HM006749, HM006750, HM006751, EU084930, EU182301, EU182302, AY240921, EU084927, EU084928, EU084929, FJ610135, FJ610138, FJ577552, FJ577551, FJ577554, FJ577553, FJ577556, FJ577555, FJ577550, HM346520, HM346519, HM346518, HM346517, HM346524, HM346523, HM346522, FJ577557, GU186603, GU186606, GQ122490, GU186604, GU186607, GU186602, GU186605, GQ122493, GQ122494, GQ122495, GQ122415, GQ122491, GQ122492, GQ122395, HM346521, FJ577534, FJ577537, FJ577536, FJ577539, HM346489, FJ577541, FJ577540, HM346506, HM346501, HM346504, HM346507, FJ577535, FJ577538, HM346508, HM346486, FJ577521, FJ577520, FJ577519, FJ577518, FJ577525, FJ577524, FJ577523, HM346485, HM346488, HM346487, HM346490, FJ577522, HM346492, HM346491, EU743000, EU742995, EU742998, EU743001, EU182276, EU182277, EU743002, EU182251, EU182252, EU182253, EU182250, EU742997, EU742996, EU742999, EU182275, AY611524, AY611527, AY611526, AY616764, GU186781, AY616766, AY616765, EU182272, EU182273, EU182274, EU182271, AY611525, AY611528, EU182270, EU182254, JN588932, JN588917, JN588903, JQ714239, JQ714236, JQ714237, JQ714238, JN588887, GU186609, GU186608, JN588836, JN588815, JN588871, JN588845, JQ714235, EU182259, EU182260, AF474035, EU182258, EU182255, EU182256, EU182257, JQ714232, JQ714233, JQ714234, AF474064, EU182261, AF474045, AF474055, HM346503, GQ122556, GQ122557, GQ122558, GQ122555, GQ122552, GQ122553, GQ122554, GQ122398, GQ122418, GQ122511, GQ122510, GQ122559, GQ122508, GQ122509, GQ122507, GQ122549, GQ122550, GQ122551, GQ122548, GQ122545, GQ122546, GQ122547, GQ122417, GQ122505, GQ122506, GQ122397, GQ122502, GQ122503, GQ122504, GQ122512, GQ122534, GQ122402, GQ122422, GQ122533, GQ122530, GQ122531, GQ122532, GQ122539, GQ122540, GQ122403, GQ122538, GQ122535, GQ122536, GQ122537, GQ122529, GQ122563, GQ122564, GQ122565, GQ122562, GQ122513, GQ122560, GQ122561, GQ122528, GQ122401, GQ122421, GQ122527, GQ122566, GQ122567, GQ122526, DQ485214, DQ485215, DQ485216, DQ485213, DQ485210, DQ485211, DQ485212, DQ485221, DQ485222, DQ485223, DQ485220, DQ485217, DQ485218, DQ485219, DQ485209, JF906205, JF906206, JF906207, JF906204, HM346502, HM346505, JF906203, DQ485206, DQ485207, DQ485208, DQ485205, JF906208, JF906209, JF906210, DQ485224, GQ122435, GQ122496, GQ122497, GQ122434, GQ122385, GQ122405, GQ122433, GQ122500, GQ122501, GQ122544, GQ122499, GQ122498, GQ122396, GQ122416, GQ122432, CY047466, CY047467, CY047468, DQ485228, DQ485225, DQ485226, DQ485227, CY047471, GQ122430, GQ122431, CY047470, FJ667188, CY047469, FJ667187, GU186765, GQ122466, GQ122467, GU186762, GU186761, GU186764, GU186763, GQ122471, GQ122470, GQ122472, GQ122469, GQ122468, GQ122391, GQ122411, GU186766, HM172430, HM172390, HM172320, HM172287, GU186768, GU186767, HM172123, HM172095, HM172237, HM172180, GQ122473, DQ064434, DQ064407, DQ064488, DQ064461, DQ064542, DQ064515, DQ064354, GU186780, GU186779, GU186778, GU186777, GU186784, GU186783, GU186782, DQ064543, GQ122477, GQ122476, GQ122514, GQ122475, GQ122474, GQ122392, GQ122412, GQ122517, GQ122518, GQ122519, GQ122419, GQ122515, GQ122516, GQ122399, EF681874, EF681875, EF681876, EF681873, EF681870, EF681871, EF681872, EF681877, EF681882, DQ376905, DQ376869, EF681881, EF681878, EF681879, EF681880, DQ376878, DQ376842, DQ376806, DQ376777, DQ376741, DQ376705, DQ376668, DQ376626, EF681867, EF681868, EF681869, DQ376770, DQ376734, DQ376698, DQ376662, DQ376833, AB507267, AB507268, DQ376871, AB507266, AB507263, AB507264, AB507265, DQ376835, DQ376655, DQ376763, DQ376872, DQ376691, DQ376799, DQ376619, DQ376727, DQ376797, DQ376891, DQ376855, DQ376689, DQ376653, DQ376761, DQ376725, DQ376819, DQ376783, AB507261, AB507262, DQ376678, DQ376639, DQ376747, DQ376711, DQ376824, DQ376644, DQ376752, DQ376860, DQ376659, DQ376768, DQ376896, DQ376716, DQ376801, DQ376621, DQ376729, DQ376837, DQ376679, DQ376788, DQ376873, DQ376642, DQ376750, DQ376714, DQ376822, DQ376774, DQ376894, DQ376858, DQ376674, DQ376624, DQ376732, DQ376696, DQ376803, DQ376786, DQ376876, DQ376840, DQ376693, DQ376848, DQ376812, DQ376631, DQ376884, DQ376703, DQ376666, DQ376775, DQ376740, DQ376849, DQ376813, DQ376632, DQ376885, DQ376704, DQ376667, DQ376776, DQ376825, DQ376645, DQ376753, DQ376861, DQ376657, DQ376765, DQ376897, DQ376717, DQ376811, DQ376630, DQ376739, DQ376847, DQ376680, DQ376789, DQ376883, FJ868020, FJ868021, FJ868014, FJ868019, EU669199, EU669201, EU669200, FJ868015, JQ794460, JQ794459, JQ794454, JQ794461, FJ868016, FJ868017, FJ868018, AY849787, AY849793, AY849790, AY849785, DQ376660, DQ376767, AY849783, AY849791, EU669197, EU497919, EU669198, EU669196, AY849792, AY849788, EU669195, JQ794457, EF205209, EF205202, EF205195, HM172282, HM172258, HM172202, HM172162, EF205160, FJ868030, FJ868031, FJ868032, EF205188, EF205181, EF205174, EF205167, JQ794468, JQ794467, JQ794462, JQ794469, JQ794456, JQ794455, JQ794458, JQ794463, HM172380, HM172328, HM172086, HM172426, JQ794465, JQ794464, JQ794466, DQ376744, DQ376708, DQ376676, DQ376636, DQ376888, DQ376852, DQ376816, DQ376780, DQ376745, DQ376709, DQ376677, DQ376637, DQ376889, DQ376853, DQ376817, DQ376692, DQ376656, DQ376764, DQ376728, DQ376836, DQ376800, DQ376620, DQ376887, DQ376707, DQ376675, DQ376779, DQ376743, DQ376851, DQ376815, DQ376635, DQ376781, DQ376622, DQ376730, DQ376694, DQ376802, DQ376793, DQ376874, DQ376838, DQ376658, DQ376623, DQ376731, DQ376695, DQ376804, DQ376766, DQ376875, DQ376839, DQ376736, DQ376701, DQ376664, DQ376628, DQ376880, DQ376844, DQ376808, DQ376772, DQ376757, DQ376722, DQ376686, DQ376649, DQ376902, DQ376865, DQ376829, DQ376669, DQ914813, DQ914814, DQ914815, DQ914811, HM172135, HM172297, DQ914812, DQ914816, DQ064489, DQ064380, DQ064435, DQ064516, DQ914817, DQ914818, DQ064569, DQ064378, DQ064459, DQ064432, DQ064513, HM172296, DQ064567, DQ064540, DQ064405, HM172113, HM172243, HM172172, HM172337, DQ064486, HM172440, HM172361, DQ064408, GU354079, GU354080, GU354081, GU354078, HM172167, HM172118, HM172261, GU354082, GQ122569, GQ122570, GQ122571, GQ122568, GU354083, GU354084, GU354085, DQ064514, DQ064379, DQ064460, DQ064541, DQ064381, DQ064462, DQ064568, DQ064433, HM172309, HM172069, HM172214, HM172357, DQ064406, DQ064487, HM172452, HQ326723, JQ904463, JQ904464, JQ904462, JQ904460, JQ904461, HQ326722, DQ064565, DQ064430, DQ064403, DQ064484, DQ064457, DQ064538, DQ064511, DQ064376, EF605598, EF605601, EU500857, EF605599, EF605602, EF605603, EF605600, EU500858, EU500863, EU500864, JQ904459, EU500862, EU500859, EU500860, EU500861, DQ064566, HM172244, HM172212, HM172139, HM172073, HM172418, HM172376, HM172327, HM172274, HM172235, HM172194, HM172164, HM172082, HM172444, HM172382, HM172316, DQ064431, DQ064404, DQ064485, DQ064458, DQ064539, DQ064512, DQ064377, HM172414, HM172191, HM172153, HM172283, HM172252, HM172363, HM172329, HM172093, DQ376760, DQ376724, DQ376688, DQ376652, DQ376904, DQ376868, DQ376832, DQ376796, DQ376749, DQ376713, DQ376671, DQ376641, DQ376893, DQ376857, DQ376821, DQ376712, DQ376670, DQ376784, DQ376748, DQ376856, DQ376820, DQ376640, DQ376899, DQ376719, DQ376683, DQ376791, DQ376755, DQ376863, DQ376827, DQ376647, DQ376785, DQ376638, DQ376746, DQ376710, DQ376818, DQ376792, DQ376890, DQ376854, DQ376673, DQ376633, DQ376738, DQ376702, DQ376810, DQ376782, DQ376882, DQ376846, DQ376742, DQ376706, DQ376672, DQ376634, DQ376886, DQ376850, DQ376814, DQ376778, DQ376756, DQ376720, DQ376684, DQ376648, DQ376900, DQ376864, DQ376828, FJ868029, GQ122478, GQ122479, FJ868028, FJ868025, FJ868026, FJ868027, GQ122480, GQ122483, GQ122484, GQ122485, GQ122482, GQ122393, GQ122413, GQ122481, GQ122520, GQ122521, GQ122522, GQ122575, GQ122572, GQ122573, GQ122574, GQ122400, FJ868022, FJ868023, FJ868024, GQ122525, GQ122420, GQ122523, GQ122524, GQ122486, AB450630, AB450646, EF205203, AB450607, AB450538, AB450561, AB450584, EF205196, EF205161, EF205182, DQ376892, EF205168, EF205189, EF205154, EF205175, GQ122489, AB450508, AB450524, GQ122488, GQ122394, GQ122414, GQ122487, AB450540, AB450648, AB450506, AB450522, AB450632, AB450565, AB450586, AB450611, JN869522, JN869523, JF715007, JN869521, FJ581434, FJ581432, FJ581433, JN869524, JN869525, JN869526, JN869527, AB256706, AB256707, AB256708, AB256705, AB256750, AB256703, AB256704, AB256709, AB256674, AB256675, AB256676, AB256673, AB256710, AB256671, AB256672, AB256723, AB256724, AB256725, AB256722, AB256719, AB256720, AB256721, AB256726, AB256747, AB256748, AB256749, AB256746, AB256743, AB256744, AB256745, AB256677, JQ356883, JQ356874, JQ356880, JQ356889, JQ356891, JQ356894, JQ356886, JQ356877, FJ581430, FJ581431, FJ581428, FJ581435, JQ356892, JQ356895, FJ581429, JQ356872, JQ356878, JQ356875, JQ356881, AB256678, JQ356884, JQ356887, JQ356890, JQ356873, JQ356879, JQ356876, JQ356882, JQ356893, JQ356885, JQ356888, AB256718, AB593455, AB593456, AB593457, AB593454, AY818148, AB593452, AB593453, AB593458, GU356583, GU356584, GU356585, GU356582, AB593459, GU356580, GU356581, EU930999, EU930998, EU930997, EU930996, EU931003, EU931002, EU931001, EU931000, AY818139, AY818142, AY818145, AY818136, AY818127, AY818130, AY818133, GU356586, GU272005, GU272006, GU272007, GU272004, GU083656, GU083657, GU272003, GU272008, GU083622, GU083618, GU083623, GU083621, GU272009, GU272010, GU083620, GU271998, GU271999, GU272000, GU271997, GU356587, GU271995, GU271996, GU272001, GU083653, GU083654, GU083655, GU083652, GU272002, GU083650, GU083651, DQ094259, AY770615, DQ138166, DQ094281, DQ099787, DQ099758, DQ099768, DQ138145, DQ094252, AY770609, EU930883, DQ094276, DQ099784, DQ099755, DQ099761, FJ868037, DQ138178, DQ138158, FJ868036, FJ868033, FJ868034, FJ868035, DQ099790, AY770621, DQ138173, DQ138152, DQ094265, DQ099760, DQ099774, DQ094287, EU930882, EU930950, EU930949, EU930952, EU930951, EU930954, EU930953, EU930948, EU930979, EU930974, EU930973, EU930976, EU930975, EU930978, EU930977, EU930972, EU930877, EU930880, EU930899, EU930878, EU930881, EU930876, EU930879, EU930898, EU930893, EU930896, EU930955, EU930894, EU930897, EU930892, EU930895, HM172192, HM172145, HM172299, HM172256, HM172392, HM172348, HM172106, HM172446, HM172198, HM172143, HM172278, HM172246, HM172397, HM172343, HM172103, HM172142, HM172305, HM172436, HM172204, HM172347, HM172111, HM172247, HM172398, HM172154, HM172302, HM172447, HM172199, HM172349, HM172099, HM172239, AB166859, AB256731, AB256732, AB256733, AB256730, AB256727, AB256728, AB256729, AB256734, AB256715, AB256716, AB256717, AB256714, AB256711, AB256712, AB256713, GU186713, AB166862, GU186708, AB166861, GU186715, AB166860, GU186714, GU186711, GU186709, GU186712, AB166866, AB166865, AB166863, AB166864, GU186710, HM172435, HM172399, HM172345, HM172267, HM172219, HM172208, HM172125, HM172102, HM172448, HM172396, HM172344, HM172276, HM172225, HM172176, HM172161, GQ122425, GQ122426, GQ122384, GQ122424, GU083619, GU083624, GU083625, GQ122404, HM172383, HM172351, HM172108, HM172420, GQ122427, GQ122429, GQ122428, HM172091, HM172289, HM172442, HM172395, HM172144, HM172193, HM172350, HM172301, HM172439, HM172400, HM172138, HM172094, HM172251, HM172184, HM172445, HM172386, HM172346, HM172295, HM172240, HM172207, HM172147, HM172090, HM172248, FJ384752, FJ384744, FJ384745, FJ384753, FJ384755, JN869528, JN869529, FJ384746, FJ384754, FJ384747, HM172475, HM172455, HM172467, DQ997185, HM172483, HM172479, HM172463, DQ064533, DQ064506, DQ064371, HM172459, HM172471, DQ064560, JN869532, JN869533, JN869534, JN869530, JF715006, JN869531, DQ997181, DQ997184, DQ997183, DQ997179, DQ997180, DQ997186, DQ997182, HM346499, HM346498, HM346493, FJ577527, FJ577530, HM346500, HM346496, AB256679, AB256680, AB256681, HM346495, HM346494, HM346497, HM346512, HM346511, HM346510, HM346515, HM346514, HM346509, HM346513, FJ577526, FJ577529, FJ577528, FJ577533, FJ577532, FJ577531, AY676035, AY676039, AY676043, AY676023, AY676027, AY676031, AY676047, HQ871938, HQ871933, HQ871937, AY676051, HQ871939, HQ871940, AB256685, AB256686, AB450503, AB256682, AB256683, AB256684, AB450519, AB450598, AB450627, AB450643, AB450535, AB450552, AB450581, HM346516, DQ997272, DQ997271, DQ997267, DQ997269, DQ997274, DQ997268, DQ997273, DQ997283, DQ997285, DQ997284, DQ997289, DQ997288, DQ997287, DQ064479, AY653193, AY653199, DQ064452, DQ064425, DQ064398, AY653198, AY653194, AY653197, DQ997270, AY653200, AY653196, AY653195, AB450626, AB450642, FJ577549, AB450550, AB450580, AB450596, FJ577548, FJ577544, FJ577543, FJ577546, FJ577547, FJ577542, FJ577545, DQ997545, DQ997551, DQ997547, DQ997290, DQ997286, DQ997546, DQ997549, AB450502, AB450518, AB450534, DQ997548, DQ997552, DQ997550, GQ117132, GQ117131, GQ117130, GQ117129, GQ117136, GQ117135, GQ117134, GQ117133, HM172241, HM172205, HM172136, HM172076, HM172443, HM172375, HM172336, AY340077, AY340091, AY342424, AY342427, AY340085, AY342420, AY338458, AB438938, AB438943, AB438944, AB438945, AB438942, AB438939, AB438940, AB438941, HM172285, AB188816, AB188817, AB188818, AB188815, GU186736, AB188813, AB188814, AB188819, EF205155, EF205176, EF205169, EF205190, AB188820, EF205204, EF205197, DQ064456, DQ064429, DQ064402, DQ064375, DQ064564, DQ064537, DQ064510, DQ064483, GU186735, GU186734, GU186733, GU186732, GU186739, GU186738, GU186737, AY342414, EU743028, EU743031, FJ517330, EU743029, EU743032, EU743027, EU743030, FJ517329, AY241604, AY241643, EU084903, AY254121, FJ517328, AY240895, FJ517327, EU743257, EU084909, EU084910, EU743254, EU743253, EU743256, EU743255, EU084911, AY241638, EU743034, EU743033, AY241599, AY240891, EU084912, AY254116, EU084904, GU354067, GU354068, GU354069, GU354066, GU354063, GU354064, GU354065, AB450505, AB450602, AB450629, AB450645, AB450583, AB450521, AB450537, AB450556, AY241606, AY241645, GU050388, AY254123, EU084905, AY240897, EU084906, GU050387, GU050382, GU050385, GU354062, GU050383, GU050386, GU050381, GU050384, EF205162, FM163437, FM163438, FM163439, AB450650, AB450588, AB450614, AB450634, FM163440, EU672455, EU672456, EU672457, FM163444, FM163441, FM163442, FM163443, CY015038, CY015037, CY015033, CY015039, EU735796, EU735795, EU735798, CY015035, AB450526, AB450542, AB450568, AB450510, AY207504, CY015034, CY015036, EU676174, EU401755, EU402405, EU402404, EU401752, EU401751, EU401754, EU401753, EU402403, EU402402, EF605604, EF605597, EU402399, EU402398, EU402401, EU402400, FJ610101, FJ610100, FJ610099, EU672459, EU672458, EU672460, EU676173, FJ610094, EU414265, EU420032, EU408333, FJ610098, FJ610097, FJ610096, FJ610095, EU735797, AB256689, AB256690, AB256691, AB256688, AB256669, AB256670, AB256687, AB256692, AB256697, AB256698, AB256699, AB256696, AB256693, AB256694, AB256695, AB256738, AB256739, AB256740, AB256737, EF205183, AB256735, AB256736, AB256741, AB256666, AB256667, AB256668, AB256665, AB256742, AB256663, AB256664, AB256700, EU735816, EU735815, EU735810, EU735817, EU742963, EU742962, EU742965, EU735813, EU735800, EU735799, EU735794, EU735801, EU735812, EU735811, EU735814, EU742971, AY240900, EU742970, EU742972, AB256701, AB256702, EU742973, EU742969, EU742966, EU742961, EU742964, EU742967, AY241609, AY241647, EU742968, HM172250, HM172077, HM172148, HM172196, HM172355, HM172265, HM172163, HM172362, HM172431, HM172308, GU186566, GU186567, GU186580, GU186569, GU186568, GU186571, GU186572, GU186565, GU186570, HM172478, HM172482, HM172453, HM172473, GU186720, GU186719, GU186716, GU186717, GU186718, HM172466, HM172078, HM172338, HM172206, HM172260, HM172371, HM172458, HM172461, HM172433, HM172469, GU186579, HM346477, HM346482, HM346479, HM346480, HM346483, FJ577511, FJ577512, HM346484, FJ577514, HM346478, AY254109, EU182292, AY241630, AY241592, AY240884, EU084913, HM346481, EU084915, EU084914, EF474443, GU186577, EF474445, EF474444, GU186574, GU186573, GU186578, GU186575, GU186576, EF474450, FJ577515, FJ577516, FJ577513, FJ577510, FJ577517, EF474448, EF474447, EF474446, EF474449, GU186721, EU662947, EU662957, EU662952, EU662946, EU662956, EU662960, EU662961, EU662959, EU662958, EU662953, EF205208, EU662955, EF205194, EF205201, EU662954, EU662951, EU662950, EU662949, EU662948, JN852795, JN852800, JN852797, JN852798, JN852801, HQ871934, HQ871935, JN852802, HQ871936, JN852796, JN852805, JN852806, JN852807, JN852804, JN852803, JN852810, JN852799, JN852808, JN852809, EF205159, DQ323673, DQ323677, DQ323679, DQ323678, DQ323672, DQ323675, DQ449636, DQ323674, DQ323676, AB188821, AB189049, AB189048, GU186722, GU186723, AB189047, AB188823, AB188822, AB189046, AB188824, EU163434, EU163435, EU163432, EU163431, EU163436, EF205173, EF205180, EF205187, EF205166, EU163433, DQ449635, DQ449632, DQ449633, DQ449634, DQ449637, EU163430, EU163429, DQ449638, DQ449639, EU742982, EU743024, EU743019, EU743022, AY241633, EU743093, EU743092, EU743260, EU743259, EU743258, EU743021, EU743087, EU743023, EU742979, AY254112, AY241595, EU742981, EU742980, EU742978, AY646080, AY646081, AY646082, AY646079, AY646078, AY646085, AY646083, AY646084

Mammal:

HQ292044, HQ291950, HQ291944, HQ291875, HQ292008, HQ291918, HQ291994, HQ291858, DQ508883, DQ508878, DQ508879, HQ291869, HQ292019, DQ508882, HQ291900, DQ508880, DQ508881, HQ291948, HQ291970, HQ291873, HQ292020, HQ291859, HQ291934, HQ291894, HQ292009, HQ291919, HQ292033, HQ291968, HQ291933, HQ291893, HQ291983, HQ291898, HQ291920, HQ291845, HQ291995, HQ292041, HQ291870, HQ291991, HQ291945, HQ292016, HQ291866, HQ291941, HQ291895, HQ291846, HQ291921, HQ291887, HQ291912, HQ291996, HQ291971, HQ291865, HQ292021, HQ291962, HQ291993, HQ291880, HQ292028, HQ291907, HQ291928, DQ508885, DQ508884, HQ291957, HQ291978, HQ292003, HQ291888, HQ291943, HQ291963, HQ292043, HQ291981, HQ291868, HQ291913, HQ292018, HQ291874, HQ292024, HQ291899, HQ291924, DQ415370, DQ415359, HQ291949, HQ291974, HQ291999, DQ508874, DQ508873, DQ508876, DQ508875, DQ508870, HQ291849, DQ508872, DQ508871, DQ415348, DQ415297, DQ415286, DQ415319, DQ415308, DQ415340, DQ415329, DQ415362, DQ415351, DQ415330, DQ415315, DQ415304, DQ415337, DQ415326, DQ415352, DQ415341, DQ415293, DQ415363, DQ508877, HQ291851, HQ292001, HQ291967, HQ291982, HQ291882, HQ292026, HQ291905, HQ291926, HQ292032, HQ291984, HQ291857, HQ292034, HQ291969, HQ291932, HQ291892, HQ292007, HQ291917, HQ291955, HQ291906, HQ291927, HQ291852, HQ292002, HQ291956, HQ291977, HQ291881, HQ292027, HQ291973, HQ291998, HQ291901, HQ291976, HQ291848, HQ292023, HQ291951, HQ291923, HQ291876, HQ292015, JQ714243, HQ291867, JQ714245, JQ714244, HQ291942, HQ291891, HQ292017, HQ291916, JQ714246, M26079, L11133, M73524, M25935, M63531, AY210191, AY210103, AY209932, HQ292042, HQ292006, HQ292004, HQ291972, HQ291856, HQ291884, HQ292029, HQ291909, HQ291929, HQ291965, HQ291847, HQ291997, HQ291966, HQ291992, HQ291890, HQ292022, HQ291915, HQ291922, EU434698, DQ508890, DQ508889, DQ508892, DQ508891, DQ508886, JQ714250, DQ508888, DQ508887, DQ508893, AY626147, AY555150, AY626149, AY626148, AY626144, AY626146, AY626145, AY555151, JQ714249, EU434700, EU434699, EU268222, EU268223, EU434696, EU434695, EU434694, EU434697, EU268221, EU434701, EU268216, JQ714248, JQ714247, EU268219, EU268220, EU268217, EU268218, HQ292011, HQ291861, HQ291936, HQ291903, HQ292039, HQ291871, HQ291989, HQ291946, HQ291878, HQ292000, HQ291850, HQ291925, HQ291904, HQ291853, HQ292036, HQ291986, HQ291953, HQ291939, HQ291990, HQ291961, HQ292013, HQ291863, HQ291940, HQ291911, HQ292040, HQ291886, HQ291897, HQ291864, HQ291988, HQ291896, HQ292014, HQ291872, HQ291938, HQ291947, HQ292038, HQ291879, HQ291931, HQ291889, HQ291987, HQ291914, HQ291854, HQ291985, HQ292031, HQ291964, HQ291958, HQ291862, HQ292012, HQ291959, HQ291979, HQ291883, HQ292037, HQ291908, HQ291937, HQ291952, HQ291910, HQ292005, HQ291885, HQ291930, HQ291954, HQ292025, HQ291855, HQ291975, HQ292030, HQ291935, HQ291902, HQ292035, HQ291877, HQ291980, HQ291960, HQ292010, HQ291860, DQ508856, DQ508857, DQ508854, DQ508855, DQ508860, DQ508861, DQ508858, DQ508859, HQ166048, HQ166042, HQ166043, AY342422, HQ166041, HQ166046, HQ166047, HQ166044, HQ166045, JN582058, JN582062, JN582061, JN582064, JN582063, EU399758, EU399757, JN582060, JN582059, JN582065, JN582055, JN582054, JN582057, JN582056, JN582051, JN582066, JN582053, JN582052, AY340089, HQ664920, HQ664921, HQ664915, HQ664916, HQ664930, HQ664931, HQ664929, HQ664937, HQ664914, HQ664926, HQ664927, HQ664924, HQ664925, HQ664936, HQ664918, HQ664928, HQ664917, HQ664932, AY342413, AY340083, HQ166055, HQ166056, AY342425, AY340079, AY342418, AY338459, HQ166054, HQ664935, HQ166049, HQ664933, HQ664934, HQ166052, HQ166053, HQ166050, HQ166051, AF389116, AF389117, J02146, AF389115, AF389120, AF389121, AF389118, AF389119, J02147, EF467822, EF467823, EF467821, V01088, EF467817, J02150, EF467824, V01099, AF389122, GQ457549, GQ457557, GQ457565, GQ457563, GQ457560, GQ457546, GQ457554, GQ457551, GQ457545, GQ457562, GQ457547, GQ457544, GQ457566, GQ457550, GQ457559, GQ457556, GQ457553, EF467820, DQ469960, DQ469961, DQ469962, DQ469959, DQ508862, DQ487334, DQ469958, V01106, DQ469957, EU399754, EU399753, EU399756, EU399751, DQ469955, DQ469956, EU399752, EU399755, DQ487333, DQ508869, DQ487336, DQ508868, DQ487338, J02151, EF467819, V00603, EF467818, DQ508867, DQ508864, DQ487340, DQ508863, DQ487335, DQ487339, DQ487337, DQ508865, DQ508866, DQ415333, DQ415322, DQ415355, DQ415344, DQ415289, DQ415369, DQ415311, DQ415300, DQ415366, DQ415345, DQ415334, DQ415367, DQ415356, DQ415301, DQ415290, DQ415323, DQ415312, DQ415358, DQ415305, DQ415294, DQ415327, DQ415316, HQ200597, HQ200598, DQ415283, HQ200600, DQ415338, DQ415325, DQ415314, DQ415347, DQ415336, DQ415360, DQ415349, DQ415303, DQ415292, DQ415284, DQ415291, DQ415365, DQ415313, DQ415302, DQ415332, DQ415321, DQ415354, DQ415343, DQ415324, DQ415296, DQ415285, DQ415318, DQ415307, DQ415346, DQ415335, DQ415368, DQ415357, DQ415310, DQ415350, DQ415339, DQ415287, DQ415361, DQ415306, DQ415295, DQ415328, DQ415317, DQ415298, DQ415364, DQ415353, DQ415299, DQ415288, DQ415320, DQ415309, DQ415342, DQ415331, HM172213, HM172072, HM172120, HM172165, HM172451, HM172266, HM172310, HM172359, HM172264, GQ457552, GQ457555, AB462292, GQ457561, GQ457564, GQ457567, GQ457558, GQ457548, HM172159, GU907119, GU907115, GU907114, GU907120, HQ664919, HQ664923, GU907116, HQ664922, GU907117, HM172104, HM172342, HM172189, HM172254, GU907121, GU907118, HM172394, HM172438, AB462293, AX399725, EF587281, AX399727, AX399726, EF587278, EF587277, EF587280, EF587279, AX399728, JN588893, HQ200601, HQ200599, HQ200596, AX399730, AX399729, JN588923, AX399731, EF587276, AB462299, AB462298, GQ850593, GQ850592, AB462295, AB462294, AB462297, AB462296, GQ850594, AX399724, GQ850599, EF587275, EF587274, GQ850596, GQ850595, GQ850598, GQ850597, DQ009921, DQ009922, DQ009920, DQ009917, DQ009923, EU268229, EU268228, EU268231, EU268230, DQ009918, DQ835314, DQ835313, DQ835316, DQ835315, DQ835312, DQ009924, DQ009919, DQ835311, DQ835310, HM114613, HM114612, DQ508903, DQ508902, HM114611, HM114608, HM114607, HM114610, HM114609, DQ508904, EU268225, EU268224, EU268227, EU268226, DQ508909, DQ508906, DQ508905, DQ508908, DQ508907, DQ835317, DQ508843, DQ508844, DQ508841, DQ508842, DQ508845, FJ445088, FJ445027, FJ445077, FJ445052, DQ508840, FJ445085, FJ445063, FJ445080, FJ445075, FJ445068, DQ508838, DQ508839, FJ445079, FJ445039, EU263984, EU263982, FJ445065, EU263983, EU263985, EU263987, EU263988, EU263981, EU263986, FJ445084, FJ445082, FJ445062, FJ445056, FJ445043, FJ445070, FJ445028, FJ445076, FJ445029, FJ445060, HM114587, HM114586, HM114589, HM114588, HM114585, HM114582, AY818147, HM114584, HM114583, HM114614, HM114621, HM114620, HM114543, HM114542, HM114619, HM114616, HM114615, HM114618, HM114617, AY818129, HM006756, HM006758, HM006757, AY818126, DQ508931, DQ508930, DQ508933, DQ508932, AY818132, AY818144, AY818141, HM006763, HM006762, HM006761, HM006759, AY818135, AY818138, HM006760, HM114544, HM114570, HM114569, HM114572, HM114571, HM114568, HM114565, HM114564, HM114567, HM114566, HM114573, HM114604, HM114603, HM114606, HM114605, HM114602, HM114599, HM114598, HM114601, HM114600, HM114551, HM114550, HM114553, HM114552, HM114549, HM114546, HM114545, HM114548, HM114547, HM114554, HM114561, HM114560, HM114563, HM114562, HM114559, HM114556, HM114555, HM114558, HM114557, AB212054, AB212055, AB212052, AB212053, AB212056, JQ070797, JN638726, AB212057, AB212059, AB212051, AJ404634, AJ404637, AF348198, AJ404630, AJ404626, AJ278646, AJ278649, AJ289871, AJ404629, JN638728, JQ070794, JQ070795, JN638729, JQ070789, JQ070790, JQ070788, JQ070777, JQ070782, JQ070799, JN638730, JQ070796, JN638731, JQ070798, JN638733, JN638727, JN638732, JQ070800, JQ070793, AF348188, HM172110, HM172315, HM172211, HM172257, HM172369, FJ445053, FJ445025, HM172413, FJ445030, HM172149, HM172298, EU597804, AF348170, EU285583, EU597805, AF348172, AF348180, AF348184, AF348174, AF348176, EU597803, FJ445069, FJ445037, FJ445046, FJ445090, FJ445036, EU597802, EU283414, EU597800, EU597801, FJ445049, FJ445051, FJ445055, FJ445067, FJ445058, FJ445035, FJ445034, FJ445042, FJ445073, FJ445078, DQ889688, DQ889685, DQ889686, DQ889687, FJ445086, FJ445074, FJ445047, FJ445048, FJ445066, FJ445050, FJ445044, FJ445054, FJ445087, FJ445072, FJ445061, FJ445091, FJ445083, FJ445033, FJ445064, FJ445045, FJ445031, FJ445071, FJ445057, FJ445041, FJ445026, FJ445038, FJ445089, FJ445032, DQ889689, JQ290174, JQ290178, JQ070786, JQ290173, JQ290180, JQ290177, JQ290176, JQ290175, JQ290179, JQ070779, JQ070792, JQ070778, JQ070781, JQ070784, JQ070785, JQ070780, JQ070787, JQ070783, JQ070791, JQ290181, JQ290187, JQ290184, JQ290188, JQ290183, DQ889683, DQ889684, JQ290182, DQ889682, JQ290186, JQ290168, JQ290172, JQ290169, JQ290170, JQ290165, JQ290166, JQ290185, JQ290171, JQ290167, GQ902804, GQ902803, GU183796, GQ902805, GQ902802, GQ902799, GQ902798, GQ902801, GQ902800, GU183797, GQ902814, GU183803, GQ902816, GQ902815, GU183802, GU183799, GU183798, GU183801, GU183800, GU271958, GU271957, GU271960, GU271959, GU271956, GU271985, GU271984, GU271955, GU271986, GU271961, GQ902795, GQ902794, GQ902797, GQ902796, GQ902793, GQ902790, GU271962, GQ902792, GQ902791, GQ902817, GU271952, GU271951, GU271954, GU271953, GU271950, GU271947, GU183811, GU271949, GU271948, GU271987, GU271994, GU271993, GQ902831, GQ902830, GU271992, GU271989, GU271988, GU271991, GU271990, GQ902824, GQ902823, GQ902826, GQ902825, GQ902822, GQ902819, GQ902818, GQ902821, GQ902820, GQ902827, GU183808, GU183807, GU183810, GU183809, GU183806, GQ902829, GQ902828, GU183805, GU183804, FJ912998, FJ912997, EU625363, FJ912999, DQ360842, DQ360840, DQ360835, DQ360841, DQ360836, FJ913000, EU625364, FJ913005, EU625367, FJ913006, FJ913004, FJ913001, EU625366, FJ913003, FJ913002, AY627893, AY555152, AY627892, AY627894, AY627895, AY627897, AY627898, AY555153, AY627896, AY627891, DQ360837, AY627888, DQ360839, DQ360838, AY627887, AY627885, AY627890, AY627886, AY627889, FJ913007, GU271971, GQ902813, GU271973, GU271972, GQ902812, GQ902809, GQ902808, GQ902811, GQ902810, GU271974, GU271981, GU271980, GU271983, GU271982, GU271979, GU271976, GU271975, GU271978, GU271977, EU625368, FJ913012, FJ913014, FJ913013, EU625365, FJ913009, FJ913008, FJ913011, FJ913010, GQ983545, GQ983552, GQ983551, GQ902807, GQ902806, GQ983550, GQ983547, GQ983546, GQ983549, GQ983548, FJ912925, FJ912924, FJ912927, FJ912926, FJ912923, EU021268, FJ912921, EU021269, FJ912922, EU021254, FJ912933, FJ912932, FJ912934, EU021260, FJ912931, EU021255, FJ912928, FJ912930, FJ912929, FJ912994, FJ912993, FJ912996, FJ912995, FJ912992, FJ912989, FJ912988, FJ912991, FJ912990, FJ912913, FJ912918, FJ912917, FJ912920, FJ912919, EU021259, FJ912915, FJ912914, FJ912916, EU021258, EU021261, EU434693, EU434692, DQ508895, DQ508894, EU434691, EU434688, EU434687, EU434686, EU434689, DQ508896, DQ508927, DQ508926, DQ508929, DQ508928, DQ508901, DQ508898, DQ508897, DQ508900, DQ508899, FJ912940, EU021252, FJ912941, EU021253, FJ912939, FJ912936, FJ912935, FJ912938, FJ912937, FJ912942, HQ533867, HQ533869, EU434690, HQ533873, HQ533870, HQ533876, HQ533878, HQ533865, HQ533875, EU021274, FJ912945, EU021275, FJ912946, FJ912944, GU271969, GU271968, FJ912943, GU271970, FJ912947, EU021283, FJ912952, FJ912954, FJ912953, EU021282, FJ912949, FJ912948, FJ912951, FJ912950, GU183812, GQ902837, GU183814, GU183813, GQ902836, GQ902833, GQ902832, GQ902835, GQ902834, GU183815, GU271965, GU271964, GU271967, GU271966, GU271963, GU183817, GU183816, GU183819, GU183818, FJ912961, FJ912975, FJ912974, FJ912977, FJ912976, FJ912973, EU021265, FJ912910, FJ912912, FJ912911, FJ912978, FJ912985, FJ912984, FJ912987, FJ912986, FJ912983, FJ912980, FJ912979, FJ912982, FJ912981, FJ912969, FJ912968, FJ912967, EU021277, FJ912970, EU021276, FJ912966, EU021272, FJ912963, FJ912962, FJ912965, EU021273, FJ912964, FJ912971, FJ912909, EU021264, FJ912908, FJ912972, FJ912907, JF714039, JF714040, JF714036, JF714035, JF714038, JF714037, DQ150427, DQ150426, DQ150429, DQ150428, DQ150423, DQ150422, DQ150425, DQ150424, JF714034, AY790306, AY790307, AY790308, AY790309, AB434383, AB434382, AB434381, AB434387, AB434388, JF714033, AB434386, AY790305, AB434384, AB434385, FJ611898, FJ611899, FJ611900, FJ611897, DQ469982, FJ611896, FJ611895, FJ410139, FJ410140, FJ410141, FJ410138, FJ611901, FJ611902, FJ410137, DQ469985, HM125983, HM125984, HM125985, HM125982, HM125979, HM125980, HM125981, DQ469986, DQ469983, DQ469984, DQ469981, HM125986, DQ469979, DQ469980, AY790310, JN409415, JN409416, JN409417, JN409414, JN409411, JN409412, JN409413, JN409422, JN409423, JN409424, JN409421, JN409418, JN409419, JN409420, JN409410, JN409400, JN409401, JN409402, JN409399, JN409396, JN409397, JN409398, JN409407, JN409408, JN409409, JN409406, JN409403, JN409404, JN409405, GQ484358, GQ484357, GQ484359, JN409443, JN409440, JN409441, JN409442, GQ484362, AY790311, AY790312, GQ484361, GQ484355, GQ484360, GQ484356, JN409439, JN409429, JN409430, JN409431, JN409428, JN409425, JN409426, JN409427, JN409436, JN409437, JN409438, JN409435, JN409432, JN409433, JN409434, EU258939, EU258936, EU258937, EU258940, JQ689096, EU258942, EU258941, EU258948, EU258943, EU258946, EU258949, EU258938, EU258935, EU258950, JQ689095, JF346138, FJ519967, JF346157, FJ519977, JF411839, EU692896, JF346158, JQ689092, JQ689093, JQ689094, JQ689091, JF346146, JQ689089, JQ689090, JQ689137, JQ689138, JQ689139, AB441174, AB441173, AB441172, AB441171, JQ689144, JN940419, JN940420, JQ689143, JQ689140, JQ689141, JQ689142, AB441170, JQ689114, JQ689115, JQ689116, JQ689113, EU258945, EU258944, EU258947, AB441177, AB441176, AB441175, JQ689120, JQ689117, JQ689118, JQ689119, JF346143, JF411838, HQ315644, JF346153, JF346142, JF346134, FJ519963, JF346127, JF346145, JF411840, HQ315645, JF346130, FJ519976, JF346137, FJ519966, FJ519974, EU692894, JF346152, HQ315643, JF411837, FJ410142, FJ410143, FJ410144, JF411835, EU692892, JF346150, JF346144, JF346136, FJ519965, JF346129, FJ519962, JF346133, JF346149, JF346141, EU692891, JF346156, FJ519973, JF346135, FJ519964, JF346128, FJ519975, JF411836, EU692893, JF346151, JF411842, JF346131, JF346147, JF411841, FJ519960, JF346154, FJ519971, JF346139, FJ519961, JF346132, JF346148, JF346140, EU692890, JF346155, FJ519972, JN409395, JN809156, JN809174, JN809200, JN809137, JN809219, JN809102, JN809120, JN809145, JN809157, JN809182, JN809128, JN809209, JN809228, JN809110, JN809218, JF820286, JF820287, JF820288, JF820285, JF820282, JF820283, JF820284, JN809147, JN809165, JN809183, JN809129, JF820289, JN809093, JN809111, JN809105, JN809123, JN809140, JN809230, JN809176, JN809193, JN809211, JN809231, JN809106, JN809124, JN809212, JN809160, JN809177, JN809194, JN809159, JN809121, JN809138, JN809158, JN809103, JN809198, JN809217, JN809236, JN809104, JN809122, JN809139, JN809229, JN809175, JN809192, JN809210, JF820281, GU135946, GU135947, GU135948, GU135945, GU135942, GU135943, GU135944, GU135953, GU135954, GU135955, GU135952, GU135949, GU135950, GU135951, GU135941, GU135931, GU135932, GU135933, GU135930, GU135927, GU135928, GU135929, GU135938, GU135939, GU135940, GU135937, GU135934, GU135935, GU135936, GU135975, GU135976, GU135977, GU135974, GU135971, GU135972, GU135973, JF820278, JF820279, JF820280, JF820277, JF820274, JF820275, JF820276, GU135970, GU135960, GU135961, GU135962, GU135959, GU135956, GU135957, GU135958, GU135967, GU135968, GU135969, GU135966, GU135963, GU135964, GU135965, JN809100, JN809118, JN809135, JN809225, JN809171, JN809189, JN809206, JN809226, JN809101, JN809119, JN809207, JN809154, JN809172, JN809190, JN809153, JN809116, JN809133, JN809152, JN809098, JN809186, JN809203, JN809222, JN809099, JN809117, JN809134, JN809224, JN809170, JN809188, JN809205, EU502902, EU502903, EU502904, EU502905, EU502907, EU502908, EU502901, JN409392, JN409393, JN409394, JN409391, JN409388, JN409389, JN409390, EU502906, JN809208, JN809227, EU502898, JN809191, JN809136, JN809155, JN809173, EU502894, EU502895, EU502896, EU502897, EU502899, EU502900, EU502893, JN809168, JN809196, JN809215, JN809234, JN809180, JN809126, JN809143, JN809163, JN809181, JN809197, JN809216, JN809164, JN809109, JN809127, JN809144, JN809108, JN809213, JN809232, JN809107, JN809199, JN809141, JN809161, JN809178, JN809195, JN809214, JN809233, JN809179, JN809125, JN809142, JN809162, JN809132, JN809150, JN809169, JN809115, JN809202, JN809221, JN809097, JN809114, JN809131, JN809151, JN809096, JN809187, JN809204, JN809223, JN809185, JN809148, JN809166, JN809184, JN809130, JN809235, JN809094, JN809112, JN809146, JN809149, JN809167, JN809113, JN809201, JN809220, JN809095, JN940421, AB620176, AB620177, AB620178, AB620175, AB620172, AB620173, AB620174, AB434336, AB434337, AB434338, AB434335, AB620179, AB434333, AB434334, AB620203, AB620193, AB620194, AB620195, AB620192, AB620189, AB620190, AB620191, AB620200, AB620201, AB620202, AB620199, AB620196, AB620197, AB620198, AY700214, AY700213, AY646424, AY700215, GU086059, GU086060, GU086061, GU086062, GU086063, GU086064, AY700216, AY646426, AY646425, AY700217, GU086058, AY619956, AY619961, AY619958, AY619955, AB434339, AB434340, AY619954, GU086055, GU086056, GU086057, GU086054, AY619960, AY619959, AY619957, AB620188, AB434374, AB434375, AB434376, AB434373, AB434298, AB434299, AB434300, AB600244, AB600562, AB600846, AB434380, AB434377, AB434378, AB434379, AB434297, AB434287, AB434288, AB434289, AB434286, EU826546, EU826547, AB434285, AB434294, AB434295, AB434296, AB434293, AB434290, AB434291, AB434292, AB600867, AB600946, AB620160, AB600862, AB600847, AB600852, AB600857, AB620165, AB620166, AB620167, AB620164, AB620161, AB620162, AB620163, AB600563, AB600945, AB434413, AB434414, AB600866, AB600851, AB600856, AB600861, AB434419, AB434420, AB600245, AB434418, AB434415, AB434416, AB434417, EU004446, EU004445, EU004444, EU004447, HM440141, HM440140, HM440143, HQ541647, HQ541646, HQ541645, EU004441, EU004443, EU004442, EU004440, HM440142, HQ840334, HQ840338, HQ840331, HQ840336, FJ789835, FJ789836, HQ840335, HM440145, HM440144, HM440139, HM440146, HQ840337, HQ840333, HQ840332, DQ139322, DQ139323, JF714024, DQ139321, DQ139326, DQ139320, DQ139324, JF714019, JF714018, JF714017, JF714020, JF714023, JF714022, JF714021, DQ139327, HQ541644, AB434349, AB434350, HQ541641, HQ541640, HQ541643, HQ541642, AB434355, AB434356, DQ139325, AB434354, AB434351, AB434352, AB434353, FJ789834, GU086076, GU086077, EU516309, GU086075, GU086072, GU086073, GU086074, EU516305, EU516306, EU516307, EU516308, EU516310, EU516311, EU516304, GU086071, GU086069, EU116038, EU116037, GU086068, GU086065, GU086066, GU086067, EU116043, EU116044, GU086070, EU116042, EU116039, EU116040, EU116041, EU502885, FJ789828, FJ789827, FJ789826, FJ374516, FJ789824, FJ789825, FJ789838, FJ789832, FJ789833, FJ789831, FJ789829, FJ789837, FJ789830, FJ374518, EU502886, EU502889, EU502892, EU502888, EU502890, EU502884, EU502887, FJ374511, FJ374515, FJ374514, FJ374517, EU502891, FJ374512, FJ374513, EU826544, EU604692, EU604693, EU604689, EU604691, HM440149, HM440148, HM440151, AB600242, AB600560, AB600844, EU604696, EU604694, EU604690, EU604695, HM440150, HM440104, HM440099, HM440102, HM440105, JN162040, JQ914806, HM440106, HM440153, HM440152, HM440147, HM440154, HM440101, HM440100, HM440103, DQ280244, DQ280240, DQ469987, DQ280242, DQ280239, DQ280243, DQ280241, DQ469992, DQ469993, DQ469990, DQ469991, DQ469988, DQ469989, DQ469994, DQ280238, AB600943, DQ280245, DQ280246, AB600864, AB600849, AB600854, AB600859, DQ280252, DQ280248, DQ280237, DQ280251, DQ280247, DQ280250, DQ280249, JN162021, AB434403, AB434404, AB600243, AB434402, AB434399, AB434400, AB434401, AB600860, AB600865, AB600944, AB600855, AB600561, AB600845, AB600850, AB434398, JN940426, AB434357, AB434358, JN940425, JN940422, JN940423, JN940424, AB434363, AB434364, AB434397, AB434362, AB434359, AB434360, AB434361, JQ624667, JQ624668, JQ624669, JQ624666, EF551057, JQ624664, JQ624665, JQ914804, JN162059, JQ914805, JQ914803, JQ624670, JQ624671, JQ914802, EF551056, EU924272, EU924273, EU478814, EU924271, EU924268, EU924269, EU924270, EF551053, EF551054, EF551055, EF551052, EU924274, EF551050, EF551051, HQ825238, HQ825239, HQ825240, HQ825237, HQ825234, HQ825235, HQ825236, HQ825245, HQ825246, HQ825247, HQ825244, HQ825241, HQ825242, HQ825243, HQ825233, JF316647, JF713871, JF713872, JF316646, JF316643, JF316644, JF316645, JF713877, JF713878, HQ825232, JF713876, JF713873, JF713874, JF713875, HQ825198, HQ825199, HQ825200, HQ825197, HQ825186, HQ825195, HQ825196, EU826548, EU826543, EU826545, EU826549, HQ825201, HQ825202, EU826550, HQ825185, HQ825191, HQ825192, HQ825193, HQ825190, HQ825187, HQ825188, HQ825189, HQ825182, HQ825183, HQ825184, HQ825181, HQ825194, HQ825179, HQ825180, JF316642, DQ280227, DQ280225, DQ280226, DQ280223, DQ280232, DQ280221, DQ280222, DQ280215, DQ280219, DQ280217, DQ280214, DQ280228, DQ280224, DQ280213, DQ280234, AY619974, AY619975, AY619976, AY619977, AY619970, AY619971, AY619972, DQ280236, DQ280233, DQ280235, DQ280231, AY619973, DQ280229, DQ280230, AY619962, AY619963, AY619964, DQ280192, DQ280193, DQ280194, DQ280196, AY619965, JF316640, JF316641, AY619967, AY619969, AY619968, AY619966, DQ280195, DQ280206, DQ280207, DQ280212, DQ280205, DQ280218, DQ280220, DQ280216, DQ280189, DQ280190, DQ280191, DQ280208, DQ280209, DQ280211, DQ280210, GU135926, GQ422386, GQ422385, GQ422388, GQ422387, GQ422384, GQ422408, GQ422390, GQ422383, GQ422382, GQ422397, GQ422403, GQ422409, GQ422391, GQ422435, GQ422427, GQ422389, GQ422415, GQ422421, HQ853342, HQ853341, HQ853343, HQ853339, HQ853340, HM189306, HM189305, HM189304, HM189307, GQ422437, GQ422414, GQ422396, GQ422402, GQ422420, HQ853345, HQ853344, GQ422426, HQ853346, GQ422428, GQ422411, GQ422419, GQ422425, GQ422394, GQ422433, GQ422417, GQ422399, GQ422405, GQ422424, GQ422429, GQ422434, GQ422418, GQ422412, GQ422406, GQ422436, GQ422392, GQ422398, GQ422395, GQ422401, GU086014, GQ422410, GQ422404, GQ422422, GQ422431, GQ422432, GQ422416, GU086021, GU086020, GQ422423, GQ422430, GU086019, GU086016, GU086015, GU086018, GU086017, HM189308, HM215159, HM215164, HM215161, HM215162, HM215165, HM223595, HM223596, HM215166, HM223598, HM215154, HM215151, HM215152, HM215153, HM215156, HM215163, HM215160, HM215157, HM215158, FJ830853, FJ830852, FJ830855, FJ830854, GQ161159, GQ161156, GQ161155, GQ161158, GQ161157, HM223599, HM223600, HM223597, HM223594, HM223601, FJ830857, FJ830856, FJ830859, FJ830858, HM215171, HM215168, HM210858, HM210859, HM215169, HM215172, HM215173, HM215170, HM215167, HM189301, HM210856, HM189303, HM189302, HM210853, HM210852, HM210857, HM210854, HM210855, HM223588, HM223589, HM223590, HM223587, HM223586, HM223593, HM215155, HM223591, HM223592, HM210861, HM210862, HM215174, HM210864, HM210863, HM210866, HM210867, HM210860, HM210865, GQ422407, JN375232, JN375197, JN375017, JN375269, JN375160, JN375057, JN375016, JN375129, JN375093, JN375270, JN375233, JN375059, JN375018, JN375198, JN375094, JN375058, JN375161, JN375130, JN375158, JN375127, JN375228, JN375194, JN375080, JN375265, JN375226, JN375050, JN375008, JN375195, JN375159, JN375267, JN375227, JN375128, JN375009, JN375266, JN375081, JN375051, JN375100, JN375061, JN375165, JN375134, JN375021, JN375201, JN375164, JN375273, JN375236, JN375135, JN375098, JN375203, JN375166, JN375063, JN375237, JN375202, JN375022, JN375274, JN375019, JN375271, JN375096, JN375060, JN375234, JN375131, JN375095, JN375199, JN375162, JN375062, JN375020, JN375133, JN375097, JN375272, JN375163, JN375132, JN375235, JN375200, JN375193, JN375187, JN375151, JN375259, JN375223, JN375120, JN375001, JN375258, JN375073, JN375043, JN375229, JN375188, JN375003, JN375260, JN375152, JN375044, JN375002, JN375121, JN375074, GU086081, GU086080, GU086083, GU086082, GU086079, GQ422393, GQ422400, GU086078, GQ422413, JN375150, JN375119, JN375222, JN375186, JN375072, GU086085, GU086084, JN375042, JN375000, JN375048, JN375006, JN375125, JN375078, JN375263, JN375155, JN375124, JN375224, JN375191, JN375079, JN375049, JN375157, JN375126, JN375007, JN375192, JN375156, JN375264, JN375225, JN375261, JN375230, JN375046, JN375004, JN375189, JN375075, JN375045, JN375153, JN375122, JN375005, JN375262, JN375077, JN375047, JN375231, JN375123, JN375076, JN375190, JN375154, AB598486, AB598480, AB598498, AB598492, HM440119, HM440118, HM440115, HM440116, HM440117, AB598493, AB598487, AB598505, AB598499, AB598481, AB598510, AB598504, AB598522, AB598516, HM440079, HM440076, HM440089, HM440090, HM440077, HM440080, HM440081, HM440078, HM440075, HM440122, HM440087, HM440120, HM440121, HM440084, HM440083, HM440088, HM440085, HM440086, AB598514, AB598508, AB598526, AB598520, AB598502, AB598484, AB598525, AB598496, AB598490, AB598521, AB598515, DQ469971, AB598527, AB598509, AB598491, AB598485, AB598503, AB598497, AB598500, AB598494, AB598512, AB598506, AB598488, AB598517, AB598511, AB598482, AB598523, AB598507, AB598501, AB598519, AB598513, AB598495, AB598524, AB598518, AB598489, AB598483, HM440082, FJ200417, DQ280200, FJ200419, FJ200418, DQ280204, DQ280203, DQ280199, DQ280202, DQ280201, AB600564, AB600246, AB600853, AB600848, FJ200422, FJ200420, FJ200415, FJ200421, FJ200416, AF251429, AF251433, DQ469963, AF251434, AF251427, AF251430, AF251432, AF251431, AF251428, DQ469966, DQ469969, DQ280198, DQ280197, DQ469968, DQ469965, DQ469964, DQ469967, DQ469970, HM440062, HM440059, HM440060, HM440061, HM440064, HM440055, HM440052, HM440065, HM440066, HM440069, HM440070, HM440071, HM440068, HM440067, HM440074, HM440063, HM440072, HM440073, GQ161103, GQ161102, GQ161105, GQ161104, GQ161101, AB600863, AB600858, AB600947, AB600868, HM440051, HM440056, HM440053, HM440054, HM440057, GQ161107, GQ161106, HM440058, GQ161108, DQ469972, HM440131, HM440136, HM440133, HM440134, HM440137, HM440124, HM440125, HM440138, HM440127, AB434409, AB434408, AB434411, AB434410, AB434407, HM440135, HM440132, AB434406, AB434405, HM440113, HM440114, HM440107, HM440112, HM440095, HM440094, HM440091, HM440092, HM440093, HM440128, HM440129, HM440126, HM440123, HM440130, HM440109, HM440110, HM440111, HM440108, FJ536814, FJ536811, AY747623, AY747624, FJ536812, FJ536815, FJ536817, FJ536813, FJ536810, EU478823, AY747621, GQ161154, GQ161153, AY747620, AY747617, AY747622, AY747618, AY747619, JF290393, JF290392, JF290395, JF290394, JF290391, JF290388, AB434412, JF290390, JF290389, AY747612, AY747610, FJ536816, AY747613, AY747611, AY747615, AY747616, AY747609, AY747614, HM440096, FJ200427, FJ200426, FJ200428, FJ200423, FJ200425, AB434330, AB434329, AB434332, AB434331, AB434320, AB434319, AB434322, AB434321, AB434318, FJ200429, FJ200424, AB434317, FJ200430, AB434341, DQ469974, AB434343, AB434342, DQ469977, DQ469978, DQ469973, DQ469976, DQ469975, AB434326, AB434325, AB434328, AB434327, AB434348, AB434345, AB434344, AB434347, AB434346, EU053144, EU053143, EU053130, EU053145, EU053142, EU053139, EU053138, EU053141, EU053140, EU053137, EU053136, HM440097, HM440098, EU053135, EU053132, EU053131, EU053134, EU053133, AB434305, AB434304, AB434307, AB434306, AB434303, AB434324, AB434323, AB434302, AB434301, AB434314, AB434313, AB434316, AB434315, AB434312, AB434309, AB434308, AB434311, AB434310, JQ365118, JQ365117, JQ365119, JQ689160, JQ365116, JQ738181, JQ738180, JQ738183, JQ738182, JQ689165, JQ365123, JQ689166, JQ365124, JQ365122, JQ689162, JQ689161, JQ365121, JQ365120, JQ738168, JQ738151, JQ738170, JQ738169, JQ738150, JQ738147, JQ738146, JQ738149, JQ738148, JQ738177, JQ738176, JQ738179, JQ738178, JQ738175, JQ738172, JQ738171, JQ738174, JQ738173, JQ350559, JQ689164, JQ914812, JQ350560, JQ350558, JQ350556, JQ350555, JQ689163, JQ350557, JQ914816, JN652475, JQ914818, JQ914817, JN652445, JQ914814, JQ914813, JQ914815, JN652415, JQ350546, JQ689153, JQ350547, JQ689154, JQ350545, JQ365125, JQ689167, JQ350544, JQ350543, JQ350554, JQ350553, JQ689156, JQ689155, JQ350552, JQ350549, JQ350548, JQ350551, JQ350550, JQ738145, GQ229324, GQ229329, GQ229325, GQ229328, GQ229315, GQ229318, GQ229317, GQ229319, GQ229322, JQ220539, JQ220536, JQ220546, JQ220543, JQ220533, GQ229330, GQ229326, GQ229323, GQ229327, JN375317, JN375312, JQ220537, JQ220534, JN375307, JN375292, JN375287, JN375302, JN375297, GQ229321, JQ220556, GQ229320, GQ229316, JQ220553, JQ220544, JQ220540, JQ220550, JQ220547, JQ738157, JQ738156, JQ738159, JQ738158, JQ738155, JQ738152, DQ150437, JQ738154, JQ738153, JQ738166, JQ738165, JQ738144, JQ738167, JQ738164, JQ738161, JQ738160, JQ738163, JQ738162, GQ229301, GQ229304, GQ229306, GQ229302, GQ229300, JQ220552, JQ220549, GQ229305, JQ220555, DQ150434, DQ150433, DQ150436, DQ150435, DQ150432, GQ229299, GQ229303, DQ150431, DQ150430, JQ914819, JQ350523, JQ350522, JQ350525, JQ350524, JQ350521, JQ689152, JQ689151, JQ350520, JQ350519, JQ350532, JQ350531, JQ350534, JQ350533, JQ350530, JQ350527, JQ350526, JQ350529, JQ350528, JQ738186, JQ738185, JQ738187, JQ398772, JQ738184, JF833357, JQ914785, JQ914786, JF833369, JQ689148, JQ689147, JQ689150, JQ689149, JQ689146, JQ398748, JQ398759, JQ689145, JQ738188, GU135915, GU135914, GU135917, GU135916, GU135913, GU135910, GU135909, GU135912, GU135911, GU135924, GU135923, EU422988, GU135925, GU135922, GU135919, GU135918, GU135921, GU135920, JQ350541, JQ350540, GU135896, JQ350542, JQ350539, JQ350536, JQ350535, JQ350538, JQ350537, GU135902, GU135901, GU135908, GU135907, GU135900, GU135898, GU135897, GU135899, EU422987, JF833345, JQ689124, JQ689123, JQ689126, JQ689125, JQ689122, JF812321, JF812298, JQ689121, JQ914791, JN656962, JF812276, JF812322, JF812299, JN656961, JQ689128, JQ689127, JN656960, JN656959, JQ914808, JQ914807, JN652492, JQ914809, JQ914821, JQ914820, JN652491, JN652557, JN652522, JQ914789, JQ914788, JQ914790, JF812275, JQ914787, JN652523, JQ914810, JQ914811, JN652558, JQ914801, JF812339, JQ914793, JQ914792, JF812316, JQ914799, JQ914798, JQ914800, JF812293, JQ914782, JQ914796, JQ914784, JQ914783, JF833358, JF833334, JQ914794, JF833346, JQ914795, JF812300, JN656967, JN656968, JF812323, JF812277, JN656964, JN656963, JN656966, JN656965, JQ689135, JQ689134, JQ914797, JQ689136, JQ689133, JQ689130, JQ689129, JQ689132, JQ689131, GU086033, GU086032, GU086035, GU086034, GU086031, GU086140, GU086139, GU086030, GU086141, JN375169, JN375114, JN375241, JN375205, JN375088, GU086037, GU086036, JN375052, JN375025, GU086138, GU086006, GU086029, GU086008, GU086007, GU086028, GU086025, GU086024, GU086027, GU086026, GU086135, GU086134, GU086137, GU086136, GU086013, GU086010, GU086009, GU086012, GU086011, JN375277, JN375055, JN375028, JN375117, JN375091, GU086045, GU086042, GU086041, GU086044, GU086043, GU086048, GU086047, GU086050, GU086049, GU086046, JN375208, JN375171, JN375280, JN375244, GU086040, JN375242, JN375206, JN375027, JN375278, JN375170, JN375053, JN375026, JN375115, JN375089, JN375279, JN375243, GU086039, GU086038, JN375207, JN375090, JN375054, JN375172, JN375116, GU086023, EU273791, EU273779, EU273800, EU273804, EU273783, EU273795, EU273799, EU273787, EU273775, EU273805, EU273792, EU273797, EU273801, EU273780, EU273776, EU273796, EU273784, EU273788, EU273803, JN375167, JN375136, JN375239, JN375204, JN375099, JN375275, JN375238, JN375064, JN375023, JN375196, JN375168, JN375268, JN375240, JN375137, JN375024, JN375276, JN375101, JN375065, EU273777, JN375310, JN375305, JN375320, JN375315, JN375300, JN375285, JN375319, JN375295, JN375290, JN375316, JN375311, GU086022, JN375321, JN375306, JN375291, JN375286, JN375301, JN375296, JN375314, JN375293, JN375288, JN375303, JN375298, JN375283, EU273785, EU273789, EU273793, EU273781, JN375299, JN375294, JN375309, JN375304, JN375289, JN375313, JN375308, JN375284, JN375318, GU086051, EU273798, EU273802, EU273774, EU273794, HQ541666, HQ541667, HQ541668, HQ541665, EU273786, JQ277164, JQ277165, JQ277162, JQ277159, EU273778, EU273782, JQ277166, EU273790, HQ541664, EU086326, EU086321, GQ161117, GQ161116, EU086318, EU086328, EU086322, EU086324, GQ161118, HQ541671, GQ161123, HQ541669, HQ541670, GQ161120, GQ161119, GQ161122, GQ161121, JQ277161, GQ229378, GQ229374, GQ229371, GQ229375, GQ229372, GQ229377, GQ229373, GQ229376, GQ229337, GQ229335, GQ229338, JN375282, GQ229331, GQ229336, GQ229332, GQ229334, GQ229333, AB434396, DQ997253, DQ997257, DQ997254, DQ997255, JQ277163, JQ277160, DQ997251, DQ997252, DQ997258, AB434393, AB434392, AB434395, AB434394, AB434389, DQ997256, AB434391, AB434390, EU086331, FJ157993, FJ157992, EU015992, EU015993, FJ157987, FJ157990, FJ157989, FJ157991, FJ157986, EF612749, EU015988, EF612747, EF612748, EU015989, EF556199, EU015991, EF556200, EU015990, FJ157988, JN375173, JN375118, JN375245, JN375209, JN375092, GU086053, GU086052, JN375056, JN375029, EU516313, EU516316, EU516315, EU516314, EU516312, EU516317, JN375281, EU516319, EU516318, EF612742, JQ220554, EU086332, EU086330, JQ220551, JQ220542, JQ220545, JQ220548, EU086320, EU086327, EU086333, EU086323, EU086329, EU086319, EU086325, JQ220538, EU086302, EU086313, EU086309, EU086304, EU086306, EU086314, EF612746, EF612743, EF612744, EU086316, EF612745, JQ220535, JQ220532, EU086310, AB591844, AB591843, AB591848, EU794541, EU794540, DQ222917, AB591849, DQ222915, DQ222913, DQ222918, DQ222914, DQ222920, DQ222919, DQ222916, EU794533, EU794532, EU794535, EU794534, FJ375224, FJ375216, FJ375209, FJ375236, AB591846, AB591847, AB591842, AB591845, EU794537, EU794536, EU794539, EU794538, EU794557, EU794558, EU794559, EU794556, EU794553, EU794554, EU794555, EU794564, EU794569, EU794570, EU794571, EU794568, EU794565, EU794566, EU794567, EU794546, EU794547, EU794548, EU794545, EU794542, EU794543, EU794544, EU794549, EU794562, EU794563, EU794552, EU794561, EU794550, EU794551, EU794560, EU794511, EU794512, EU794513, EU794510, JQ228394, EU794508, EU794509, EU794514, GU571144, GU571146, GU571145, GU571149, EU794515, GU571147, GU571148, EU794496, EU794497, EU794498, EU794495, EU794492, EU794493, EU794494, EU794499, JQ228393, JQ228391, JQ228392, JQ228390, JQ228396, JQ228395, JQ228397, EU794517, EU794518, EU794519, EU794516, AY855343, AY855344, AY855345, EU794520, FJ375233, FJ375228, FJ375213, FJ375221, EU794521, EU794522, EU794523, EU794526, EU794527, EU794528, EU794525, GU571150, GU571151, EU794524, EU794529, AY855340, AY855341, AY855342, AY855339, EU794530, EU794531, AY855338, EU794578, EU794579, EU794576, EU794577, EU794573, EU794575, EU794572, EU794574, GU433362, GU433366, JN247576, GU433363, GU433364, GU433361, GU433365, JN247583, JN247582, JN247584, JN247586, JN247585, JN247578, JN247577, JN247579, JN247581, JN247580, GU433367, GU433376, GU433352, GU433346, GU433369, GU433374, GU433375, GU433349, GU433351, GU433350, GU433347, GU433348, GU433345, GU433372, GU433356, GU433357, GU433353, GU433368, GU433354, GU433355, GU433373, GU433370, GU433371, GU433358, GU433359, GU433360, JN247615, JN247616, JN247614, JN247612, JN247613, JN247617, JN247621, JN247622, JN247620, JN247618, JN247619, JN247611, JN247603, JN247604, JN247602, JN247600, JN247601, JN247605, JN247609, JN247610, JN247608, JN247606, JN247607, JN247623, JN247594, JN247593, JN247595, JN247597, JN247596, JN247592, JN247588, JN247587, JN247589, JN247591, JN247590, JN247598, EU127500, FJ560887, FJ560888, FJ560890, FJ560889, EU127501, JN247599, FJ560886, FJ560885, HQ454985, HQ454984, EU420048, EU420049, EU420046, EU420047, AM744955, AM744956, EU420050, EU420039, EU420040, EU420041, EU420042, EU420051, EU420052, EU420053, EU532424, EU532425, EU532426, EU532423, EU532420, EU532421, EU532422, AM744952, AM492165, AM744954, AM492166, EU532427, AM744953, AM744957, GQ176138, GQ176137, EU826555, GQ176139, GQ176142, GQ176141, GQ176144, EU826556, EU826557, EU826558, EU826551, EU826552, EU826553, EU826554, EF178522, EF178523, EF178524, EU420045, EU420038, EU420043, EU420044, EF178527, GQ176140, GQ176143, EF178526, AY834279, EF178525, AY834280, HQ454981, DQ643980, DQ643979, HQ454982, HQ316192, HQ454980, DQ643984, DQ643985, DQ643986, DQ643981, DQ643982, DQ643983, HQ454983, HQ316191
