# Supplementary material for: Comparison of CpG- and UpA-mediated restriction of RNA virus replication in mammalian and avian cells and investigation of potential ZAP-mediated shaping of host transcriptome compositions
Source: RNA. 2022 Aug;28(8):1089–109. doi: 10.1261/rna.079102.122 (PMC9297844; doi:10.1261/rna.079102.122)
Supplement: Supplemental Material [file supp_079102.122_Supplemental_Material_.zip › Supplemental_Table_S15.docx]

TABLE S15

IAV SEROTYPES ANALYSED FOR DINUCLEOTIDE COMPOSITION

|  | **Avian** | | | **Mammal** | | | |
| --- | --- | --- | --- | --- | --- | --- | --- |
| **Serotype** | **Chicken** | **Duck** | **All** | **Human** | **Swine** | **Other** | **All** |
| Avian H1 | 0 | 96 | 96 | 0 | 0 | 0 | 0 |
| Avian H2 | 8 | 176 | 184 | 0 | 0 | 0 | 0 |
| Avian H3 | 8 | 152 | 160 | 0 | 0 | 0 | 0 |
| Avian H4 | 0 | 72 | 72 | 0 | 0 | 0 | 0 |
| Avian H5^1^ | 104 | 496 | 600 | 0 | 0 | 0 | 0 |
| Avian H6 | 312 | 1336 | 1648 | 0 | 0 | 0 | 0 |
| Avian H7 | 296 | 88 | 384 | 0 | 0 | 0 | 0 |
| Avian H8 | 0 | 16 | 16 | 0 | 0 | 0 | 0 |
| Avian H9 | 784 | 64 | 848 | 0 | 0 | 0 | 0 |
| Avian H10 | 8 | 40 | 48 | 0 | 0 | 0 | 0 |
| Avian H11 | 0 | 104 | 104 | 0 | 0 | 0 | 0 |
| Avian H12 | 0 | 24 | 24 | 0 | 0 | 0 | 0 |
| Avian H13 | 0 | 16 | 16 | 0 | 0 | 0 | 0 |
| H1N1 | 8 | 216 | 224 | 528 | 787 | 0 | 1315 |
| H3N2 | 0 | 40 | 40 | 376 | 749 | 104 | 1229 |
| H5N1 | 1128 | 825 | 1953 | 218 | 139 | 48 | 405 |

^1^Non-H5N1 serotypes
