# Supplementary material for: Comparison of CpG- and UpA-mediated restriction of RNA virus replication in mammalian and avian cells and investigation of potential ZAP-mediated shaping of host transcriptome compositions
Source: RNA. 2022 Aug;28(8):1089–109. doi: 10.1261/rna.079102.122 (PMC9297844; doi:10.1261/rna.079102.122)
Supplement: Supplemental Material [file supp_079102.122_Supplemental_Material_.zip › Supplemental_Table_S16.docx]

TABLE S16

COMPARISON OF CODON NORMALISED COMPOSITIONS

OF IAV STRAINS INFECTING DIFFERENT HOSTS

A) Comparison of compositions of different segments of IAV strains infecting mammals and birds

|  | **CpG O/E** | | | | **UpA O/E** | | | |
| --- | --- | --- | --- | --- | --- | --- | --- | --- |
| **Segment** | **Avian** | **Mamm.** | **Diff.** | ***p*** | **Avian** | **Mamm.** | **Diff.** | ***p*** |
| 1 | 0.3719 | 0.3432 | 2.87% | <1E-30 | 0.7395 | 0.7354 | 0.41% | 1.6E-01 |
| 2 | 0.3579 | 0.3176 | 4.03% | <1E-30 | 0.7372 | 0.7687 | -3.15% | <1E-30 |
| 3 | 0.4411 | 0.3859 | 5.52% | <1E-30 | 0.6511 | 0.6843 | -3.32% | <1E-30 |
| 4 | 0.3318 | 0.3245 | 0.73% | <1E-30 | 0.7829 | 0.7658 | 1.71% | 2.0E-07 |
| 5 | 0.3468 | 0.3625 | -1.57% | 1.8E-13 | 0.5692 | 0.6267 | -5.75% | <1E-30 |
| 6 | 0.3076 | 0.3434 | -3.58% | <1E-30 | 0.8031 | 0.8025 | 0.06% | 0.24 |
| 7 | 0.4476 | 0.4348 | 1.28% | 7.8E-05 | 0.7971 | 0.81 | -1.29% | 0.01 |
| 8 | 0.4227 | 0.4827 | -6.00% | <1E-30 | 0.6119 | 0.6767 | -6.48% | <1E-30 |
|  |  |  |  |  |  |  |  |  |
| All | 0.3784 | 0.3743 | 0.41% | 1.5E-05 | 0.7115 | 0.7338 | -2.23% | <1E-30 |

B) Comparison of compositions of different segments of IAV strains infecting ducks and chickens

|  | **CpG O/E** | | | | **UpA O/E** | | | |
| --- | --- | --- | --- | --- | --- | --- | --- | --- |
| **Segment** | **Duck** | **Chicken** | **Diff.** | ***p*** | **Duck** | **Chicken** | **Diff.** | ***p*** |
| 1 | 0.37 | 0.3746 | -0.46% | <1E-30 | 0.7332 | 0.7485 | -1.53% | 1.0E-06 |
| 2 | 0.3494 | 0.3699 | -2.05% | 1.10E-13 | 0.7281 | 0.75 | -2.19% | 6.4E-12 |
| 3 | 0.4417 | 0.4402 | 0.15% | 0.60 | 0.6531 | 0.6482 | 0.49% | <1E-30 |
| 4 | 0.331 | 0.333 | -0.20% | 0.20 | 0.7731 | 0.7968 | -2.37% | 4.2E-09 |
| 5 | 0.3449 | 0.3496 | -0.47% | <1E-30 | 0.5472 | 0.6003 | -5.31% | <1E-30 |
| 6 | 0.3095 | 0.3048 | 0.47% | 0.20 | 0.8129 | 0.7893 | 2.36% | 1.6E-09 |
| 7 | 0.4428 | 0.4543 | -1.15% | 1.60E-06 | 0.7751 | 0.8281 | -5.30% | <1E-30 |
| 8 | 0.4161 | 0.432 | -1.59% | 1.50E-05 | 0.6045 | 0.6224 | -1.79% | 7.9E-06 |
|  |  |  |  |  |  |  |  |  |
| All | 0.3757 | 0.3823 | -0.66% | 8.20E-06 | 0.7034 | 0.723 | -1.96% | 4.8E-13 |

Footnotes as in Table S12

B) Ducks and chickens

|  | **G+C composition** | | | | **CpG O/E** | | | | **UpA O/E** | | | |
| --- | --- | --- | --- | --- | --- | --- | --- | --- | --- | --- | --- | --- |
| **Segment** | **Duck** | **Chicken** | **Diff.** | ***p*** | **Duck** | **Chicken** | **Diff.** | ***p*** | **Duck** | **Chicken** | **Diff.** | ***p*** |
| 1 | 44.56% | 44.62% | -0.06% | 0.111 | 0.4633 | 0.4677 | -0.44% | 0.1 | 0.6058 | 0.618 | -1.22% | 3.8E-06 |
| 2 | 43.37% | 43.24% | 0.13% | 5.5E-04 | 0.4306 | 0.4546 | -2.40% | 1.6E-13 | 0.5723 | 0.5936 | -2.13% | <1E-30 |
| 3 | 44.14% | 43.97% | 0.17% | 1.5E-07 | 0.5157 | 0.5164 | -0.07% | 0.50 | 0.4818 | 0.4782 | 0.36% | <1E-30 |
| 4 | 41.88% | 41.62% | 0.26% | 1.0E-03 | 0.387 | 0.3821 | 0.49% | 0.1 | 0.6472 | 0.681 | -3.38% | <1E-30 |
| 5 | 47.42% | 47.40% | 0.02% | 0.741 | 0.4644 | 0.4726 | -0.82% | 1.9E-04 | 0.4107 | 0.4542 | -4.35% | <1E-30 |
| 6 | 43.24% | 43.29% | -0.05% | 0.146 | 0.3485 | 0.3364 | 1.21% | 2.2E-04 | 0.7131 | 0.6868 | 2.63% | 1.3E-09 |
| 7 | 50.62% | 49.79% | 0.83% | <1E-30 | 0.4884 | 0.4973 | -0.89% | 4.9E-05 | 0.5909 | 0.6514 | -6.05% | <1E-30 |
| 8 | 46.09% | 45.89% | 0.20% | 3.5E-04 | 0.4922 | 0.5133 | -2.11% | 5.3E-06 | 0.4446 | 0.4613 | -1.67% | 1.9E-06 |
|  |  |  |  |  |  |  |  |  |  |  |  |  |
| All | 45.16% | 44.98% | 0.18% | 0.053 | 0.4487 | 0.4551 | -0.64% | 3.4E-06 | 0.5583 | 0.5781 | -1.98% | 2.7E-15 |
