# Supplementary material for: Comparison of CpG- and UpA-mediated restriction of RNA virus replication in mammalian and avian cells and investigation of potential ZAP-mediated shaping of host transcriptome compositions
Source: RNA. 2022 Aug;28(8):1089–109. doi: 10.1261/rna.079102.122 (PMC9297844; doi:10.1261/rna.079102.122)
Supplement: Supplemental Material [file supp_079102.122_Supplemental_Material_.zip › Supplemental_Table_S2.docx]

TABLE S2

SEQUENCES OF PRIMERS USED TO AMPLIFY AND CLONE IAV A SEGMENT 4 MUTANTS

| **Primer** | **Sequence 5’ - 3’** |
| --- | --- |
| S4 CGH insert F | AGAATGTGGCAGTGACACATTCGGTTAAC |
| S4 CGH insert R | ATCGCCAGAATCTGATACACGCCCATCGAT |
| S4 CGH vector F | ATCGATGGGCGTGTATCAGATTCTGGCGAT |
| S4 CGH vector R | GTTAACCGAATGTGTCACTGCCACATTCT |
