# Supplementary material for: Comparison of CpG- and UpA-mediated restriction of RNA virus replication in mammalian and avian cells and investigation of potential ZAP-mediated shaping of host transcriptome compositions
Source: RNA. 2022 Aug;28(8):1089–109. doi: 10.1261/rna.079102.122 (PMC9297844; doi:10.1261/rna.079102.122)
Supplement: Supplemental Material [file supp_079102.122_Supplemental_Material_.zip › Supplemental_Table_S3.docx]

TABLE S3

SEQUENCES OF PRIMERS USED FOR qPCR

| **Target** | **Sequence** |
| --- | --- |
| *All species* |  |
| HPRT1 | HPRT_F: TCAACCTTGACTGGAAAGAATG |
|  | HPRT_R: CAACAAATTTGTCTGGCACTTC |
| *Chicken* |  |
| IFN-β | chIFNb_F: GCCTCCTCAACCAGATCCAG |
|  | chIFNb_R: CATGGTCCCAGGTACAAGCA |
| ZAP | chZAP_F: TGCACCACCTTCAGAAGTGC |
|  | chZAP_R: CCTTCAACTTTACTTCTGGTGCC |
| *Duck* |  |
| IFN-β | duIFNb_F: ATCAACGCGCACTTTTTCCC |
|  | duIFNb_R: AGTGGTTGAGCTGGTTGAGG |
| ZAP | duZAP_F: CTTCCTTCCTGATGTCTGCC |
|  | duZAP_R: AGTGAGTGTTCATCCAAGAGC |
| *Quail* |  |
| IFN-β | quIFNb_F: GTGCCTCTTCCATTTCCGGA |
|  | quIFNb_R: CTTGTTGAGGAGGCTGTGGT |
| ZAP | quZAP_F: GTGCAACTTTCAGAAGATCCCAG |
|  | quZAP_R: AAGTGTTGGCAGAGGAATCATC |
| *Zebra finch* |  |
| IFN-β | zfIFNb_F: CTCTGGACACACGACGACTC |
|  | zfIFNb_R: TGGAAGAGGTGCTGGAGGAT |
| ZAP | zfZAP_F: TGGGATACAAGGCAGTGGTG |
|  | zfZAP_R: GCCACTGAAACACCTTCCAG |
