# Supplementary material for: Comparison of CpG- and UpA-mediated restriction of RNA virus replication in mammalian and avian cells and investigation of potential ZAP-mediated shaping of host transcriptome compositions
Source: RNA. 2022 Aug;28(8):1089–109. doi: 10.1261/rna.079102.122 (PMC9297844; doi:10.1261/rna.079102.122)
Supplement: Supplemental Material [file supp_079102.122_Supplemental_Material_.zip › Supplemental_Table_S4.docx]

TABLE S4

A) Sources of ZAP gene (*ZC3HAV1*) sequences used for PAML and diversity analysis

**Name Species Accession number**

Superb fairywren *Malurus cyaneus* ENSMCSG00000013325

Oriental scops-owl *Otus sunia* ENSOSUG00000007153

Little spotted kiwi *Apteryx owenii*  ENSAOWG00000014114

Spoon-billed sandpiper *Calidris pygmaea*  ENSCPGG00000011903

Golden eagle *Aquila chrysaetos* ENSACCG00020008429

Budgerigar *Melopsittacus undulatus* ENSMUNG00000015562

Kakapo *Strigops habroptilus*  ENSSHBG00005005804

Duck *Anas platyrhynchos* ENSAPLG00000011338

Japanese quail *Coturnix japonica* ENSCJPG00005016194

Ring-necked pheasant *Phasianus colchicus*  ENSPCLG00000011371

Turkey *Meleagris gallopavo* ENSMGAG00000013843

Chicken *Gallus gallus* ENSGALG00000013911

Helmeted guineafowl *Numida meleagris* ENSNMEG00000015515

B) Numbering used for sites^1^ referred to in the text.

**Model Align’t Chicken**

M2a 24 24

M2a 29 29

M2a 62 71

M2a 67 76

M2a 87 96

M2a 145 154

M2a 161 170

M2a 168 177

M2a 178 187

M2a 393 460

M2a 445 512

M2a 449 516

M2a 489 556

M2a 527 594

M2a 565 632

M2a 598 665

M8 24 24

M8 29 29

M8 38 38

M8 62 71

M8 67 76

M8 87 96

M8 143 152

M8 145 154

M8 147 156

M8 161 170

M8 168 177

M8 178 187

M8 213 281

M8 386 453

M8 393 460

M8 407 474

M8 445 512

M8 449 516

M8 489 556

M8 527 594

M8 565 632

M8 573 640

M8 591 658

M8 598 665

M8 601 668

M8 615 682

M8 645 712

^1^ This table can be used to translate the reported positively selected site from PAML site analyses M2a and M8 to the original chicken ZAP gene sequence. The alignment used for PAML removed a region of no homology between the fourth and fifth zinc-finger motifs.
