# Supplementary material for: Comparison of CpG- and UpA-mediated restriction of RNA virus replication in mammalian and avian cells and investigation of potential ZAP-mediated shaping of host transcriptome compositions
Source: RNA. 2022 Aug;28(8):1089–109. doi: 10.1261/rna.079102.122 (PMC9297844; doi:10.1261/rna.079102.122)
Supplement: Supplemental Material [file supp_079102.122_Supplemental_Material_.zip › Supplemental_Table_S5.docx]

TABLE S5

Sites under selection

| **Model comparison** | **2ΔlnL** | **p-value** | **dN/dS** | **% sites** | **Selected sites (Post. prob. > *0.95; **0.99)** |
| --- | --- | --- | --- | --- | --- |
| M1a/M2a | 104.07 | 0.00 | 2.89 | 12.07 | 24R*, 29L*, 62A**, 67D*, 87P*, 145M**, 161K*, 168Q*, 178S*, 393Q*, 445S*, 449A*, 489S**, 527V*, 565T**, 598S* |
|  |  |  |  |  |  |
| M7/M8 | 129.15 | 0.00 | 2.54 | 15.83 | 24R**, 29L**, 38R*, 62A**, 67D**,  87P**, 143D*, 145M**, 147R*, 161K*, 168Q**, 178S**, 213V*, 386P*, 393Q*, 407V*, 445S**, 449A**, 489S**, 527V*, 565T**, 573G*, 591P*, 598S**, 601A*, 615L*, 645I* |
|  |  |  |  |  |  |
| M8a/M8 | 104.19 | 0.00 |  |  |  |
