# Supplementary material for: Comparison of CpG- and UpA-mediated restriction of RNA virus replication in mammalian and avian cells and investigation of potential ZAP-mediated shaping of host transcriptome compositions
Source: RNA. 2022 Aug;28(8):1089–109. doi: 10.1261/rna.079102.122 (PMC9297844; doi:10.1261/rna.079102.122)
Supplement: Supplemental Material [file supp_079102.122_Supplemental_Material_.zip › Supplemental_Table_S6.docx]

TABLE S6

COMPARISON OF LINEAR REGRESSIONS OF G+C CONTENT WITH CpG AND UpA REPRESENTATION

**Genes Host Host m c R^2^ *p* t *p***

mRNA CpG Human 1.26 -0.23 0.38 <10^-100^  159 <10^-100^

Chicken 1.53 -0.33 0.48 <10^-100^ 5.5 3 x 10^-8^

mRNA UpA Human -0.74 0.91 0.25 <10^-100^  -107 <10^-100^

Chicken -0.83 0.95 0.25 <10^-100^  3.5 4 x 10^-4^

mRNA CpG Chicken 1.53 -0.33 0.48 <10^-100^  213 <10^-100^

Duck 1.53 -0.30 0.50 <10^-100^ 4.8 2 x 10^-6^

mRNA UpA Chicken -0.83 0.95 0.25 <10^-100^  -0.13 <10^-100^

Duck -0.85 0.97 0.28 <10^-100^  -0.86 1 x 10^-17^
