# Supplementary material for: Comparison of CpG- and UpA-mediated restriction of RNA virus replication in mammalian and avian cells and investigation of potential ZAP-mediated shaping of host transcriptome compositions
Source: RNA. 2022 Aug;28(8):1089–109. doi: 10.1261/rna.079102.122 (PMC9297844; doi:10.1261/rna.079102.122)
Supplement: Supplemental Material [file supp_079102.122_Supplemental_Material_.zip › Supplemental_Table_S7.docx]

TABLE S7

COMPARISON OF LINEAR REGRESSIONS OF G+C CONTENT WITH CpG AND UpA REPRESENTATION

A) Comparison of ISGs and mRNA sequences

**Host Motif Gene set m c R^2^ *p* t *p***

Human CpG mRNA 1.25 -0.22 0.39 <10^-100^  146

ISG 1.48 -0.33 0.43 7 x 10^-56^ -1.23 0.22

UpA mRNA -0.74 0.91 0.25 <10^-100^  -105

ISG -0.62 0.84 0.17 3 x 10^-19^ 2.27 **0.023**

Chicken CpG mRNA 1.53 -0.33 0.48 <10^-100^  210

ISG 1.60 -0.33 0.49 6 x 10^-35^ 0.23 0.82

UpA mRNA -0.83 0.95 0.25 <10^-100^  -125

ISG -0.80 0.95 0.25 8 x 10^-15^ -1.79 0.86

Duck CpG mRNA 1.53 -0.30 0.50 <10^-100^  193

ISG 1.77 -0.40 0.60 9 x 10^-44^ 0.67 0.51

UpA mRNA -0.85 0.97 0.28 <10^-100^  -122

ISG -0.77 0.94 0.28 2 x 10^-16^ -0.69 0.49

B) Comparison of mammalian and avian RNA virus gene sequences

**Motif Virus host m c R^2^ *p* t *p***

CpG Mammal 1.99 -0.41 0.41 <10^-100^  8.2

Avian 1.26 -0.10 0.20 1 x 10^-14^ 2.2 0.03

UpA Mammal -0.85 1.10 0.11 1 x 10^-41^  -5.9

Avian -1.11 1.25 0.15 3 x 10^-11^ 0.65 0.52
