# Supplementary material for: Comparison of CpG- and UpA-mediated restriction of RNA virus replication in mammalian and avian cells and investigation of potential ZAP-mediated shaping of host transcriptome compositions
Source: RNA. 2022 Aug;28(8):1089–109. doi: 10.1261/rna.079102.122 (PMC9297844; doi:10.1261/rna.079102.122)
Supplement: Supplemental Material [file supp_079102.122_Supplemental_Material_.zip › Supplemental_Table_S8.docx]

TABLE S8

IDENTIFIED HOMOLOGUES OF HUMAN ISGs IN THE CHICKEN GENOME

ABLIM3 ENSGALT00000105933.1

ABTB2 ENSGALT00000102854.1

ACSL1 ENSGALT00000088260.2

ADAR ENSGALT00000091771.1

AHNAK2 ENSGALT00000108049.1

AKT3 ENSGALT00000078278.2

ALDH1A1 ENSGALT00000024442.6

ALYREF ENSGALT00000011710.6

AMPH ENSGALT00000059785.2

ANGPTL1 ENSGALT00000099925.1

ANKFY1 ENSGALT00000002223.7

ANKRD22 ENSGALT00000103344.1

AQP9 ENSGALT00000064987.2

ARG2 ENSGALT00000015501.6

ARHGEF3 ENSGALT00000104431.1

ARNTL ENSGALT00000008634.5

ATF3 ENSGALT00000108231.1

ATL1 ENSGALT00000089842.2

ATP10D ENSGALT00000022955.5

ATP4A ENSGALT00000100969.1

B4GALT5 ENSGALT00000013071.6

BAG1 ENSGALT00000021488.6

BCL2L14 ENSGALT00000018829.7

BCL6 ENSGALT00000098470.1

BLVRA ENSGALT00000104657.1

BUB1 ENSGALT00000013400.6

C1S ENSGALT00000023545.5

C3AR1 ENSGALT00000021594.5

CASP7 ENSGALT00000014519.5

CCDC92 ENSGALT00000046182.3

CCNA1 ENSGALT00000027549.6

CCND3 ENSGALT00000005513.4

CD163 ENSGALT00000099854.1

CD38 ENSGALT00000083561.2

CD74 ENSGALT00000088722.2

CD9 ENSGALT00000030043.5

CDK17 ENSGALT00000018679.6

CDK18 ENSGALT00000100275.1

CDKN1A ENSGALT00000097804.1

CEBPD ENSGALT00000066024.2

CEL ENSGALT00000056488.2

CGAS ENSGALT00000025670.6

CH25H ENSGALT00000010268.6

CHMP5 ENSGALT00000021491.5

CMPK2 ENSGALT00000043751.3

CMTR1 ENSGALT00000016499.6

COMMD3 ENSGALT00000012825.5

CPT1A ENSGALT00000011466.6

CREB3L3 ENSGALT00000001900.6

CRY1 ENSGALT00000020627.6

CSRNP1 ENSGALT00000044280.4

CSRP1 ENSGALT00000000424.4

CXCR4 ENSGALT00000020184.4

CYP1B1 ENSGALT00000047969.2

CYTH1 ENSGALT00000067931.2

DCP1A ENSGALT00000054403.2

DDIT4 ENSGALT00000080768.2

DDX60 ENSGALT00000015699.6

DHX58 ENSGALT00000005325.5

DTX3L ENSGALT00000019727.6

DUSP5 ENSGALT00000071147.2

EGR1 ENSGALT00000012431.6

EHD4 ENSGALT00000014555.5

EIF2AK2 ENSGALT00000017183.2

EIF3L ENSGALT00000020091.4

ELF1 ENSGALT00000027391.4

EPAS1 ENSGALT00000046856.3

ERLIN1 ENSGALT00000005241.5

ETV6 ENSGALT00000037297.5

ETV7 ENSGALT00000054845.2

EXOC3L1 ENSGALT00000002614.6

EXT1 ENSGALT00000103176.1

FKBP5 ENSGALT00000090967.1

FLT1 ENSGALT00000027608.5

FNDC3B ENSGALT00000015009.6

FNDC4 ENSGALT00000026632.5

FOS ENSGALT00000043488.3

FOSB ENSGALT00000052563.2

FUT4 ENSGALT00000015959.6

FZD4 ENSGALT00000027873.4

GALNT2 ENSGALT00000018092.5

GBA3 ENSGALT00000079179.2

GCH1 ENSGALT00000019922.5

GCK ENSGALT00000051467.2

GEM ENSGALT00000061235.2

GJA4 ENSGALT00000003990.5

GLIPR2 ENSGALT00000043005.3

GMNN ENSGALT00000043851.3

GMPR ENSGALT00000042938.3

GPATCH11 ENSGALT00000064824.3

GTPBP1 ENSGALT00000059122.2

GTPBP2 ENSGALT00000016793.6

HEG1 ENSGALT00000019286.6

HELZ2 ENSGALT00000104315.1

HES4 ENSGALT00000085574.2

HESX1 ENSGALT00000088215.2

HIF1A ENSGALT00000019364.5

HIP1R ENSGALT00000053590.2

HIVEP2 ENSGALT00000106963.1

HK2 ENSGALT00000108207.1

HOOK2 ENSGALT00000107393.1

HOXD10 ENSGALT00000038688.4

HOXD11 ENSGALT00000059654.2

HPSE ENSGALT00000018267.6

IFI30 ENSGALT00000054220.2

IFI35 ENSGALT00000004470.6

IFIH1 ENSGALT00000053962.2

IFNGR1 ENSGALT00000031776.5

IFNLR1 ENSGALT00000098112.1

IGF2BP1 ENSGALT00000080686.2

IGFBP2 ENSGALT00000018698.5

IL15 ENSGALT00000038637.4

IL1R1 ENSGALT00000079162.2

IL6 ENSGALT00000097616.1

IL6ST ENSGALT00000023728.7

IMPA2 ENSGALT00000022420.6

IRF1 ENSGALT00000094481.1

IRF2 ENSGALT00000075597.3

IRF7 ENSGALT00000039023.4

ISG20 ENSGALT00000095215.1

JADE2 ENSGALT00000010368.7

JAK2 ENSGALT00000107987.1

JUN ENSGALT00000038039.4

KCNA5 ENSGALT00000106833.1

KCNJ15 ENSGALT00000025868.6

KIF2A ENSGALT00000060109.2

LAP3 ENSGALT00000028108.6

LEPR ENSGALT00000032933.2

LGALS3 ENSGALT00000063008.3

LGMN ENSGALT00000090059.2

LIPA ENSGALT00000010304.6

LMO2 ENSGALT00000067141.2

LPCAT1 ENSGALT00000106688.1

LRRN3 ENSGALT00000015449.4

MAFB ENSGALT00000005816.7

MAFF ENSGALT00000093539.1

MAGI2 ENSGALT00000091933.1

MAP2 ENSGALT00000106529.1

MAP3K14 ENSGALT00000040744.3

MAP3K5 ENSGALT00000055814.2

MARCKS ENSGALT00000047609.2

MASTL ENSGALT00000012133.6

MAX ENSGALT00000065978.2

MCL1 ENSGALT00000089951.2

MCOLN2 ENSGALT00000060976.2

MCUB ENSGALT00000019912.6

METAP2 ENSGALT00000018560.6

MKX ENSGALT00000012017.6

MTHFD2L ENSGALT00000017642.6

MVB12B ENSGALT00000102072.1

MYD88 ENSGALT00000044477.3

MYO1C ENSGALT00000062318.2

NAMPT ENSGALT00000013144.5

NAPA ENSGALT00000062438.2

NAPSA ENSGALT00000106219.1

NCALD ENSGALT00000079087.2

NCF1 ENSGALT00000035150.4

NCOA3 ENSGALT00000107895.1

NDC80 ENSGALT00000023897.4

NEURL1B ENSGALT00000104619.1

NFIL3 ENSGALT00000024541.4

NFKBIZ ENSGALT00000024766.4

NLRC5 ENSGALT00000058183.2

NMI ENSGALT00000088545.2

NOCT ENSGALT00000053885.2

NOS2 ENSGALT00000072375.3

NPAS2 ENSGALT00000096115.1

NR2F1 ENSGALT00000033927.4

NT5C3A ENSGALT00000078633.3

NUP50 ENSGALT00000058132.2

OASL ENSGALT00000029324.5

ODC1 ENSGALT00000026527.7

OGFR ENSGALT00000092445.1

OPTN ENSGALT00000022327.7

P2RY6 ENSGALT00000100424.1

PABPC4 ENSGALT00000006028.6

PADI2 ENSGALT00000055517.2

PARP12 ENSGALT00000087175.2

PCDH17 ENSGALT00000027383.6

PDGFRL ENSGALT00000032107.5

PDK1 ENSGALT00000021613.5

PDPK1 ENSGALT00000010359.3

PFKFB3 ENSGALT00000083042.2

PHF11 ENSGALT00000030139.5

PI4K2B ENSGALT00000057770.2

PIM3 ENSGALT00000039200.4

PLIN2 ENSGALT00000024341.6

PLSCR1 ENSGALT00000107339.1

PMM2 ENSGALT00000029998.5

PMP22 ENSGALT00000048380.2

PNPT1 ENSGALT00000102404.1

PNRC1 ENSGALT00000025447.3

PPM1K ENSGALT00000018386.5

PTGDR ENSGALT00000050232.2

PTX3 ENSGALT00000045711.3

PUS1 ENSGALT00000003714.7

PXK ENSGALT00000101401.1

RAB27A ENSGALT00000106709.1

RASGEF1B ENSGALT00000099085.1

RETREG1 ENSGALT00000091894.1

RGS1 ENSGALT00000034033.5

RIN2 ENSGALT00000013781.6

RIPK2 ENSGALT00000056899.2

RNASEH2A ENSGALT00000094036.1

RND1 ENSGALT00000058000.2

RNF114 ENSGALT00000013014.4

RNF19B ENSGALT00000102671.1

RNF213 ENSGALT00000049077.2

RSAD2 ENSGALT00000026450.5

RTCB ENSGALT00000020590.6

SAMD4A ENSGALT00000063249.2

SAMHD1 ENSGALT00000001868.5

SCARB2 ENSGALT00000018738.6

SCO2 ENSGALT00000091305.1

SDCBP2 ENSGALT00000045292.3

SEMA3D ENSGALT00000095428.1

SERPING1 ENSGALT00000011936.5

SLC15A2 ENSGALT00000019075.5

SLC16A1 ENSGALT00000002634.4

SLC16A12 ENSGALT00000010362.6

SLC1A1 ENSGALT00000016558.6

SLC25A21 ENSGALT00000087963.2

SLC25A28 ENSGALT00000012088.5

SLC25A30 ENSGALT00000027432.6

SMAD3 ENSGALT00000074963.2

SOCS1 ENSGALT00000011595.4

SOCS2 ENSGALT00000098204.1

SPATS2L ENSGALT00000087748.2

SPSB1 ENSGALT00000095416.1

SPTLC2 ENSGALT00000051622.2

SQLE ENSGALT00000074439.2

SSBP3 ENSGALT00000070782.2

SSTR2 ENSGALT00000038508.4

ST3GAL4 ENSGALT00000093416.1

STAP1 ENSGALT00000019102.5

STARD5 ENSGALT00000034132.5

STAT1 ENSGALT00000057169.2

STAT3 ENSGALT00000005170.7

STEAP4 ENSGALT00000105504.1

SUN2 ENSGALT00000043362.3

TAGAP ENSGALT00000101242.1

TBX3 ENSGALT00000047256.2

TCF7L2 ENSGALT00000014436.7

TDRD7 ENSGALT00000020176.7

TENT5A ENSGALT00000055761.2

TFEC ENSGALT00000068913.3

THEMIS2 ENSGALT00000043845.3

TLK2 ENSGALT00000101445.1

TLR3 ENSGALT00000021952.6

TLR7 ENSGALT00000102083.1

TMEM140 ENSGALT00000090133.2

TMEM255A ENSGALT00000014012.7

TMEM268 ENSGALT00000046863.2

TMEM51 ENSGALT00000073020.2

TNFAIP3 ENSGALT00000062647.2

TNFAIP6 ENSGALT00000020391.5

TNFSF10 ENSGALT00000089668.2

TRAF1 ENSGALT00000002404.7

TRAFD1 ENSGALT00000076176.2

TRANK1 ENSGALT00000095134.1

TRIM14 ENSGALT00000024720.6

TRIM25 ENSGALT00000049075.3

TXNIP ENSGALT00000095286.1

TYRP1 ENSGALT00000038332.4

UNC93B1 ENSGALT00000051466.3

UPP2 ENSGALT00000085587.2

VEGFC ENSGALT00000017629.5

VMP1 ENSGALT00000099277.1

WHAMM ENSGALT00000009658.6

XAF1 ENSGALT00000009537.7

YBX3 ENSGALT00000072603.3

ZNF385B ENSGALT00000092643.1
