# Supplementary material for: Comparison of CpG- and UpA-mediated restriction of RNA virus replication in mammalian and avian cells and investigation of potential ZAP-mediated shaping of host transcriptome compositions
Source: RNA. 2022 Aug;28(8):1089–109. doi: 10.1261/rna.079102.122 (PMC9297844; doi:10.1261/rna.079102.122)
Supplement: Supplemental Material [file supp_079102.122_Supplemental_Material_.zip › Supplemental_Table_S9.docx]

TABLE S9

INTERFERON α, β and γ GENES ANALYSED FROM DIFFERENT MAMMALIAN SPECIES

**Gene Gene symbol_Species**

IFN-α ENSOCUP00000002829_Rabbit_Oryctolagus_cuniculus

IFN-α ENSOCUP00000017093_Rabbit_Oryctolagus_cuniculus

IFN-α ENSOCUP00000017385_Rabbit_Oryctolagus_cuniculus

IFN-α ENSOCUP00000002178_Rabbit_Oryctolagus_cuniculus

IFN-α ENSOCUP00000018285_Rabbit_Oryctolagus_cuniculus

IFN-α ENSOCUP00000013451_Rabbit_Oryctolagus_cuniculus

IFN-α ENSPVAP00000002966_Megabat_Pteropus_vampyrus

IFN-α ENSPVAP00000002964_Megabat_Pteropus_vampyrus

IFN-α ENSPVAP00000013711_Megabat_Pteropus_vampyrus

IFN-α ENSRFEP00010011178_Greater_horseshoe_bat_Rhinolophus_ferrumequinum

IFN-α ENSRFEP00010011333_Greater_horseshoe_bat_Rhinolophus_ferrumequinum

IFN-α ENSTSYP00000015160_Tarsier_Carlito_syrichta

IFN-α ENSCJAP00000066482_Marmoset_Callithrix_jacchus

IFN-α ENSCJAP00000056423_Marmoset_Callithrix_jacchus

IFN-α ENSANAP00000027125_Ma's_night_monkey_Aotus_nancymaae

IFN-α ENSPSMP00000019518_Greater_bamboo_lemur_Prolemur_simus

IFN-α ENSPSMP00000019504_Greater_bamboo_lemur_Prolemur_simus

IFN-α ENSPSMP00000019537_Greater_bamboo_lemur_Prolemur_simus

IFN-α ENSPSMP00000003338_Greater_bamboo_lemur_Prolemur_simus

IFN-α ENSPSMP00000002376_Greater_bamboo_lemur_Prolemur_simus

IFN-α ENSPSMP00000019464_Greater_bamboo_lemur_Prolemur_simus

IFN-α ENSPSMP00000001877_Greater_bamboo_lemur_Prolemur_simus

IFN-α ENSPSMP00000001923_Greater_bamboo_lemur_Prolemur_simus

IFN-α ENSPSMP00000019495_Greater_bamboo_lemur_Prolemur_simus

IFN-α ENSPSMP00000001887_Greater_bamboo_lemur_Prolemur_simus

IFN-α ENSOGAP00000019053_Bushbaby_Otolemur_garnettii

IFN-α ENSOGAP00000016591_Bushbaby_Otolemur_garnettii

IFN-α ENSOGAP00000016507_Bushbaby_Otolemur_garnettii

IFN-α ENSOGAP00000021825_Bushbaby_Otolemur_garnettii

IFN-α ENSOGAP00000017882_Bushbaby_Otolemur_garnettii

IFN-α ENSGGOP00000035522_Gorilla_Gorilla_gorilla_gorilla

IFN-α ENSP00000276927

IFN-α ENSPPAP00000016834_Bonobo_Pan_paniscus

IFN-α ENSPPAP00000001899_Bonobo_Pan_paniscus

IFN-α ENSPTRP00000080901_Chimpanzee_Pan_troglodytes

IFN-α ENSPTRP00000089453_Chimpanzee_Pan_troglodytes

IFN-α ENSNLEP00000026765_Gibbon_Nomascus_leucogenys

IFN-α ENSNLEP00000022839_Gibbon_Nomascus_leucogenys

IFN-α ENSPTEP00000012443_Ugandan_red_Colobus_Piliocolobus_tephrosceles

IFN-α ENSCANP00000000912_Angola_colobus_Colobus_angolensis_palliatus

IFN-α ENSMLEP00000012226_Drill_Mandrillus_leucophaeus

IFN-α ENSPANP00000035694_Olive_baboon_Papio_anubis

IFN-α ENSTGEP00000022391_Gelada_Theropithecus_gelada

IFN-α ENSTGEP00000016300_Gelada_Theropithecus_gelada

IFN-α ENSMMUP00000031108_Macaque_Macaca_mulatta

IFN-α ENSMMUP00000077482_Macaque_Macaca_mulatta

IFN-α ENSMFAP00000004263_Crab-eating_macaque_Macaca_fascicularis

IFN-α ENSMFAP00000011265_Crab-eating_macaque_Macaca_fascicularis

IFN-α ENSMFAP00000008630_Crab-eating_macaque_Macaca_fascicularis

IFN-α ENSMNEP00000029214_Pig-tailed_macaque_Macaca_nemestrina

IFN-α ENSMNEP00000023427_Pig-tailed_macaque_Macaca_nemestrina

IFN-α ENSCSAP00000017563_Vervet-AGM_Chlorocebus_sabaeus

IFN-α ENSCSAP00000017564_Vervet-AGM_Chlorocebus_sabaeus

IFN-α ENSPPYP00000021485_Orangutan_Pongo_abelii

IFN-α ENSPTIP00000012429_Tiger_Panthera_tigris_altaica

IFN-α ENSCAFP00000059833_Dog_Canis_lupus_familiaris

IFN-α ENSCAFP00000052302_Dog_Canis_lupus_familiaris

IFN-α ENSCAFP00000053543_Dog_Canis_lupus_familiaris

IFN-α ENSCAFP00000063103_Dog_Canis_lupus_familiaris

IFN-α ENSCAFP00000046496_Dog_Canis_lupus_familiaris

IFN-α ENSCAFP00000047701_Dog_Canis_lupus_familiaris

IFN-α ENSCAFP00000054526_Dog_Canis_lupus_familiaris

IFN-α ENSCAFP00000052916_Dog_Canis_lupus_familiaris

IFN-α ENSCAFP00000049716_Dog_Canis_lupus_familiaris

IFN-α ENSCAFP00000057301_Dog_Canis_lupus_familiaris

IFN-α ENSCAFP00000046622_Dog_Canis_lupus_familiaris

IFN-α ENSCAFP00000045774_Dog_Canis_lupus_familiaris

IFN-α ENSCAFP00000055713_Dog_Canis_lupus_familiaris

IFN-α ENSCAFP00000063864_Dog_Canis_lupus_familiaris

IFN-α ENSCAFP00000062595_Dog_Canis_lupus_familiaris

IFN-α ENSCAFP00040017100_Dog_Canis_lupus_familiaris

IFN-α ENSCAFP00020028494_Dog_Canis_lupus_familiaris

IFN-α ENSCAFP00020028495_Dog_Canis_lupus_familiaris

IFN-α ENSCAFP00020028498_Dog_Canis_lupus_familiaris

IFN-α ENSCAFP00020028492_Dog_Canis_lupus_familiaris

IFN-α ENSCAFP00020017199_Dog_Canis_lupus_familiaris

IFN-α ENSCAFP00000045894_Dog_Canis_lupus_familiaris

IFN-α ENSCAFP00020028488_Dog_Canis_lupus_familiaris

IFN-α ENSCAFP00040034947_Dog_Canis_lupus_familiaris

IFN-α ENSCAFP00030006244_Dog_Canis_lupus_familiaris

IFN-α ENSCAFP00030006278_Dog_Canis_lupus_familiaris

IFN-α ENSCAFP00030006318_Dog_Canis_lupus_familiaris

IFN-α ENSCAFP00030006192_Dog_Canis_lupus_familiaris

IFN-α ENSCAFP00040034928_Dog_Canis_lupus_familiaris

IFN-α ENSCAFP00030006337_Dog_Canis_lupus_familiaris

IFN-α ENSCAFP00030006170_Dog_Canis_lupus_familiaris

IFN-α ENSCAFP00020017196_Dog_Canis_lupus_familiaris

IFN-α ENSCAFP00020017157_Dog_Canis_lupus_familiaris

IFN-α ENSCAFP00020017159_Dog_Canis_lupus_familiaris

IFN-α ENSCAFP00020017163_Dog_Canis_lupus_familiaris

IFN-α ENSCAFP00020017156_Dog_Canis_lupus_familiaris

IFN-α ENSCAFP00040017164_Dog_Canis_lupus_familiaris

IFN-α ENSCAFP00040017187_Dog_Canis_lupus_familiaris

IFN-α ENSCAFP00040017219_Dog_Canis_lupus_familiaris

IFN-α ENSCAFP00020017167_Dog_Canis_lupus_familiaris

IFN-α ENSCAFP00020017183_Dog_Canis_lupus_familiaris

IFN-α ENSCAFP00020017180_Dog_Canis_lupus_familiaris

IFN-α ENSVVUP00000010859_Red_fox_Vulpes_vulpes

IFN-α ENSVVUP00000010863_Red_fox_Vulpes_vulpes

IFN-α ENSVVUP00000010869_Red_fox_Vulpes_vulpes

IFN-α ENSVVUP00000001642_Red_fox_Vulpes_vulpes

IFN-α ENSVVUP00000010855_Red_fox_Vulpes_vulpes

IFN-α ENSVVUP00000001662_Red_fox_Vulpes_vulpes

IFN-α ENSSSUP00005018524_Meerkat_Suricata_suricatta

IFN-α ENSFCAP00000039141_Cat_Felis_catus

IFN-α ENSFCAP00000032900_Cat_Felis_catus

IFN-α ENSLCNP00005034407_Canada_lynx_Lynx_canadensis

IFN-α ENSLCNP00005034411_Canada_lynx_Lynx_canadensis

IFN-α ENSLCNP00005034368_Canada_lynx_Lynx_canadensis

IFN-α ENSLCNP00005034350_Canada_lynx_Lynx_canadensis

IFN-α ENSLCNP00005034363_Canada_lynx_Lynx_canadensis

IFN-α ENSPLOP00000021538_Lion_Panthera_leo

IFN-α ENSPLOP00000011877_Lion_Panthera_leo

IFN-α ENSPPRP00000020723_Leopard_Panthera_pardus

IFN-α ENSNVIP00000015887_American_mink_Neovison_vison

IFN-α ENSNVIP00000015970_American_mink_Neovison_vison

IFN-α ENSNVIP00000015992_American_mink_Neovison_vison

IFN-α ENSNVIP00000008541_American_mink_Neovison_vison

IFN-α ENSNVIP00000015873_American_mink_Neovison_vison

IFN-α ENSNVIP00000015928_American_mink_Neovison_vison

IFN-α ENSNVIP00000015949_American_mink_Neovison_vison

IFN-α ENSMPUP00000019680_Ferret_Mustela_putorius_furo

IFN-α ENSMPUP00000019683_Ferret_Mustela_putorius_furo

IFN-α ENSMPUP00000019688_Ferret_Mustela_putorius_furo

IFN-α ENSMPUP00000019673_Ferret_Mustela_putorius_furo

IFN-α ENSMPUP00000019675_Ferret_Mustela_putorius_furo

IFN-α ENSMPUP00000019676_Ferret_Mustela_putorius_furo

IFN-α ENSMPUP00000019385_Ferret_Mustela_putorius_furo

IFN-α ENSMPUP00000019392_Ferret_Mustela_putorius_furo

IFN-α ENSMPUP00000019395_Ferret_Mustela_putorius_furo

IFN-α ENSMPUP00000019690_Ferret_Mustela_putorius_furo

IFN-α ENSMPUP00000019371_Ferret_Mustela_putorius_furo

IFN-α ENSMPUP00000019378_Ferret_Mustela_putorius_furo

IFN-α ENSMPUP00000019670_Ferret_Mustela_putorius_furo

IFN-α ENSZCAP00015022380_California_sea_lion_Zalophus_californianus

IFN-α ENSZCAP00015022358_California_sea_lion_Zalophus_californianus

IFN-α ENSZCAP00015022296_California_sea_lion_Zalophus_californianus

IFN-α ENSZCAP00015022471_California_sea_lion_Zalophus_californianus

IFN-α ENSZCAP00015022440_California_sea_lion_Zalophus_californianus

IFN-α ENSZCAP00015022412_California_sea_lion_Zalophus_californianus

IFN-α ENSUTTP00000020013_Asiatic_black_bear_Ursus_thibetanus_thibetanus

IFN-α ENSUTTP00000020021_Asiatic_black_bear_Ursus_thibetanus_thibetanus

IFN-α ENSUAMP00000006118_American_black_bear_Ursus_americanus

IFN-α ENSUAMP00000019351_American_black_bear_Ursus_americanus

IFN-α ENSUAMP00000003212_American_black_bear_Ursus_americanus

IFN-α ENSUAMP00000011530_American_black_bear_Ursus_americanus

IFN-α ENSUMAP00000034409_Polar_bear_Ursus_maritimus

IFN-α ENSUMAP00000034407_Polar_bear_Ursus_maritimus

IFN-α ENSUMAP00000021851_Polar_bear_Ursus_maritimus

IFN-α ENSUMAP00000007187_Polar_bear_Ursus_maritimus

IFN-α ENSUMAP00000021899_Polar_bear_Ursus_maritimus

IFN-α ENSAMEP00000003186_Panda_Ailuropoda_melanoleuca

IFN-α ENSAMEP00000003181_Panda_Ailuropoda_melanoleuca

IFN-α ENSAMEP00000021292_Panda_Ailuropoda_melanoleuca

IFN-α ENSAMEP00000021294_Panda_Ailuropoda_melanoleuca

IFN-α ENSAMEP00000003198_Panda_Ailuropoda_melanoleuca

IFN-α ENSOGAP00000018787_Bushbaby_Otolemur_garnettii

IFN-α ENSLAFP00000028352_Elephant_Loxodonta_africana

IFN-α ENSLAFP00000013786_Elephant_Loxodonta_africana

IFN-α ENSLAFP00000021500_Elephant_Loxodonta_africana

IFN-α ENSLAFP00000025490_Elephant_Loxodonta_africana

IFN-α ENSCDRP00005002178_Arabian_camel_Camelus_dromedarius

IFN-α ENSCDRP00005002166_Arabian_camel_Camelus_dromedarius

IFN-α ENSVPAP00000011192_Alpaca_Vicugna_pacos

IFN-α ENSVPAP00000011785_Alpaca_Vicugna_pacos

IFN-α ENSCWAP00000017282_Chacoan_peccary_Catagonus_wagneri

IFN-α ENSCWAP00000016961_Chacoan_peccary_Catagonus_wagneri

IFN-α ENSCWAP00000016824_Chacoan_peccary_Catagonus_wagneri

IFN-α ENSCWAP00000017189_Chacoan_peccary_Catagonus_wagneri

IFN-α ENSCWAP00000017071_Chacoan_peccary_Catagonus_wagneri

IFN-α ENSSSCP00000053795_Pig_Sus_scrofa

IFN-α ENSSSCP00000063698_Pig_Sus_scrofa

IFN-α ENSSSCP00000067859_Pig_Sus_scrofa

IFN-α ENSSSCP00000063734_Pig_Sus_scrofa

IFN-α ENSSSCP00000067458_Pig_Sus_scrofa

IFN-α ENSSSCP00000047731_Pig_Sus_scrofa

IFN-α ENSPCTP00005022823_Sperm_whale_Physeter_catodon

IFN-α ENSPCTP00005022970_Sperm_whale_Physeter_catodon

IFN-α ENSPCTP00005022772_Sperm_whale_Physeter_catodon

IFN-α ENSPCTP00005023099_Sperm_whale_Physeter_catodon

IFN-α ENSBMSP00010003566_Blue_whale_Balaenoptera_musculus

IFN-α ENSBMSP00010003572_Blue_whale_Balaenoptera_musculus

IFN-α ENSBMSP00010003599_Blue_whale_Balaenoptera_musculus

IFN-α ENSBMSP00010000101_Blue_whale_Balaenoptera_musculus

IFN-α ENSBMSP00010000169_Blue_whale_Balaenoptera_musculus

IFN-α ENSPSNP00000001390_Vaquita_Phocoena_sinus

IFN-α ENSPSNP00000001484_Vaquita_Phocoena_sinus

IFN-α ENSPSNP00000001464_Vaquita_Phocoena_sinus

IFN-α ENSMMNP00015021685_Narwhal_Monodon_monoceros

IFN-α ENSMMNP00015021842_Narwhal_Monodon_monoceros

IFN-α ENSMMNP00015021729_Narwhal_Monodon_monoceros

IFN-α ENSTTRP00000007667_Dolphin_Tursiops_truncatus

IFN-α ENSEEUP00000010918_Hedgehog_Erinaceus_europaeus

IFN-α ENSEEUP00000007279_Hedgehog_Erinaceus_europaeus

IFN-α ENSPCAP00000012700_Hyrax_Procavia_capensis

IFN-α ENSPCAP00000009838_Hyrax_Procavia_capensis

IFN-α ENSPCAP00000004368_Hyrax_Procavia_capensis

IFN-α ENSMOCP00000004223_Prairie_vole_Microtus_ochrogaster

IFN-α ENSMOCP00000004827_Prairie_vole_Microtus_ochrogaster

IFN-α ENSMOCP00000004419_Prairie_vole_Microtus_ochrogaster

IFN-α ENSMOCP00000005012_Prairie_vole_Microtus_ochrogaster

IFN-α ENSMOCP00000027457_Prairie_vole_Microtus_ochrogaster

IFN-α ENSMOCP00000004368_Prairie_vole_Microtus_ochrogaster

IFN-α ENSMOCP00000007344_Prairie_vole_Microtus_ochrogaster

IFN-α ENSMOCP00000003645_Prairie_vole_Microtus_ochrogaster

IFN-α ENSMOCP00000004148_Prairie_vole_Microtus_ochrogaster

IFN-α ENSPEMP00000034251_Northern_American_deer_mouse_Peromyscus_maniculatus_bairdii

IFN-α ENSPEMP00000035642_Northern_American_deer_mouse_Peromyscus_maniculatus_bairdii

IFN-α ENSPEMP00000036980_Northern_American_deer_mouse_Peromyscus_maniculatus_bairdii

IFN-α ENSPEMP00000034692_Northern_American_deer_mouse_Peromyscus_maniculatus_bairdii

IFN-α ENSPEMP00000029644_Northern_American_deer_mouse_Peromyscus_maniculatus_bairdii

IFN-α ENSPEMP00000033408_Northern_American_deer_mouse_Peromyscus_maniculatus_bairdii

IFN-α ENSPEMP00000031360_Northern_American_deer_mouse_Peromyscus_maniculatus_bairdii

IFN-α ENSPEMP00000030353_Northern_American_deer_mouse_Peromyscus_maniculatus_bairdii

IFN-α ENSPEMP00000029332_Northern_American_deer_mouse_Peromyscus_maniculatus_bairdii

IFN-α ENSPEMP00000034387_Northern_American_deer_mouse_Peromyscus_maniculatus_bairdii

IFN-α ENSPEMP00000030136_Northern_American_deer_mouse_Peromyscus_maniculatus_bairdii

IFN-α ENSPEMP00000031797_Northern_American_deer_mouse_Peromyscus_maniculatus_bairdii

IFN-α ENSPEMP00000031347_Northern_American_deer_mouse_Peromyscus_maniculatus_bairdii

IFN-α ENSPEMP00000029641_Northern_American_deer_mouse_Peromyscus_maniculatus_bairdii

IFN-α ENSPEMP00000033217_Northern_American_deer_mouse_Peromyscus_maniculatus_bairdii

IFN-α ENSPEMP00000036499_Northern_American_deer_mouse_Peromyscus_maniculatus_bairdii

IFN-α ENSPEMP00000031730_Northern_American_deer_mouse_Peromyscus_maniculatus_bairdii

IFN-α ENSPEMP00000034448_Northern_American_deer_mouse_Peromyscus_maniculatus_bairdii

IFN-α ENSPEMP00000036386_Northern_American_deer_mouse_Peromyscus_maniculatus_bairdii

IFN-α ENSPEMP00000029157_Northern_American_deer_mouse_Peromyscus_maniculatus_bairdii

IFN-α ENSPEMP00000036269_Northern_American_deer_mouse_Peromyscus_maniculatus_bairdii

IFN-α ENSPEMP00000031172_Northern_American_deer_mouse_Peromyscus_maniculatus_bairdii

IFN-α ENSPEMP00000030885_Northern_American_deer_mouse_Peromyscus_maniculatus_bairdii

IFN-α ENSMAUP00000003981_Golden_Hamster_Mesocricetus_auratus

IFN-α ENSMUSP00000100774_Mouse_Mus_musculus

IFN-α ENSMUSP00000071333_Mouse_Mus_musculus

IFN-α ENSMUSP00000100779_Mouse_Mus_musculus

IFN-α ENSMUSP00000100778_Mouse_Mus_musculus

IFN-α ENSMUSP00000092581_Mouse_Mus_musculus

IFN-α ENSMUSP00000092580_Mouse_Mus_musculus

IFN-α ENSMUSP00000099868_Mouse_Mus_musculus

IFN-α ENSMUSP00000127921_Mouse_Mus_musculus

IFN-α ENSMUSP00000100777_Mouse_Mus_musculus

IFN-α ENSMUSP00000100780_Mouse_Mus_musculus

IFN-α ENSMUSP00000099871_Mouse_Mus_musculus

IFN-α ENSMUSP00000099870_Mouse_Mus_musculus

IFN-α ENSMUSP00000099873_Mouse_Mus_musculus

IFN-α ENSMUSP00000099872_Mouse_Mus_musculus

IFN-α ENSMSIP00000037998_Steppe_mouse_Mus_spicilegus

IFN-α ENSMSIP00000037997_Steppe_mouse_Mus_spicilegus

IFN-α ENSMSIP00000018756_Steppe_mouse_Mus_spicilegus

IFN-α ENSMSIP00000018751_Steppe_mouse_Mus_spicilegus

IFN-α ENSMSIP00000037985_Steppe_mouse_Mus_spicilegus

IFN-α ENSMSIP00000037993_Steppe_mouse_Mus_spicilegus

IFN-α ENSMSIP00000003140_Steppe_mouse_Mus_spicilegus

IFN-α ENSMSIP00000037982_Steppe_mouse_Mus_spicilegus

IFN-α ENSMUGP00000008033_Mongolian_gerbil_Meriones_unguiculatus

IFN-α ENSMUGP00000029071_Mongolian_gerbil_Meriones_unguiculatus

IFN-α ENSMUGP00000008041_Mongolian_gerbil_Meriones_unguiculatus

IFN-α ENSMUGP00000006118_Mongolian_gerbil_Meriones_unguiculatus

IFN-α ENSMUGP00000001678_Mongolian_gerbil_Meriones_unguiculatus

IFN-α ENSRNOP00000055160_Rat_Rattus_norvegicus

IFN-α ENSRNOP00000075265_Rat_Rattus_norvegicus

IFN-α ENSRNOP00000072648_Rat_Rattus_norvegicus

IFN-α ENSRNOP00000043498_Rat_Rattus_norvegicus

IFN-α ENSRNOP00000042399_Rat_Rattus_norvegicus

IFN-α ENSRNOP00000073431_Rat_Rattus_norvegicus

IFN-α ENSRNOP00000048643_Rat_Rattus_norvegicus

IFN-α ENSRNOP00000058900_Rat_Rattus_norvegicus

IFN-α ENSRNOP00000051043_Rat_Rattus_norvegicus

IFN-α ENSRNOP00000041463_Rat_Rattus_norvegicus

IFN-α ENSNGAP00000012458_Upper_Galilee_mountains_blind_mole_rat_Nannospalax_galili

IFN-α ENSCGRP00015022181_Chinese_hamster_CriGri_Cricetulus_griseus

IFN-α ENSCGRP00015022167_Chinese_hamster_CriGri_Cricetulus_griseus

IFN-α ENSCGRP00015022185_Chinese_hamster_CriGri_Cricetulus_griseus

IFN-α ENSCGRP00000026614_Chinese_hamster_CriGri_Cricetulus_griseus

IFN-α ENSCGRP00000026517_Chinese_hamster_CriGri_Cricetulus_griseus

IFN-α ENSCGRP00015022002_Chinese_hamster_CriGri_Cricetulus_griseus

IFN-α ENSCGRP00015022039_Chinese_hamster_CriGri_Cricetulus_griseus

IFN-α ENSCGRP00015022046_Chinese_hamster_CriGri_Cricetulus_griseus

IFN-α ENSCGRP00015022153_Chinese_hamster_CriGri_Cricetulus_griseus

IFN-α ENSCGRP00015022161_Chinese_hamster_CriGri_Cricetulus_griseus

IFN-α ENSCGRP00000026436_Chinese_hamster_CriGri_Cricetulus_griseus

IFN-α ENSCGRP00000026043_Chinese_hamster_CriGri_Cricetulus_griseus

IFN-α ENSCGRP00000017850_Chinese_hamster_CriGri_Cricetulus_griseus

IFN-α ENSCGRP00000008433_Chinese_hamster_CriGri_Cricetulus_griseus

IFN-α ENSCGRP00000026017_Chinese_hamster_CriGri_Cricetulus_griseus

IFN-α ENSCGRP00000025920_Chinese_hamster_CriGri_Cricetulus_griseus

IFN-α ENSCGRP00000026119_Chinese_hamster_CriGri_Cricetulus_griseus

IFN-α ENSCGRP00000026756_Chinese_hamster_CriGri_Cricetulus_griseus

IFN-α ENSCGRP00000026085_Chinese_hamster_CriGri_Cricetulus_griseus

IFN-α ENSCGRP00000026409_Chinese_hamster_CriGri_Cricetulus_griseus

IFN-α ENSCGRP00000026074_Chinese_hamster_CriGri_Cricetulus_griseus

IFN-α ENSCGRP00001005273_Chinese_hamster_CriGri_Cricetulus_griseus

IFN-α ENSCGRP00001005185_Chinese_hamster_CriGri_Cricetulus_griseus

IFN-α ENSCGRP00001004472_Chinese_hamster_CriGri_Cricetulus_griseus

IFN-α ENSCGRP00001019639_Chinese_hamster_CriGri_Cricetulus_griseus

IFN-α ENSCGRP00001005705_Chinese_hamster_CriGri_Cricetulus_griseus

IFN-α ENSCGRP00001005692_Chinese_hamster_CriGri_Cricetulus_griseus

IFN-α ENSCGRP00001004432_Chinese_hamster_CriGri_Cricetulus_griseus

IFN-α ENSCGRP00015022142_Chinese_hamster_CriGri_Cricetulus_griseus

IFN-α ENSCGRP00001007172_Chinese_hamster_CriGri_Cricetulus_griseus

IFN-α ENSCGRP00001007002_Chinese_hamster_CriGri_Cricetulus_griseus

IFN-α ENSCGRP00001004560_Chinese_hamster_CriGri_Cricetulus_griseus

IFN-α ENSCGRP00015022057_Chinese_hamster_CriGri_Cricetulus_griseus

IFN-α ENSCGRP00015022062_Chinese_hamster_CriGri_Cricetulus_griseus

IFN-α ENSCGRP00015022133_Chinese_hamster_CriGri_Cricetulus_griseus

IFN-α ENSCGRP00015022072_Chinese_hamster_CriGri_Cricetulus_griseus

IFN-α ENSCGRP00015022118_Chinese_hamster_CriGri_Cricetulus_griseus

IFN-α ENSCGRP00001002739_Chinese_hamster_CriGri_Cricetulus_griseus

IFN-α ENSCGRP00001002358_Chinese_hamster_CriGri_Cricetulus_griseus

IFN-α ENSCGRP00001002604_Chinese_hamster_CriGri_Cricetulus_griseus

IFN-α ENSCGRP00001001705_Chinese_hamster_CriGri_Cricetulus_griseus

IFN-α ENSCGRP00001003128_Chinese_hamster_CriGri_Cricetulus_griseus

IFN-α ENSSVLP00005029432_Eurasian_red_squirrel_Sciurus_vulgaris

IFN-α ENSSVLP00005029459_Eurasian_red_squirrel_Sciurus_vulgaris

IFN-α ENSSVLP00005029476_Eurasian_red_squirrel_Sciurus_vulgaris

IFN-α ENSSVLP00005029397_Eurasian_red_squirrel_Sciurus_vulgaris

IFN-α ENSSVLP00005029202_Eurasian_red_squirrel_Sciurus_vulgaris

IFN-α ENSSVLP00005029347_Eurasian_red_squirrel_Sciurus_vulgaris

IFN-α ENSSVLP00005029154_Eurasian_red_squirrel_Sciurus_vulgaris

IFN-α ENSSVLP00005029223_Eurasian_red_squirrel_Sciurus_vulgaris

IFN-α ENSSVLP00005029378_Eurasian_red_squirrel_Sciurus_vulgaris

IFN-α ENSSVLP00005029326_Eurasian_red_squirrel_Sciurus_vulgaris

IFN-α ENSSVLP00005029298_Eurasian_red_squirrel_Sciurus_vulgaris

IFN-α ENSSDAP00000017064_Daurian_ground_squirrel_Spermophilus_dauricus

IFN-α ENSSDAP00000017044_Daurian_ground_squirrel_Spermophilus_dauricus

IFN-α ENSSDAP00000017005_Daurian_ground_squirrel_Spermophilus_dauricus

IFN-α ENSSDAP00000017018_Daurian_ground_squirrel_Spermophilus_dauricus

IFN-α ENSSDAP00000017035_Daurian_ground_squirrel_Spermophilus_dauricus

IFN-α ENSSDAP00000022974_Daurian_ground_squirrel_Spermophilus_dauricus

IFN-α ENSSDAP00000016990_Daurian_ground_squirrel_Spermophilus_dauricus

IFN-α ENSSDAP00000027536_Daurian_ground_squirrel_Spermophilus_dauricus

IFN-α ENSSDAP00000017067_Daurian_ground_squirrel_Spermophilus_dauricus

IFN-α ENSSDAP00000016997_Daurian_ground_squirrel_Spermophilus_dauricus

IFN-α ENSUPAP00010009379_Arctic_ground_squirrel_Urocitellus_parryii

IFN-α ENSUPAP00010024252_Arctic_ground_squirrel_Urocitellus_parryii

IFN-α ENSUPAP00010024250_Arctic_ground_squirrel_Urocitellus_parryii

IFN-α ENSUPAP00010022402_Arctic_ground_squirrel_Urocitellus_parryii

IFN-α ENSUPAP00010022417_Arctic_ground_squirrel_Urocitellus_parryii

IFN-α ENSMMMP00000006669_Alpine_marmot_Marmota_marmota_marmota

IFN-α ENSMMMP00000006659_Alpine_marmot_Marmota_marmota_marmota

IFN-α ENSMMMP00000006649_Alpine_marmot_Marmota_marmota_marmota

IFN-α ENSMMMP00000006692_Alpine_marmot_Marmota_marmota_marmota

IFN-α ENSMMMP00000006696_Alpine_marmot_Marmota_marmota_marmota

IFN-α ENSMMMP00000006679_Alpine_marmot_Marmota_marmota_marmota

IFN-α ENSMMMP00000010847_Alpine_marmot_Marmota_marmota_marmota

IFN-α ENSMMMP00000006617_Alpine_marmot_Marmota_marmota_marmota

IFN-α ENSMMMP00000006645_Alpine_marmot_Marmota_marmota_marmota

IFN-α ENSSTOP00000026679_Squirrel_Ictidomys_tridecemlineatus

IFN-α ENSSTOP00000028887_Squirrel_Ictidomys_tridecemlineatus

IFN-α ENSODEP00000005345_Degu_Octodon_degus

IFN-α ENSODEP00000005410_Degu_Octodon_degus

IFN-α ENSCAPP00000001226_Brazilian_guinea_pig_Cavia_aperea

IFN-α ENSCAPP00000001420_Brazilian_guinea_pig_Cavia_aperea

IFN-α ENSCAPP00000012450_Brazilian_guinea_pig_Cavia_aperea

IFN-α ENSCPOP00000021784_Guinea_Pig_Cavia_porcellus

IFN-α ENSCPOP00000030203_Guinea_Pig_Cavia_porcellus

IFN-α ENSCPOP00000031224_Guinea_Pig_Cavia_porcellus

IFN-α ENSCPOP00000020885_Guinea_Pig_Cavia_porcellus

IFN-α ENSCPOP00000024866_Guinea_Pig_Cavia_porcellus

IFN-α ENSCPOP00000031690_Guinea_Pig_Cavia_porcellus

IFN-α ENSCPOP00000018061_Guinea_Pig_Cavia_porcellus

IFN-α ENSCPOP00000021491_Guinea_Pig_Cavia_porcellus

IFN-α ENSCPOP00000027319_Guinea_Pig_Cavia_porcellus

IFN-α ENSCPOP00000023540_Guinea_Pig_Cavia_porcellus

IFN-α ENSCPOP00000030562_Guinea_Pig_Cavia_porcellus

IFN-α ENSCPOP00000020331_Guinea_Pig_Cavia_porcellus

IFN-α ENSFDAP00000012025_Damara_mole_rat_Fukomys_damarensis

IFN-α ENSHGLP00100027418_Naked_mole-rat_female_Heterocephalus_glaber

IFN-α ENSHGLP00000027622_Naked_mole-rat_female_Heterocephalus_glaber

IFN-α ENSCLAP00000017420_Long-tailed_chinchilla_Chinchilla_lanigera

IFN-α MGP_CAROLIEiJ_P0062914_Ryukyu_mouse_Mus_caroli

IFN-α MGP_CAROLIEiJ_P0062920_Ryukyu_mouse_Mus_caroli

IFN-α MGP_CAROLIEiJ_P0062912_Ryukyu_mouse_Mus_caroli

IFN-α MGP_CAROLIEiJ_P0062913_Ryukyu_mouse_Mus_caroli

IFN-α MGP_CAROLIEiJ_P0062923_Ryukyu_mouse_Mus_caroli

IFN-α MGP_CAROLIEiJ_P0062922_Ryukyu_mouse_Mus_caroli

IFN-α MGP_SPRETEiJ_P0065401_Algerian_mouse_Mus_spretus

IFN-α MGP_SPRETEiJ_P0065395_Algerian_mouse_Mus_spretus

IFN-α MGP_SPRETEiJ_P0065403_Algerian_mouse_Mus_spretus

IFN-α MGP_SPRETEiJ_P0065408_Algerian_mouse_Mus_spretus

IFN-α MGP_SPRETEiJ_P0065407_Algerian_mouse_Mus_spretus

IFN-α MGP_SPRETEiJ_P0065404_Algerian_mouse_Mus_spretus

IFN-α MGP_PahariEiJ_P0078050_Shrew_mouse_Mus_pahari

IFN-α MGP_PahariEiJ_P0078047_Shrew_mouse_Mus_pahari

IFN-α MGP_PahariEiJ_P0078048_Shrew_mouse_Mus_pahari

IFN-α MGP_PahariEiJ_P0078049_Shrew_mouse_Mus_pahari

IFN-α MGP_PahariEiJ_P0078054_Shrew_mouse_Mus_pahari

IFN-α ENSCCNP00000005371_American_beaver_Castor_canadensis

IFN-α ENSCCNP00000005028_American_beaver_Castor_canadensis

IFN-α ENSCCNP00000009218_American_beaver_Castor_canadensis

IFN-α ENSCCNP00000005326_American_beaver_Castor_canadensis

IFN-α ENSCCNP00000005350_American_beaver_Castor_canadensis

IFN-α ENSCCNP00000005108_American_beaver_Castor_canadensis

IFN-α ENSCCNP00000005357_American_beaver_Castor_canadensis

IFN-α ENSCCNP00000005352_American_beaver_Castor_canadensis

IFN-α ENSECAP00000038987_Horse_Equus_caballus

IFN-α ENSECAP00000004795_Horse_Equus_caballus

IFN-α ENSECAP00000040329_Horse_Equus_caballus

IFN-α ENSECAP00000026656_Horse_Equus_caballus

IFN-α ENSECAP00000050755_Horse_Equus_caballus

IFN-α ENSECAP00000042726_Horse_Equus_caballus

IFN-α ENSEASP00005032794_Donkey_Equus_asinus_asinus

IFN-α ENSEASP00005032802_Donkey_Equus_asinus_asinus

IFN-α ENSEASP00005032827_Donkey_Equus_asinus_asinus

IFN-α ENSEASP00005032824_Donkey_Equus_asinus_asinus

IFN-α ENSEASP00005032810_Donkey_Equus_asinus_asinus

IFN-γ ENSOANP00000009443_Platypus_Ornithorhynchus_anatinus

IFN-γ ENSSHAP00000017704_Tasmanian_devil_Sarcophilus_harrisii

IFN-γ ENSVURP00010031608_Common_wombat_Vombatus_ursinus

IFN-γ ENSPCIP00000025898_Koala_Phascolarctos_cinereus

IFN-γ ENSTSYP00000001471_Tarsier_Carlito_syrichta

IFN-γ ENSCCAP00000019170_Capuchin_Cebus_capucinus_imitator

IFN-γ ENSCJAP00000056963_Marmoset_Callithrix_jacchus

IFN-γ ENSANAP00000001024_Ma's_night_monkey_Aotus_nancymaae

IFN-γ ENSSBOP00000037274_Bolivian_squirrel_monkey_Saimiri_boliviensis_boliviensis

IFN-γ ENSPPYP00000005405_Orangutan_Pongo_abelii

IFN-γ ENSNLEP00000004233_Gibbon_Nomascus_leucogenys

IFN-γ ENSGGOP00000005908_Gorilla_Gorilla_gorilla_gorilla

IFN-γ ENSP00000229135

IFN-γ ENSPTRP00000008822_Chimpanzee_Pan_troglodytes

IFN-γ ENSPPAP00000038608_Bonobo_Pan_paniscus

IFN-γ ENSRBIP00000031792_Black_snub-nosed_monkey_Rhinopithecus_bieti

IFN-γ ENSCANP00000014463_Angola_colobus_Colobus_angolensis_palliatus

IFN-γ ENSMLEP00000021950_Drill_Mandrillus_leucophaeus

IFN-γ ENSCSAP00000000682_Vervet-AGM_Chlorocebus_sabaeus

IFN-γ ENSTGEP00000027025_Gelada_Theropithecus_gelada

IFN-γ ENSPTEP00000033649_Ugandan_red_Colobus_Piliocolobus_tephrosceles

IFN-γ ENSRROP00000017582_Golden_snub-nosed_monkey_Rhinopithecus_roxellana

IFN-γ ENSCATP00000022573_Sooty_mangabey_Cercocebus_atys

IFN-γ ENSPANP00000001825_Olive_baboon_Papio_anubis

IFN-γ ENSMFAP00000024297_Crab-eating_macaque_Macaca_fascicularis

IFN-γ ENSMNEP00000045088_Pig-tailed_macaque_Macaca_nemestrina

IFN-γ ENSMMUP00000065064_Macaque_Macaca_mulatta

IFN-γ ENSOGAP00000003836_Bushbaby_Otolemur_garnettii

IFN-γ ENSMICP00000003845_Mouse_Lemur_Microcebus_murinus

IFN-γ ENSPSMP00000002481_Greater_bamboo_lemur_Prolemur_simus

IFN-γ ENSPCOP00000020761_Coquerel's_sifaka_Propithecus_coquereli

IFN-γ ENSCLAP00000013803_Long-tailed_chinchilla_Chinchilla_lanigera

IFN-γ ENSCPOP00000009919_Guinea_Pig_Cavia_porcellus

IFN-γ ENSCAPP00000015598_Brazilian_guinea_pig_Cavia_aperea

IFN-γ ENSFDAP00000000699_Damara_mole_rat_Fukomys_damarensis

IFN-γ ENSHGLP00100021036_Naked_mole-rat_female_Heterocephalus_glaber

IFN-γ ENSHGLP00000013116_Naked_mole-rat_female_Heterocephalus_glaber

IFN-γ ENSSVLP00005017258_Eurasian_red_squirrel_Sciurus_vulgaris

IFN-γ ENSMMMP00000007081_Alpine_marmot_Marmota_marmota_marmota

IFN-γ ENSSDAP00000004060_Daurian_ground_squirrel_Spermophilus_dauricus

IFN-γ ENSSTOP00000013397_Squirrel_Ictidomys_tridecemlineatus

IFN-γ ENSUPAP00010029638_Arctic_ground_squirrel_Urocitellus_parryii

IFN-γ ENSCCNP00000006355_American_beaver_Castor_canadensis

IFN-γ ENSMOCP00000014947_Prairie_vole_Microtus_ochrogaster

IFN-γ ENSPEMP00000001426_Northern_American_deer_mouse_Peromyscus_maniculatus_bairdii

IFN-γ ENSMAUP00000013711_Golden_Hamster_Mesocricetus_auratus

IFN-γ ENSCGRP00015005739_Chinese_hamster_CHOK1GS_Cricetulus_griseus

IFN-γ ENSCGRP00001005117_Chinese_hamster_CHOK1GS_Cricetulus_griseus

IFN-γ ENSCGRP00000013616_Chinese_hamster_CHOK1GS_Cricetulus_griseus

IFN-γ ENSMUGP00000004162_Mongolian_gerbil_Meriones_unguiculatus

IFN-γ ENSRNOP00000009917_Rat_Rattus_norvegicus

IFN-γ MGP_PahariEiJ_P0087814

IFN-γ MGP_CAROLIEiJ_P0024852

IFN-γ MGP_SPRETEiJ_P0026106

IFN-γ ENSMUSP00000063800_Mouse_Mus_musculus

IFN-γ ENSMSIP00000012277_Steppe_mouse_Mus_spicilegus

IFN-γ ENSDNOP00000003931_Armadillo_Dasypus_novemcinctus

IFN-γ ENSETEP00000006202_Lesser_hedgehog_tenrec_Echinops_telfairi

IFN-γ ENSPCAP00000010797_Hyrax_Procavia_capensis

IFN-γ ENSLAFP00000003740_Elephant_Loxodonta_africana

IFN-γ ENSRFEP00010003332_Greater_horseshoe_bat_Rhinolophus_ferrumequinum

IFN-γ ENSMLUP00000002447_Microbat_Myotis_lucifugus

IFN-γ ENSPVAP00000014187_Megabat_Pteropus_vampyrus

IFN-γ ENSECAP00000030547_Horse_Equus_caballus

IFN-γ ENSEASP00005011380_Donkey_Equus_asinus_asinus

IFN-γ ENSSSUP00005031115_Meerkat_Suricata_suricatta

IFN-γ ENSLCNP00005003190_Canada_lynx_Lynx_canadensis

IFN-γ ENSFCAP00000008355_Cat_Felis_catus

IFN-γ ENSPTIP00000017209_Tiger_Panthera_tigris_altaica

IFN-γ ENSPPRP00000017115_Leopard_Panthera_pardus

IFN-γ ENSPLOP00000020324_Lion_Panthera_leo

IFN-γ ENSVVUP00000014058_Red_fox_Vulpes_vulpes

IFN-γ ENSCAFP00000000588_Dingo_Canis_lupus_dingo

IFN-γ ENSCAFP00020021553_Dingo_Canis_lupus_dingo

IFN-γ ENSCAFP00040036908_Dingo_Canis_lupus_dingo

IFN-γ ENSCAFP00030016532_Dingo_Canis_lupus_dingo

IFN-γ ENSNVIP00000012608_American_mink_Neovison_vison

IFN-γ ENSMPUP00000017401_Ferret_Mustela_putorius_furo

IFN-γ ENSZCAP00015039676_California_sea_lion_Zalophus_californianus

IFN-γ ENSAMEP00000009180_Panda_Ailuropoda_melanoleuca

IFN-γ ENSUTTP00000000011_Asiatic_black_bear_Ursus_thibetanus_thibetanus

IFN-γ ENSUMAP00000028473_Polar_bear_Ursus_maritimus

IFN-γ ENSCDRP00005019497_Arabian_camel_Camelus_dromedarius

IFN-γ ENSSSCP00000036242_Pig_Sus_scrofa

IFN-γ ENSCWAP00000027781_Chacoan_peccary_Catagonus_wagneri

IFN-γ ENSPCTP00005004159_Sperm_whale_Physeter_catodon

IFN-γ ENSBMSP00010022167_Blue_whale_Balaenoptera_musculus

IFN-γ ENSTTRP00000006137_Dolphin_Tursiops_truncatus

IFN-γ ENSPSNP00000006759_Vaquita_Phocoena_sinus

IFN-γ ENSMMNP00015027916_Narwhal_Monodon_monoceros

IFN-γ ENSDLEP00000002943_Beluga_whale_Delphinapterus_leucas

IFN-γ ENSCHYP00000004381_Yarkand_deer_Cervus_hanglu_yarkandensis

IFN-γ ENSMMSP00000025262_Siberian_musk_deer_Moschus_moschiferus

IFN-γ ENSOARP00000002060_Sheep_texel_

IFN-γ ENSCHIP00000016152_Goat_Capra_hircus

IFN-γ ENSBBBP00000019468_American_bison_Bison_bison_bison

IFN-γ ENSBMUP00000015309_Wild_yak_Bos_mutus

IFN-γ ENSBGRP00000032753_Domestic_yak_Bos_grunniens

IFN-γ ENSBIXP00005002394_Hybrid_-_Bos_Indicus_Bos_indicus_x_Bos_taurus

IFN-γ ENSBTAP00000016634_Cow_Bos_taurus

IFN-γ ENSBIXP00000036945_Hybrid_-_Bos_Indicus_Bos_indicus_x_Bos_taurus

IFN-β ENSMEUP00000013050_Wallaby_Notamacropus_eugenii

IFN-β ENSPCIP00000030557_Koala_Phascolarctos_cinereus

IFN-β ENSPCIP00000054679_Koala_Phascolarctos_cinereus

IFN-β ENSPCIP00000047283_Koala_Phascolarctos_cinereus

IFN-β ENSPCIP00000054105_Koala_Phascolarctos_cinereus

IFN-β ENSVURP00010016291_Common_wombat_Vombatus_ursinus

IFN-β ENSMODP00000037231_Opossum_Monodelphis_domestica

IFN-β ENSSHAP00000020810_Tasmanian_devil_Sarcophilus_harrisii

IFN-β ENSOANP00000048849_Platypus_Ornithorhynchus_anatinus

IFN-β ENSOANP00000009491_Platypus_Ornithorhynchus_anatinus

IFN-β ENSDNOP00000004636_Armadillo_Dasypus_novemcinctus

IFN-β ENSMLUP00000020233_Microbat_Myotis_lucifugus

IFN-β ENSRFEP00010011850_Greater_horseshoe_bat_Rhinolophus_ferrumequinum

IFN-β ENSPSMP00000006701_Greater_bamboo_lemur_Prolemur_simus

IFN-β ENSPSMP00000003527_Greater_bamboo_lemur_Prolemur_simus

IFN-β ENSPSMP00000006694_Greater_bamboo_lemur_Prolemur_simus

IFN-β ENSOGAP00000016718_Bushbaby_Otolemur_garnettii

IFN-β ENSGGOP00000016838_Gorilla_Gorilla_gorilla_gorilla

IFN-β ENSP00000369581

IFN-β ENSPTRP00000035597_Chimpanzee_Pan_troglodytes

IFN-β ENSPPYP00000021493_Orangutan_Pongo_abelii

IFN-β ENSNLEP00000022842_Gibbon_Nomascus_leucogenys

IFN-β ENSPTEP00000012105_Ugandan_red_Colobus_Piliocolobus_tephrosceles

IFN-β ENSMNEP00000028961_Pig-tailed_macaque_Macaca_nemestrina

IFN-β ENSCANP00000028836_Angola_colobus_Colobus_angolensis_palliatus

IFN-β ENSPANP00000010147_Olive_baboon_Papio_anubis

IFN-β ENSTGEP00000016752_Gelada_Theropithecus_gelada

IFN-β ENSMFAP00000013528_Crab-eating_macaque_Macaca_fascicularis

IFN-β ENSMMUP00000025064_Macaque_Macaca_mulatta

IFN-β ENSCATP00000006144_Sooty_mangabey_Cercocebus_atys

IFN-β ENSRBIP00000004555_Black_snub-nosed_monkey_Rhinopithecus_bieti

IFN-β ENSRROP00000000706_Golden_snub-nosed_monkey_Rhinopithecus_roxellana

IFN-β ENSCSAP00000017571_Vervet-AGM_Chlorocebus_sabaeus

IFN-β ENSCJAP00000069357_Marmoset_Callithrix_jacchus

IFN-β ENSZCAP00015022554_California_sea_lion_Zalophus_californianus

IFN-β ENSMPUP00000019400_Ferret_Mustela_putorius_furo

IFN-β ENSNVIP00000016013_American_mink_Neovison_vison

IFN-β ENSAMEP00000021291_Panda_Ailuropoda_melanoleuca

IFN-β ENSCAFP00030006431_Dog_Canis_lupus_familiaris

IFN-β ENSCAFP00000002410_Dog_Canis_lupus_familiaris

IFN-β ENSVVUP00000001627_Red_fox_Vulpes_vulpes

IFN-β ENSPPRP00000029362_Leopard_Panthera_pardus

IFN-β ENSPTIP00000003917_Tiger_Panthera_tigris_altaica

IFN-β ENSFCAP00000004253_Cat_Felis_catus

IFN-β ENSSSUP00005008257_Meerkat_Suricata_suricatta

IFN-β ENSLAFP00000028132_Elephant_Loxodonta_africana

IFN-β ENSMMSP00000001381_Siberian_musk_deer_Moschus_moschiferus

IFN-β ENSMMSP00000001423_Siberian_musk_deer_Moschus_moschiferus

IFN-β ENSOARP00000009307_Sheep_texel_

IFN-β ENSOARP00000009333_Sheep_texel_

IFN-β ENSOARP00000009432_Sheep_texel_

IFN-β ENSCHIP00000004054_Goat_Capra_hircus

IFN-β ENSCHIP00000006620_Goat_Capra_hircus

IFN-β ENSCHIP00000009453_Goat_Capra_hircus

IFN-β ENSCHIP00000001260_Goat_Capra_hircus

IFN-β ENSCHIP00000008789_Goat_Capra_hircus

IFN-β ENSCHIP00000005465_Goat_Capra_hircus

IFN-β ENSCHIP00000006276_Goat_Capra_hircus

IFN-β ENSCHIP00000001421_Goat_Capra_hircus

IFN-β ENSCHIP00000010698_Goat_Capra_hircus

IFN-β ENSBBBP00000015456_American_bison_Bison_bison_bison

IFN-β ENSBBBP00000009314_American_bison_Bison_bison_bison

IFN-β ENSBBBP00000011523_American_bison_Bison_bison_bison

IFN-β ENSBBBP00000023078_American_bison_Bison_bison_bison

IFN-β ENSBBBP00000023054_American_bison_Bison_bison_bison

IFN-β ENSBMUP00000008356_Wild_yak_Bos_mutus

IFN-β ENSBMUP00000033008_Wild_yak_Bos_mutus

IFN-β ENSBMUP00000008380_Wild_yak_Bos_mutus

IFN-β ENSBMUP00000008339_Wild_yak_Bos_mutus

IFN-β ENSBTAP00000062866_Cow_Bos_taurus

IFN-β ENSBTAP00000062103_Cow_Bos_taurus

IFN-β ENSBTAP00000065352_Cow_Bos_taurus

IFN-β ENSBTAP00000069345_Cow_Bos_taurus

IFN-β ENSBTAP00000063551_Cow_Bos_taurus

IFN-β ENSBTAP00000064555_Cow_Bos_taurus

IFN-β ENSBTAP00000058775_Cow_Bos_taurus

IFN-β ENSBTAP00000067210_Cow_Bos_taurus

IFN-β ENSBTAP00000045034_Cow_Bos_taurus

IFN-β ENSBIXP00005042035_Hybrid_-_Bos_Indicus_Bos_indicus_x_Bos_taurus

IFN-β ENSBIXP00000017895_Hybrid_-_Bos_Indicus_Bos_indicus_x_Bos_taurus

IFN-β ENSBIXP00005022341_Hybrid_-_Bos_Indicus_Bos_indicus_x_Bos_taurus

IFN-β ENSBIXP00005022322_Hybrid_-_Bos_Indicus_Bos_indicus_x_Bos_taurus

IFN-β ENSBIXP00005022360_Hybrid_-_Bos_Indicus_Bos_indicus_x_Bos_taurus

IFN-β ENSBIXP00005014421_Hybrid_-_Bos_Indicus_Bos_indicus_x_Bos_taurus

IFN-β ENSBIXP00000017879_Hybrid_-_Bos_Indicus_Bos_indicus_x_Bos_taurus

IFN-β ENSBIXP00000038066_Hybrid_-_Bos_Indicus_Bos_indicus_x_Bos_taurus

IFN-β ENSBIXP00005041964_Hybrid_-_Bos_Indicus_Bos_indicus_x_Bos_taurus

IFN-β ENSBIXP00005022306_Hybrid_-_Bos_Indicus_Bos_indicus_x_Bos_taurus

IFN-β ENSBIXP00000017883_Hybrid_-_Bos_Indicus_Bos_indicus_x_Bos_taurus

IFN-β ENSBIXP00000038060_Hybrid_-_Bos_Indicus_Bos_indicus_x_Bos_taurus

IFN-β ENSBGRP00000003734_Domestic_yak_Bos_grunniens

IFN-β ENSBGRP00000003643_Domestic_yak_Bos_grunniens

IFN-β ENSBGRP00000003616_Domestic_yak_Bos_grunniens

IFN-β ENSBGRP00000003687_Domestic_yak_Bos_grunniens

IFN-β ENSBGRP00000003653_Domestic_yak_Bos_grunniens

IFN-β ENSBGRP00000003675_Domestic_yak_Bos_grunniens

IFN-β ENSCHYP00000026759_Yarkand_deer_Cervus_hanglu_yarkandensis

IFN-β ENSCHYP00000021331_Yarkand_deer_Cervus_hanglu_yarkandensis

IFN-β ENSCHYP00000035281_Yarkand_deer_Cervus_hanglu_yarkandensis

IFN-β ENSCHYP00000017780_Yarkand_deer_Cervus_hanglu_yarkandensis

IFN-β ENSCHYP00000035243_Yarkand_deer_Cervus_hanglu_yarkandensis

IFN-β ENSBMSP00010000236_Blue_whale_Balaenoptera_musculus

IFN-β ENSPCTP00005022719_Sperm_whale_Physeter_catodon

IFN-β ENSDLEP00000028877_Beluga_whale_Delphinapterus_leucas

IFN-β ENSMMNP00015022013_Narwhal_Monodon_monoceros

IFN-β ENSPSNP00000001378_Vaquita_Phocoena_sinus

IFN-β ENSTTRP00000015564_Dolphin_Tursiops_truncatus

IFN-β ENSCWAP00000016727_Chacoan_peccary_Catagonus_wagneri

IFN-β ENSSSCP00000005550_Pig_Sus_scrofa

IFN-β ENSCDRP00005001879_Arabian_camel_Camelus_dromedarius

IFN-β ENSETEP00000007197_Lesser_hedgehog_tenrec_Echinops_telfairi

IFN-β ENSPCAP00000006984_Hyrax_Procavia_capensis

IFN-β ENSPCAP00000006138_Hyrax_Procavia_capensis

IFN-β ENSOCUP00000020497_Rabbit_Oryctolagus_cuniculus

IFN-β ENSSVLP00005029410_Eurasian_red_squirrel_Sciurus_vulgaris

IFN-β ENSSVLP00005029368_Eurasian_red_squirrel_Sciurus_vulgaris

IFN-β ENSSVLP00005029071_Eurasian_red_squirrel_Sciurus_vulgaris

IFN-β ENSSVLP00005029236_Eurasian_red_squirrel_Sciurus_vulgaris

IFN-β ENSSVLP00005029312_Eurasian_red_squirrel_Sciurus_vulgaris

IFN-β ENSMMMP00000006625_Alpine_marmot_Marmota_marmota_marmota

IFN-β ENSMMMP00000006700_Alpine_marmot_Marmota_marmota_marmota

IFN-β ENSSTOP00000016816_Squirrel_Ictidomys_tridecemlineatus

IFN-β ENSSTOP00000016011_Squirrel_Ictidomys_tridecemlineatus

IFN-β ENSSTOP00000020908_Squirrel_Ictidomys_tridecemlineatus

IFN-β ENSSTOP00000020234_Squirrel_Ictidomys_tridecemlineatus

IFN-β ENSSDAP00000017051_Daurian_ground_squirrel_Spermophilus_dauricus

IFN-β ENSUPAP00010008351_Arctic_ground_squirrel_Urocitellus_parryii

IFN-β ENSUPAP00010024227_Arctic_ground_squirrel_Urocitellus_parryii

IFN-β ENSCAPP00000009742_Brazilian_guinea_pig_Cavia_aperea

IFN-β ENSCPOP00000015789_Guinea_Pig_Cavia_porcellus

IFN-β ENSCPOP00000008736_Guinea_Pig_Cavia_porcellus

IFN-β ENSHGLP00000027652_Naked_mole-rat_female_Heterocephalus_glaber

IFN-β ENSHGLP00000028805_Naked_mole-rat_female_Heterocephalus_glaber

IFN-β ENSHGLP00100027358_Naked_mole-rat_female_Heterocephalus_glaber

IFN-β ENSFDAP00000011502_Damara_mole_rat_Fukomys_damarensis

IFN-β ENSNGAP00000018763_Upper_Galilee_mountains_blind_mole_rat_Nannospalax_galili

IFN-β ENSMSIP00000025914_Steppe_mouse_Mus_spicilegus

IFN-β ENSMUSP00000056720_Mouse_Mus_musculus

IFN-β MGP_SPRETEiJ_P0065394

IFN-β MGP_CAROLIEiJ_P0062911

IFN-β MGP_PahariEiJ_P0078046

IFN-β ENSRNOP00000008225_Rat_Rattus_norvegicus

IFN-β ENSMUGP00000014045_Mongolian_gerbil_Meriones_unguiculatus

IFN-β ENSCGRP00001002020_Chinese_hamster_CHOK1GS_Cricetulus_griseus

IFN-β ENSCGRP00000026463_Chinese_hamster_CHOK1GS_Cricetulus_griseus

IFN-β ENSCGRP00015021979_Chinese_hamster_CHOK1GS_Cricetulus_griseus

IFN-β ENSPEMP00000031000_Northern_American_deer_mouse_Peromyscus_maniculatus_bairdii

IFN-β ENSSARP00000003573_Shrew_Sorex_araneus

IFN-β ENSECAP00000004620_Horse_Equus_caballus

IFN-β ENSECAP00000050798_Horse_Equus_caballus
